# Shifts in frog size and phenology: Testing predictions of climate change on a widespread anuran using data from prior to rapid climate warming

**DOI:** 10.1002/ece3.3636

**Published:** 2017-12-23

**Authors:** Jennifer A. Sheridan, Nicholas M. Caruso, Joseph J. Apodaca, Leslie J. Rissler

**Affiliations:** ^1^ Department of Biological Sciences University of Alabama Tuscaloosa AL USA; ^2^ Division of Science Yale‐NUS College Singapore; ^3^ Tangled Bank Conservation Asheville NC USA; ^4^ Division of Environmental Biology National Science Foundation Arlington VA USA

**Keywords:** amphibians, Bergman's rule, global warming, James’ rule, *Lithobates sylvaticus*, phenology

## Abstract

Changes in body size and breeding phenology have been identified as two major ecological consequences of climate change, yet it remains unclear whether climate acts directly or indirectly on these variables. To better understand the relationship between climate and ecological changes, it is necessary to determine environmental predictors of both size and phenology using data from prior to the onset of rapid climate warming, and then to examine spatially explicit changes in climate, size, and phenology, not just general spatial and temporal trends. We used 100 years of natural history collection data for the wood frog, *Lithobates sylvaticus* with a range >9 million km^2^, and spatially explicit environmental data to determine the best predictors of size and phenology prior to rapid climate warming (1901–1960). We then tested how closely size and phenology changes predicted by those environmental variables reflected actual changes from 1961 to 2000. Size, phenology, and climate all changed as expected (smaller, earlier, and warmer, respectively) at broad spatial scales across the entire study range. However, while spatially explicit changes in climate variables accurately predicted changes in phenology, they did not accurately predict size changes during recent climate change (1961–2000), contrary to expectations from numerous recent studies. Our results suggest that changes in climate are directly linked to observed phenological shifts. However, the mechanisms driving observed body size changes are yet to be determined, given the less straightforward relationship between size and climate factors examined in this study. We recommend that caution be used in “space‐for‐time” studies where measures of a species’ traits at lower latitudes or elevations are considered representative of those under future projected climate conditions. Future studies should aim to determine mechanisms driving trends in phenology and body size, as well as the impact of climate on population density, which may influence body size.

## 1 INTRODUCTION

Understanding how species respond to climate change, both at a population and species level, is one of the most important questions facing ecologists and evolutionary biologists today. Changes in body size (Edeline, Lacroix, Delire, Poulet, & Legendre, 2013; Gardner, Peters, Kearney, Joseph, & Heinsohn, 2011) and breeding phenology (Bradshaw & Holzapfel, 2006; Parmesan, 2006; Stenseth et al., 2002; Walther et al., 2002) have been identified as two of the major ecological consequences of climate change. In many organisms, size dictates energetics and fitness, which in turn affect population size, so the effect of climate on body size has potentially far‐reaching repercussions for individuals, populations, and ecosystems (Edeline et al., 2013; Schuldiner‐Harpaz & Coll, 2013). Similarly, phenological shifts may lead to changes in growth of individuals and populations, and such shifts could cause species interactions to fall out of sync, leading to fitness consequences that are difficult to predict (Parmesan, 2007; Root et al., 2003). Many studies have revealed size declines (Caruso, Sears, Adams, & Lips, 2014; Gardner et al., 2011; Millien et al., 2006; Sheridan & Bickford, 2011) associated with increased temperature across large timescales for several taxa, but the direction and magnitude of size shifts are not consistent across all species within a taxonomic group (e.g., birds) or across populations of a given species (Gardner et al., 2011, 2014; Salewski, Siebenrock, Hochachka, Woog, & Fiedler, 2014; Yom‐Tov & Yom‐Tov, 2005).

Documented size declines are often cited as reflections of biogeographical or macroecological patterns such as Bergmann's rule (warmer environments tend to be inhabited by smaller endothermic species than colder environments, Blackburn, Gaston, & Loder, 1999; Clauss, Dittmann, Muller, Meloro, & Codron, 2013) or James’ rule (within a given species, individuals in warmer environments are smaller than those in colder environments, James, 1970). Additionally, many species are known to have decreased in size during past periods of climate warming (Finkel, Katz, Wright, Schofield, & Falkowski, 2005; Hadly, Kohn, Leonard, & Wayne, 1998; Smith, Betancourt, & Brown, 1995; Smith, Hasiotis, Kraus, & Woody, 2009) or in response to experimental warming of their environment (Chomsky, Kamenir, Hyams, Dubinsky, & Chadwick‐Furman, 2004; Hovenden et al., 2008; de Jong, 2010; Stillwell & Fox, 2009). While body size is certainly due to a number of selective factors such as evolutionary history and population density, among many others, these lines of evidence suggest that temperature, and its physiological effects on organisms, is one of the most important driving mechanisms behind observed size changes, and researchers have proposed that recent trends in size declines are due to temperature's influence on metabolism (Bickford, Howard, Ng, & Sheridan, 2010), developmental rate (Gillooly, Brown, West, Savage, & Charnov, 2001), or resource availability (Wikelski & Thom, 2000). However, empirical patterns found in nature vary (Chamaille‐Jammes, Massot, Aragon, & Clobert, 2006), and there have been few empirical tests of whether observed changes in size and breeding phenology are due directly to climate changes.

Determining the mechanisms influencing body size in wild populations over long time spans can be difficult, but any given mechanism will have predictable outcomes associated with observed climate changes. If the mechanism is heat preservation, for example, as proposed for Bergmann's rule, we would expect declines in body size with increased temperatures. If the mechanism is minimization of water loss or desiccation, we would expect size increases with decreased precipitation. Alternately, if the mechanism is food abundance or quality, we would expect increased size with increased feeding season length or primary productivity (Gardner et al., 2014).

Additionally, if climate is directly responsible for observed changes in size or phenology, then climate data from prior to the onset of rapid climate warming should determine the statistical relationship between climate and both size and phenology. That information could then be used to predict size and phenology changes across space and time. If observed patterns postclimate warming are in line with predictions, then climate is strongly implicated in directly causing these shifts. Alternatively, if recent observed shifts in size and phenology do not match predictions, then mechanisms other than climate, such as resource availability or quality, are likely more important than direct effects of climate change.

**Figure 1:**
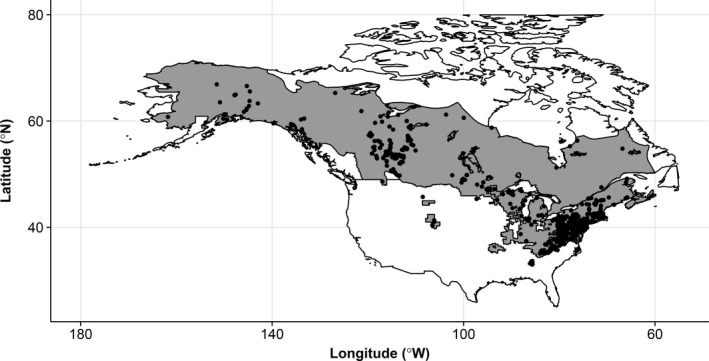
Map of wood frog range (gray) with sampling locations (black dots) across North America

Limited sampling of most species across time and space (e.g., vouchers in natural history collections), coupled with limited geographic ranges of species, makes assessing body size and phenology shifts due to climate change challenging for all but relatively few taxa. In North America, the wood frog (*Lithobates sylvaticus*) provides one such rare opportunity. The species is well represented in natural history collections; has the largest range of any amphibian on the continent (~10 million km^2^); spans 35°–65°N latitude; and has a latitudinal gradient in body size that follows the converse of James’ rule (i.e., larger in the south, smaller in the north, Angilletta & Dunham, 2003; Davenport & Hossack, 2016; Martof & Humphries, 1959), hypothesized to reflect the greater number of available feeding days in the south, leading to larger body sizes there compared to more northern latitudes (Martof & Humphries, 1959). Interestingly, studies by Berven (1982) of the same species across elevational rather than latitudinal gradients demonstrated that animals from higher (colder) elevations were larger than those at lower (warmer) elevations. Together, these studies indicate that mechanisms operating at small (e.g., within a mountain range across elevations) and large (e.g., continental) scales may differ or that our conclusions as to the mechanisms depend on scale. In this study, we quantify the relationship between climate and both body size and phenology of wood frogs across their entire range (Figure 1) from prior to rapid climate warming (1901–1960) using a spatially explicit approach. We then test whether those relationships are maintained during rapid climate warming (1961–2000), in order to determine whether climate is likely to be acting directly or indirectly on size and breeding phenology.

## 2 METHODS

We present data on 1,264 sexually mature (484 gravid females and 780 males with nuptial pads) wood frog specimens, collected between 1901 and 2000, to determine spatial and temporal patterns of body size and breeding phenology (Table S1). While there are many more available *L. sylvaticus* specimens in museum collections than in our dataset, we limited our measurements to those which were sexually mature, and which had exact collection locality information, in order to perform spatially explicit analyses. We compared body size and Julian day of first collection (a proxy for breeding date), for two periods: 1901–1960 (body size *n* = 679; phenology *n* = 359) and 1961–2000 (body size *n* = 585; phenology *n* = 414) as there were insufficient sample sizes to analyze changes on a decade‐by‐decade basis (Table S2). We chose this split because the decade spanning 1950–1960 is approximately when global climate begins to shift drastically in the historical record (Girvetz et al., 2009). Individuals were measured for snout–vent length (SVL) to the nearest 0.05 mm with digital calipers by a single researcher (JAS) to eliminate interindividual measurement variation (Bernal & Clavijo, 2009; Lee, 1982). Date of collection was converted to Julian day to analyze phenology shifts. Because wood frogs have explosive breeding events, with reports showing up to 80% of breeding occurring within 3 days, for example (Petranka & Thomas, 1995; Waldman, 1982), and because they are typically collected during the breeding season, we considered Julian day of collection to be synonymous with breeding events. Although we know of no study which has previously used collection date as a proxy for breeding date in frogs, collection date has been used to measure change in phenology for other taxa (Davis, Willis, Connolly, Kelly, & Ellison, 2015; Scharlemann, 2001). Additionally, given that male and female wood frogs arrive at breeding sites within a few days of each other (Howard, 1980; Waldman, 1982), we believe collection date combined with natural history knowledge can be used as a proxy for breeding in this species. We identified some collections that were well outside of the breeding season (determined at any given latitude by amphibian field guides, natural history publications on this species, and our own experience in the field) so we visually inspected histograms and used k‐means clustering to identify two clusters of collection dates (breeding season and nonbreeding season) within each degree of latitude for which we had data. Dates separate from the breeding season cluster were removed from phenology analyses.

### 2.1 Climate data

Historical climate data were downloaded from the Intergovernmental Panel on Climate Change (IPCC) Data Distribution Centre (DDC) and were manipulated using ArcGIS^®^ (V 10.2) software by Esri to create decadal layers of nontransformed (true‐value) climate variables (Mitchell, Carter, Jones, Hulme, & New, 2004; Mitchell & Jones, 2005). Our climate dataset consists of mean temperature, precipitation, and number of frost‐free days (FFD) for each decade of the time period 1901–1960 or 1961–2000, at five arc‐minute resolution.

Within our dataset, both temperature and FFD were highly correlated (*r* = .873; *t*_1,262_ = 63.570; *p* < .001), while precipitation was weakly correlated with both temperature (*r* = .320; *t*_1,262_ = 11.995; *p* < .001) and FFD (*r* = .245; *t*_1,262_ = 8.980; *p* < .001). Running models with both precipitation and temperature change resulted in very high (>>5) variance inflation factor (a measure of multicollinearity), whereas models run with precipitation and FFD change had low variance inflation factor, so we chose FFD over temperature for subsequent analyses where appropriate (Table S3).

### 2.2 Climate variables associated with historic body size and breeding dates (1901–1960)

We used data from 1901 to 1960 to determine whether FFD, precipitation, or their interaction (FFD:precipitation) best explains spatial patterns in body size and breeding date throughout the species’ range. To confirm our choice of cutoff date, we tested whether the relationship between climate variables and body size (female or male) or collection date varied with choice of cutoff date, comparing our model selection (described below) and coefficient values with 95% CI for eight different cutoff years (every 5 years from 1940 to 1980), and compared decadal coefficient estimates (1930, 1940, 1950, 1960, 1970, 1980) to our 1960 cutoff date estimates. We found no effect of choice of cutoff date on our results and no consistent trend in observations when compared on a decade‐by‐decade basis (Appendix S1). To account for sexual dimorphism in this species (Berven, 1982), we analyzed male and female body size data separately. Data for both males and females were used together for phenology analysis as both sexes are known to arrive at breeding ponds within one to a few days of each other (Howard, 1980; Waldman, 1982).

We used a linear mixed effects model with an exponential spatial correlation structure that included each data point's latitude, longitude, and elevation to correct for spatial autocorrelation. We modeled, separately, the responses of SVL (log‐transformed) and Julian day, with the year of collection as a random intercept and fit five models to include all possible combinations of FFD, precipitation, and their interaction as well as an intercept‐only model (Table S4). Both FFD and precipitation were centered and scaled prior to analyses. We fit all models using maximum likelihood and determined the best model for SVL and Julian day based on lowest AICc (Burnham & Anderson, 2002). We confirmed that all our models met the assumptions of homogeneity of variances, normally distributed residuals, and linearity by inspecting plots of standardized residuals versus fitted values, quantile–quantile plots, and plots of our three response variables against our fixed effects, respectively. For the model with the lowest AICc, we determined significance of the fixed effect using a likelihood ratio test. When multiple models received substantial support (i.e., ΔAICc ≤ 2), we averaged competing models and determined significance based on whether the 95% confidence intervals overlapped with zero. We report the marginal and conditional *R* ^2^ values of all models tested (Table S4), where marginal *R* ^2^ (*R* ^2^ *m*) is a measure of the proportion of the variance explained by the fixed effects, and the conditional *R* ^2^ (*R* ^2^ *c*) measures the proportion of the variance explained by both the random and fixed effects (Nakagawa & Schielzeth, 2013). We tested each parameter (temperature, FFD, FFD+precipitation, etc.) independently to determine whether it was significantly related to body size or Julian day of collection (breeding).

### 2.3 Temporal changes in body size and breeding date independent of climate

For pre‐1960 and post‐1960 data separately, we used a thin plate spline regression in which the response of SVL or Julian day was regressed against the latitude, longitude, and elevation of each data point, and we used 100 bootstrapped iterations to determine the mean body size or mean Julian day and 95% confidence intervals for pre‐ and post‐1960. Additionally, we used the bootstrapped data to determine the mean *r* ^2^ and the mean‐squared prediction error to determine prediction accuracy. We created predictive and spatially explicit maps of our two response variables pre‐ and post‐1960 using five arc‐minute resolution raster layers of latitude, longitude, and elevation, so that all areas within the species’ range had an estimated body size and breeding phenology value. We obtained 95% confidence intervals for each map as some geographic regions were more heavily sampled (e.g., Appalachia) and some were not sampled (e.g., areas in northwestern Canada) (Figure 1). Those geographic areas in which the confidence intervals pre‐ and post‐1960 did not overlap were determined to be significant areas of change, while areas in which the confidence intervals overlapped were considered equal (i.e., change equals zero).

Given the lack of consistent sampling across time at all geographic locations, as is typical of natural history collections, this method allowed a more conservative estimate across the entire continental range for those areas that showed changes in body size and breeding phenology. Analysis of covariance (ANCOVA) in which the continuous predictor is a spatial variable (e.g., latitude) and the categorical predictor is pre‐ or post‐1960 is less preferred because we do not have equal sampling across latitudes, longitudes, or elevations (Figure S1 and Table S2). Because the wood frog has known body size and phenology clines with latitude and elevation (Berven, 1982; Davenport & Hossack, 2016; Martof & Humphries, 1959), an uneven distribution of samples (e.g., among elevations and across latitudes) between pre‐ and post‐1960 would bias our results using an ANCOVA. Similar to our analyses with climate variables, we determined the sensitivity of our analyses to our cutoff date (1960), but found no strong differences in patterns based on choice of cutoff date, so we present here results only using our original cutoff date of 1960 (Appendix S1).

### 2.4 Changes in body size and breeding date with climate

We used a general least‐squares model with an exponential spatial correlation structure to determine the relationship between the change in body size or Julian day and changes in climate across our two study periods. First, we created a dataset of 484 randomly distributed points (equivalent to our smallest dataset, female body size). Next, we used those data points to extract the spatially explicit data on mean change in body size (males and females) and Julian day, as well as the two climate variables. Both fixed effects were adjusted such that the data were proportional to the pre‐1960 levels (e.g., Δ precipitation/pre‐1960 precipitation). We weighted each response (female size, male size, Julian day) by its pooled standard error (i.e., areas with low pooled standard error were weighted more than areas with high pooled standard error), and fit each of our three models using maximum likelihood. We determined significance of each fixed effect by examining 95% confidence intervals of averaged models. Because our responses did not show a linear relationship with our fixed effects, we fit both fixed effects as second‐order polynomials and tested all combinations of our fixed effects for all responses (linear terms were always included when quadratic terms were included).

All statistical analyses were performed using R version 3.3.1 (R Core Team, 2015); we used the *classInt* package for *k*‐means clustering (Bivand, 2015), the *fields* package for thin plate spline regressions (Nychka, Furrer, Paige, & Sain, 2015), the *nlme* package for linear mixed effects and general least‐squares models (Pinheiro, Bates, DebRoy, Sarkar, & Team, 2013), and the *MuMIn* package (Barton, 2016) for AICc model selection and determining marginal and conditional *R* ^2^ values.

## 3 RESULTS

### 3.1 Climate variables associated with historic (1901–1960) body size and breeding dates

Two models (precipitation and FFD*precipitation) provided the best explanation for variation in female body size (Table S5) from 1901 to 1960. Female body size was positively associated with precipitation (slope = 0.112; 95% CI = 0.088, 0.136; χ^2^ = 15.401; *p* < .001; *R* ^2^ *c* = 0.423) and the interaction between FFD and precipitation (slope = 0.045; 95% CI = 0.023, 0.067; χ^2^ = 15.401; *p* < .001; *R* ^2^ *c* = 0.429; Table S5).

Male body size was positively associated with FFD (slope = 0.041; 95% CI = 0.024, 0.057; *R* ^2^ *c* = 0.205) but not with precipitation (slope = 0.008; 95% CI = −0.002, 0.018; *R* ^2^ *c* = 0.173) or FFD:precipitation (slope = −0.004; 95% CI = −0.014, 0.007; *R* ^2^ *c* = 0.190; Table S5).

Julian day of collection was negatively associated with FFD (slope = −42.403; 95% CI = 47.611, 37.195; *R* ^2^ *c* = 0.714) and precipitation (slope = −1.219; 95% CI = −2.260, −0.179; *R* ^2^ *c* = 0.427) at a given latitude, but was not significantly associated with FFD*precipitation (slope = 0.377; 95% CI = −0.736, 1.490; *R* ^2^ *c* = 0.719; Table S5).

### 3.2 Temporal changes in body size and breeding date independent of climate

**Figure 2:**
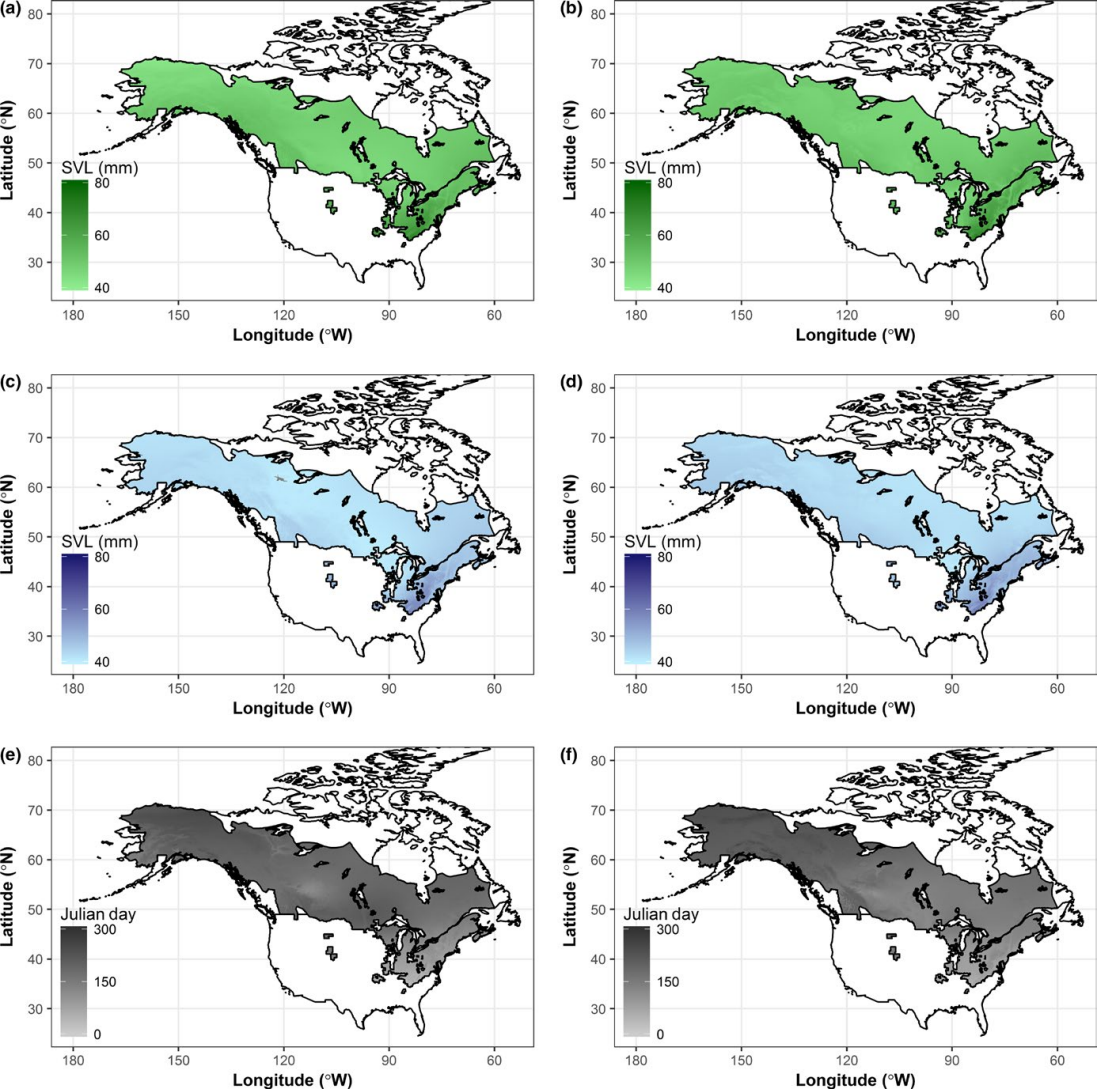
Body size of females before (a) and after (b) 1960; body size of males before (c) and after (d) 1960; Julian day of first collection (breeding) before (e) and after (f) 1960

**Figure 3:**
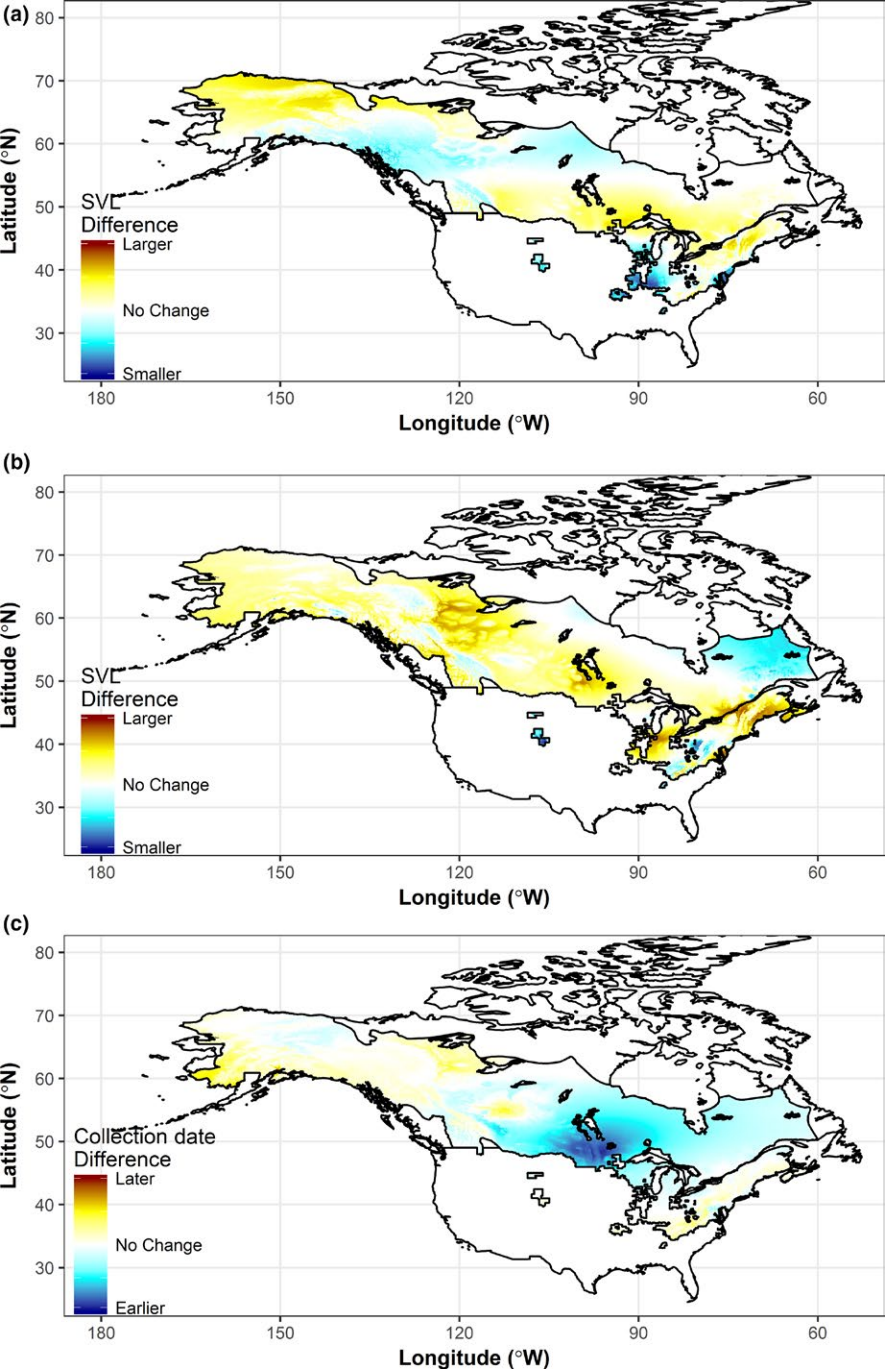
Change between the two study periods, 1901–1960 and 1961–2000, for (a) female body size, (b) male body size, and (c) Julian day of first collection (breeding)

After 1960, we found that size of both sexes increased and decreased, depending on locality and that these changes were not uniform across the species’ range (Figures 2 and 3). Similarly, breeding dates were earlier in most parts of the range, but later in the Appalachians, far northwest Canada, and Alaskan coastal areas (Figure 3c).

### 3.3 Changes in body size and breeding date in relation to climate changes

**Figure 4:**
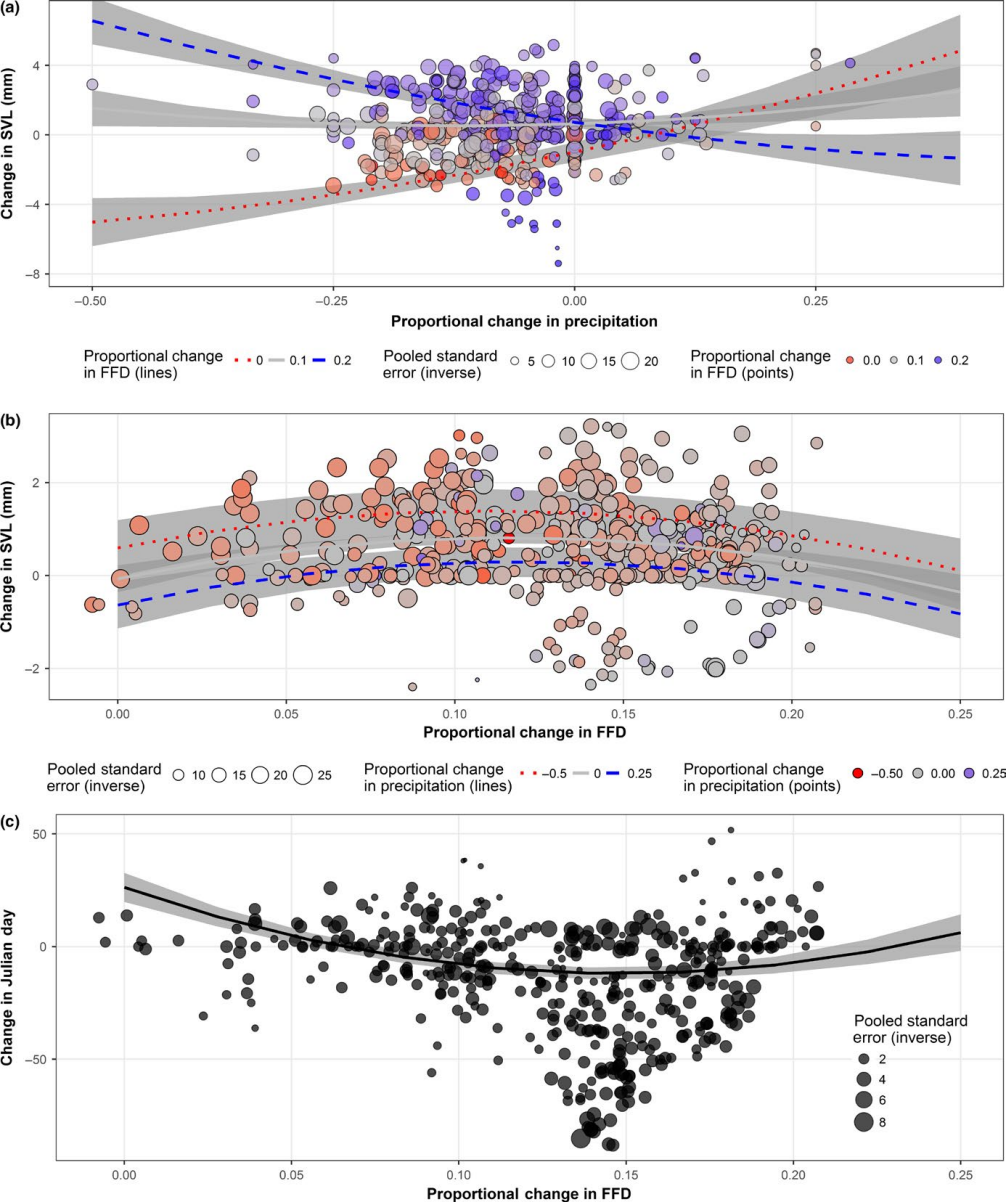
Change in (a) female body size, (b) male body size, (c) Julian day of first collection (breeding) with respect to change in climate. In panel a, the three lines represent low (red dashed line), median (thin gray line), and high (blue dashed line) changes in FFD. In panel b, the three lines represent low (red dashed line), median (thin gray line), and high (blue dashed line) changes in precipitation. Panel c contains a single line because there were no mixed effects, and the gray‐shaded ribbons represent 95% CI. Color of dots corresponds to associated line (red, gray, or blue), and size of dots is proportional to the inverse of the pooled standard error (smaller dots have higher *SE*; see Methods)

We found that the variation in change in female body size was dependent on FFD, precipitation, and their interaction (Table 1). In areas with no proportional change in FFD (Figure 4a, red dotted line), female body size decreased in areas which also became drier (proportional change in precipitation < 0; Figure 4a) or had small proportional increases in precipitation, but female body size increased in areas where proportional increases in precipitation were high. In areas with an intermediate increase in FFD, females always became larger regardless of precipitation change (thin gray line, Figure 4a). In areas with high proportional increases in FFD (Figure 4a, blue dashed line), female body size increased in areas which also became drier (proportional change in precipitation < 0; Figure 4a) or had small proportional increases in precipitation, but decreased in areas with high proportional increases in precipitation. Thus, females were larger in areas with small increases in FFD regardless of precipitation, as well as in areas with intermediate and high increases in FFD that also became drier or experienced only small increases in precipitation. Females became smaller in areas with no change in FFD that also were drier or only experienced small increases in precipitation, and in areas that experienced relatively large increases in both FFD and precipitation.

**Table 1:** Model estimates for relationship between change in climate variables and change in female body size, male body size, and Julian day of first collection (breeding)

	Fixed effect	Estimate	Lower 95% CI	Upper 95% CI
Female body size	**FFD**	**23.87**	**6.69**	**41.06**
	**FFD** ^**2**^	**−76.39**	**−143.68**	**−9.09**
	**Precipitation**	**11.67**	**6.11**	**17.24**
	**Precipitation** ^**2**^	**9.95**	**0.43**	**19.46**
	**FFD:Precipitation**	**−98.70**	**−138.55**	**−58.84**
Male body size	**FFD**	**15.24**	**6.12**	**24.35**
	**FFD** ^**2**^	**−65.57**	**−103.68**	**27.46**
	**Precipitation**	**−1.95**	**−3.86**	**−0.04**
	Precipitation^2^	**−**3.69	**−**9.76	2.38
	FFD:Precipitation	**−**7.76	**−**16.54	32.06
Julian day of first collection (breeding)	**FFD**	**−511.88**	**−726.35**	**−297.42**
	**FFD** ^**2**^	**1,744.72**	**901.43**	**2,588.01**
	Precipitation	27.65	**−**32.01	87.32
	Precipitation^2^	98.51	**−**53.73	250.75
	FFD:Precipitation	230.10	**−**336.63	796.83

Bolded rows indicate significance (upper and lower CI overlap zero).

Changes in male body size were significantly explained by the second‐order polynomial of FFD and precipitation (Table 1). Areas which were drier (red dotted line, Figure 4b) saw males increase in size, regardless of the change in FFD, but the greatest size increases occurred in areas with median levels of proportional changes in FFD (Figure 4b). Areas with no change in precipitation (thin gray line, Figure 4b) also saw males increase in size, except in areas with the highest proportional increase in FFD, where males decreased in size. Areas with increases in precipitation (blue dashed line, Figure 4b) saw males increase in size only when there was also an intermediate change in proportional FFD, and decrease in areas where there was a very low or very high level of change in proportional FFD (Figure 4b). Thus, males were larger in areas that were drier, areas with no change in precipitation and low to intermediate increases in FFD, and areas that were wetter with an intermediate increase in FFD. Males were smaller in areas with very high proportional increases in FFD that also had zero or positive changes in precipitation, as well as in areas that were wetter with very low increases in number of FFD.

Lastly, the second‐order polynomial of FFD significantly explained changes in Julian day (Table 1): Earlier first collection dates at a given location were associated with intermediate proportional increases in FFD (Figure 4c), and later first collection dates were associated with very low and very high proportional increases in FFD.

## 4 DISCUSSION

As expected, size and Julian day of first collection (breeding) changed significantly across our two time periods. However, the climate factor most strongly associated with each of our response variables before onset of rapid climate warming did not maintain the same relationship after the onset of climate change. Additionally, the strongest climate predictor/s of size differed for males and females, and the relationship between size and climate variables was complex. Thus, when data from all locations were pooled in analyses, we found that both males and females were larger after 1960 compared to before, but examination of changes at fine spatial scales (10 km^2^) revealed that size increased in some areas and decreased in others, depending on the specific temperature and precipitation changes. This is an important result because most studies do not examine species across such a large geographic range nor with spatially explicit estimates of climate or trait changes (but see Davenport & Hossack, 2016). Given that precipitation changes are expected to be difficult to predict (IPCC, 2013), our results suggest that size changes of wood frogs may not be easy to anticipate and that caution should be used when ecologists and evolutionary biologists use “space‐for‐time” studies (Pickett, 1989) where measures of a species’ traits at lower latitudes or elevations are considered representative of those under future projected climatic conditions.

We were unable to identify a single mechanism (heat preservation, minimization of water loss, food abundance) responsible for size change in wood frogs, due in part to the complex relationship between size and climate variables we observed. For example, our results suggest that male size may be driven by minimization of water loss when areas become drier (larger size), but that with no change or increase in precipitation, size is more strongly influenced by food abundance (larger with low to intermediate increased FFD) and heat preservation (smaller with very high increases in FFD). Notably, females responded to precipitation changes in the opposite direction as that predicted by the mechanism of minimizing water loss when FFD did not change. Instead of becoming larger in drier areas and smaller in wetter areas, females became smaller and larger, respectively. This may suggest that food abundance is instead the driving mechanism, as food abundance is ultimately influenced by changes in precipitation (more primary productivity in wetter areas, less in drier areas). This is supported by the larger female sizes observed with small increases in FFD, as predicted when food abundance is the driving mechanism of body size. However, with intermediate and high increases in FFD, females became larger with decreased precipitation but smaller with increased precipitation, indicating that food abundance is not the strongest predictor of size in these cases, and that instead, minimization of water loss may be the driving mechanism.

At broad spatial scales, overall patterns and degree of size change in our study are similar to those found for several other terrestrial ectotherms examined so far (McCarthy, Masson, Thieme, Leimgruber, & Gratwicke, 2017; Tryjanowski, Sparks, Rybacki, & Berger, 2006; Wikelski & Thom, 2000). Two species of European frogs, for example, increased in size from 1963 to 2003 (Tryjanowski et al., 2006) in conjunction with milder winters, and the authors suggest that warmer winters may have increased the number of insect prey, resulting in higher growth rates of these species. Size of marine iguanas and at least one population of salamanders are also known to be correlated with resource availability; both species were reported to have shrunk during periods of decreased food availability (Bendik & Gluesenkamp, 2013; Wikelski & Thom, 2000). However, in our study, size increases were not greatest in areas with the highest increases in frost‐free days (a proxy for number of feeding days), nor in areas that were both warmer and wetter, which should lead to increased primary productivity. Thus, resource availability may not always be the driving mechanism for size changes in wood frogs, as has been suggested for other species (Yom‐Tov & Yom‐Tov, 2004; Yom‐Tov, Yom‐Tov, & Jarrell, 2008), but explicit tests of stomach contents in museum specimens over time would help directly address this. It is possible that frost‐free days do not correlate directly with higher prey availability, so future studies should also examine changes in primary productivity with respect to body size, as suggested by Huston and Wolverton (2011) and as examined in Gardner et al. (2014). Studies that investigate the interrelationship between temperature and precipitation on resource availability would be informative because changes in rainfall will be less consistent and predictable than temperature, and may vary over even finer spatial and temporal scales (IPCC, 2013).

Our observation that breeding has shifted earlier over time, in conjunction with intermediate increases in number of FFD, is consistent with other observations showing earlier breeding in response to warmer temperatures (Blaustein et al., 2001; Green, 2017), including a study on wood frogs from Michigan which showed a negative association between average winter daily maximum temperature and breeding date between 2006 and 2012 (Benard, 2015). Additionally, our result is consistent with a recent meta‐analysis (Ficetola & Maiorano, 2016), indicating that temperature (here, FFD) and not precipitation is the main driver of phenology trends.

Although we found a significant relationship between body size and climate variables prior to significant climate shifts, those relationships were weak (*R* ^2^ *m* = 0.396 and 0.087). This could be due, in part, to our limited samples at various points in the wood frog's range, as our samples were biased toward the eastern United States. In contrast, the relationship between breeding date and temperature (FFD) was stronger (*R* ^2^ *m* = 0.643) than that for size and temperature, and changes in breeding and frost‐free days from 1961 to 2000 were strongly correlated. While our general trends (body size changes concurrent with changes in climate variables) reflect widely observed patterns, our analyses are unique in making spatially explicit predictions of how body size and phenology are expected to change, based on observed correlations with climate variables prior to rapid climate change.

It should be noted that the correlation of size and phenology with climate variables prior to the onset of rapid climate warming does not necessarily indicate that climate is driving those trends. Both size and phenology are selected for by numerous factors which may themselves be influenced by or correlated with climate. One interpretation of our results could be that climate is not the main driver of body size in wood frogs, as the two were significantly correlated prior to 1960, but not after. If climate were the main driver, the relationship should hold true both before and after the onset of rapid climate warming. For phenology, the fact that we found a significant relationship between Julian date of first collection (breeding) and FFD in both time periods indicates that either the selective factor(s) acting on phenology are changing consistently with FFD or that FFD itself is indeed driving that trend.

One factor that may be responsible for the inconsistent trends of size and breeding changes is differences in population density leading to differences in competition across populations. High population density has been shown to have negative effects on growth in this species (Berven, 2009), and others have found that toads in Ontario increased in size in inverse proportion to density, but that size was unrelated to temperature (Green & Middleton, 2013). Changes in population density surely will contribute to changes in size, but the question remains whether these populations are undergoing long‐term trends in population density (becoming either more or less dense), and what is driving that trend. Without population density studies, it is impossible to answer this question, although data from Berven's long‐term (21 years) study in Michigan (Berven, 2009) could be analyzed with respect to the environmental factors considered here to help establish the relationship between climate, density, and size. Environmental conditions likely contribute to density by affecting resource availability, but density could also be impacted by changes in predator densities or human disturbance, so further studies on size changes of species should also consider factors related to population density when considering mechanisms for size changes.

It may also be that responses to climate in this geographically widespread species vary by genetic clade. Wood frogs are known to have undergone postglacial expansion into much of Canada from various refugia, and Lee‐Yaw, Irwin, and Green (2008) found evidence for two mtDNA clades, one originating in Wisconsin and expanding into the great lakes region, Alaska, and central and western Canada, and one originating in the Appalachians expanding into Maine and eastern Canada. Our observed differences in size and phenology changes do not match up to these mtDNA groupings (see fig. 6 in Lee‐Yaw et al., 2008), but future studies could examine variation in genetics, breeding, and phenology across the wood frog range to determine the relationship between these factors.

As with all studies, possible observation error or collection bias should be considered when interpreting our results. For example, if there is inconsistent observer bias over time toward collecting individuals of a given size, or at a time of year when individuals of a certain size class are more likely to be found (Connette, Crawford, & Peterman, 2015), body size of museum specimens may not be representative of sizes found in nature (Grant, 2015). This could then be one reason why our results are inconsistent with predicted changes in size with environmental changes, so our data may in fact be missing a trend that actually exists.

Similarly, while wood frog breeding is indeed explosive, with reports from the literature indicating that up to 80% of breeding occurring within 3 days, for example (Petranka & Thomas, 1995; Waldman, 1982), the arrival of individuals at a breeding site may occur for many more days before actual breeding. While there is no indication in the literature that arrival before breeding varies across the range of this species, it is possible that first collection of wood frogs in a given year (or over many years) does not match up to actual onset of breeding, and thus, that our results are due to consistently earlier collection efforts over time. The possible influences of collection or observer bias on our results should not be dismissed, but our very large (>1,200 observations) dataset spanning 100 years should be fairly robust to such factors, minimizing such bias.

Our results highlight the fact that while it is easy to infer causation from widely reported correlations between climate and size, the mechanisms behind observed size changes have yet to be identified. Ours is the first study to correlate breeding phenology and size of organisms with environmental variables (temperature, precipitation, number of frost‐free days) *before* significant climate change, and then to test how changes in the strongest environmental predictor of these variables correlate with observed size changes. In addition, because climate (e.g., temperature and precipitation) does not vary across latitude in a simple way, large spatial‐scale analyses typical of macroecological studies are likely to miss variation that is important when assessing mechanisms; therefore, our statistical analyses and spatially explicit predictive maps generated from georeferenced natural history collection data and 10 km^2^ resolution environmental data are an important advance to study trait changes through time and space. Our results do not support the predictions that heat preservation, desiccation prevention, or food abundance alone are the mechanisms responsible for size changes in wood frogs, and we encourage further research in physiological ecology and population density changes with climate to better understand how body size and associated life history traits within and across populations may shift under climate change.

## CONFLICT OF INTEREST

None declared.

## AUTHOR CONTRIBUTIONS

Sheridan conceived of the study, measured all animals and obtained breeding phenology data, and framed the final introduction and discussion. Caruso conducted the statistical analyses and designed the phenology study. Apodaca conducted the geospatial analyses of climate data. Rissler organized the project in association with National Science Foundation grant BCS 1026841 and wrote much of the first draft of this article. All authors edited the final draft of the manuscript.

## DATA ACCESSIBILITY

All anuran locality info is available on Vertnet.org, and specimens can be requested directly from their respective museums. All historical environmental data layers are available in raw form the Intergovernmental Panel on Climate Change (IPCC) Data Distribution Center (http://www.ipcc-data.org).
