# Supplementary material for: Shifts in frog size and phenology: Testing predictions of climate change on a widespread anuran using data from prior to rapid climate warming
Source: Ecol Evol. 2017 Dec 23;8(2):1316–27. doi: 10.1002/ece3.3636 (PMC5773303; doi:10.1002/ece3.3636)
Supplement: Supplementary file 1 [file ECE3-8-1316-s001.docx]

**Supporting Information**

**Table S1.** Specimens used in this study.

| **Museum** | **Catalog No.** | **Species** | **Sex** | **Year Collected** | **Longitude** | **Latitude** |
| --- | --- | --- | --- | --- | --- | --- |
| Auburn | 18192 | *Lithobates sylvaticus* | Male | 1969 | -77.2197 | 38.7963 |
| Auburn | 18193 | *Lithobates sylvaticus* | Male | 1969 | -77.2197 | 38.7963 |
| Auburn | 18194 | *Lithobates sylvaticus* | Male | 1969 | -77.2197 | 38.7963 |
| Auburn | 18195 | *Lithobates sylvaticus* | Male | 1969 | -77.2197 | 38.7963 |
| Auburn | 18196 | *Lithobates sylvaticus* | Male | 1969 | -77.2197 | 38.7963 |
| Auburn | 19302 | *Lithobates sylvaticus* | Female | 1971 | -79.0000 | 37.0000 |
| Auburn | 19487 | *Lithobates sylvaticus* | Male | 1971 | -79.0000 | 37.0000 |
| Auburn | 24235 | *Lithobates sylvaticus* | Male | 1976 | -83.3333 | 35.5667 |
| Auburn | 26666 | *Lithobates sylvaticus* | Female | 1978 | -74.0000 | 41.0000 |
| Auburn | 26669 | *Lithobates sylvaticus* | Female | 1978 | -80.3833 | 35.7167 |
| Auburn | 27694 | *Lithobates sylvaticus* | Female | 1979 | -74.5000 | 40.9833 |
| Auburn | 29920 | *Lithobates sylvaticus* | Male | 1981 | -85.8418 | 33.1659 |
| Auburn | 29921 | *Lithobates sylvaticus* | Male | 1981 | -85.8418 | 33.1659 |
| Auburn | 29922 | *Lithobates sylvaticus* | Male | 1981 | -85.8418 | 33.1659 |
| Auburn | 29923 | *Lithobates sylvaticus* | Male | 1981 | -85.8418 | 33.1659 |
| Auburn | 29924 | *Lithobates sylvaticus* | Male | 1981 | -85.8418 | 33.1659 |
| Auburn | 29925 | *Lithobates sylvaticus* | Male | 1981 | -85.8418 | 33.1659 |
| Auburn | 29926 | *Lithobates sylvaticus* | Male | 1981 | -85.8418 | 33.1659 |
| Auburn | 30392 | *Lithobates sylvaticus* | Male | 1980 | -85.8418 | 33.1659 |
| Auburn | 30393 | *Lithobates sylvaticus* | Male | 1980 | -85.8418 | 33.1659 |
| Auburn | 30394 | *Lithobates sylvaticus* | Male | 1980 | -85.8418 | 33.1659 |
| Auburn | 30395 | *Lithobates sylvaticus* | Male | 1980 | -85.8418 | 33.1659 |
| Auburn | 30396 | *Lithobates sylvaticus* | Male | 1980 | -85.8418 | 33.1659 |
| Auburn | 30397 | *Lithobates sylvaticus* | Male | 1980 | -85.8418 | 33.1659 |
| Auburn | 30398 | *Lithobates sylvaticus* | Male | 1980 | -85.8418 | 33.1659 |
| Auburn | 30399 | *Lithobates sylvaticus* | Female | 1979 | -85.8091 | 33.4857 |
| Auburn | 30401 | *Lithobates sylvaticus* | Male | 1980 | -85.8418 | 33.1659 |
| Auburn | 30403 | *Lithobates sylvaticus* | Male | 1980 | -85.6438 | 33.0536 |
| Auburn | 30404 | *Lithobates sylvaticus* | Male | 1980 | -85.6438 | 33.0536 |
| Auburn | 30405 | *Lithobates sylvaticus* | Male | 1980 | -85.6438 | 33.0536 |
| Auburn | 30857 | *Lithobates sylvaticus* | Male | 1982 | -86.0433 | 33.1920 |
| Auburn | 30890 | *Lithobates sylvaticus* | Male | 1982 | -85.8418 | 33.1659 |
| Auburn | 30891 | *Lithobates sylvaticus* | Male | 1982 | -85.8418 | 33.1659 |
| Auburn | 30892 | *Lithobates sylvaticus* | Male | 1982 | -85.8418 | 33.1659 |
| Auburn | 30893 | *Lithobates sylvaticus* | Male | 1982 | -85.8418 | 33.1659 |
| Auburn | 30894 | *Lithobates sylvaticus* | Female | 1982 | -85.8418 | 33.1659 |
| Auburn | 30895 | *Lithobates sylvaticus* | Male | 1982 | -85.8418 | 33.1659 |
| Auburn | 30993 | *Lithobates sylvaticus* | Female | 1979 | -85.7522 | 33.5200 |
| Auburn | 30994 | *Lithobates sylvaticus* | Male | 1979 | -85.8642 | 33.4746 |
| Auburn | 31777 | *Lithobates sylvaticus* | Female | 1979 | -85.6026 | 33.0724 |
| Auburn | 31778 | *Lithobates sylvaticus* | Male | 1979 | -85.7042 | 33.0168 |
| Auburn | 31779 | *Lithobates sylvaticus* | Female | 1979 | -85.6343 | 33.5187 |
| Auburn | 32086 | *Lithobates sylvaticus* | Female | 1983 | -85.6992 | 33.0285 |
| Auburn | 35034 | *Lithobates sylvaticus* | Female | 1997 | -85.6119 | 33.4485 |
| CAS | 2741 | *Lithobates sylvaticus* | Female | 1931 | -153.8836 | 59.7444 |
| CAS | 2743 | *Lithobates sylvaticus* | Male | 1931 | -145.8857 | 61.7380 |
| CAS | 5523 | *Lithobates sylvaticus* | Female | 1939 | -80.9689 | 41.5250 |
| CAS | 6995 | *Lithobates sylvaticus* | Female | 1941 | -71.1715 | 45.1560 |
| CAS | 8434 | *Lithobates sylvaticus* | Female | 1945 | -147.6082 | 64.9626 |
| CAS | 9300 | *Lithobates sylvaticus* | Female | 1947 | -149.7370 | 61.2894 |
| CAS | 9988 | *Lithobates sylvaticus* | Male | 1950 | -77.6564 | 37.9382 |
| CAS | 98982 | *Lithobates sylvaticus* | Female | 1957 | -149.9064 | 61.1886 |
| CAS | 98983 | *Lithobates sylvaticus* | Female | 1957 | -149.9064 | 61.1886 |
| CAS | 135237 | *Lithobates sylvaticus* | Male | 1972 | -136.0663 | 59.4197 |
| CAS | 135239 | *Lithobates sylvaticus* | Female | 1972 | -136.0663 | 59.4197 |
| CAS | 135242 | *Lithobates sylvaticus* | Male | 1972 | -136.0663 | 59.4197 |
| CAS | 173187 | *Lithobates sylvaticus* | Female | 1989 | -144.6721 | 65.5914 |
| CAS | 195071 | *Lithobates sylvaticus* | Male | 1962 | -73.3832 | 41.1818 |
| CAS | SU-2597 | *Lithobates sylvaticus* | Male | 1935 | -77.0170 | 39.0056 |
| CMNH | 1943 | *Lithobates sylvaticus* | Female | 1908 | -76.7989 | 40.8853 |
| CMNH | 1946 | *Lithobates sylvaticus* | Male | 1906 | -80.1263 | 42.1509 |
| CMNH | 4029 | *Lithobates sylvaticus* | Male | 1926 | -80.3799 | 41.5536 |
| CMNH | 4030 | *Lithobates sylvaticus* | Male | 1926 | -80.3799 | 41.5536 |
| CMNH | 4970 | *Lithobates sylvaticus* | Male | 1931 | -79.8503 | 40.5289 |
| CMNH | 4971 | *Lithobates sylvaticus* | Female | 1931 | -79.8503 | 40.5289 |
| CMNH | 5094 | *Lithobates sylvaticus* | Male | 1931 | -79.4922 | 39.8733 |
| CMNH | 5095 | *Lithobates sylvaticus* | Male | 1931 | -79.4922 | 39.8733 |
| CMNH | 5535 | *Lithobates sylvaticus* | Female | 1931 | -80.5004 | 41.2336 |
| CMNH | 5799 | *Lithobates sylvaticus* | Male | 1932 | -79.9203 | 40.5405 |
| CMNH | 5800 | *Lithobates sylvaticus* | Female | 1932 | -79.9203 | 40.5405 |
| CMNH | 5938 | *Lithobates sylvaticus* | Male | 1932 | -80.4227 | 41.6554 |
| CMNH | 5943 | *Lithobates sylvaticus* | Male | 1932 | -79.9203 | 40.5405 |
| CMNH | 6144 | *Lithobates sylvaticus* | Female | 1932 | -74.5625 | 40.8742 |
| CMNH | 6149 | *Lithobates sylvaticus* | Male | 1932 | -80.2438 | 39.4339 |
| CMNH | 7520 | *Lithobates sylvaticus* | Male | 1933 | -79.9469 | 40.5052 |
| CMNH | 7601 | *Lithobates sylvaticus* | Female | 1934 | -79.0941 | 40.5878 |
| CMNH | 7629 | *Lithobates sylvaticus* | Male | 1934 | -79.1266 | 40.5977 |
| CMNH | 8011 | *Lithobates sylvaticus* | Female | 1934 | -79.1560 | 40.6236 |
| CMNH | 8058 | *Lithobates sylvaticus* | Male | 1934 | -79.5563 | 41.9910 |
| CMNH | 8079 | *Lithobates sylvaticus* | Male | 1934 | -79.1976 | 40.4687 |
| CMNH | 8080 | *Lithobates sylvaticus* | Male | 1934 | -79.1976 | 40.4687 |
| CMNH | 8081 | *Lithobates sylvaticus* | Male | 1934 | -79.1976 | 40.4687 |
| CMNH | 8091 | *Lithobates sylvaticus* | Female | 1934 | -79.1976 | 40.4687 |
| CMNH | 8100 | *Lithobates sylvaticus* | Female | 1934 | -76.3639 | 39.8882 |
| CMNH | 9729 | *Lithobates sylvaticus* | Male | 1935 | -76.8867 | 40.3128 |
| CMNH | 9831 | *Lithobates sylvaticus* | Male | 1935 | -80.2594 | 41.3426 |
| CMNH | 9964 | *Lithobates sylvaticus* | Female | 1935 | -79.1201 | 40.3014 |
| CMNH | 10010 | *Lithobates sylvaticus* | Male | 1935 | -76.8526 | 40.3987 |
| CMNH | 10011 | *Lithobates sylvaticus* | Male | 1935 | -76.8526 | 40.3987 |
| CMNH | 10036 | *Lithobates sylvaticus* | Male | 1935 | -76.3639 | 39.8882 |
| CMNH | 10037 | *Lithobates sylvaticus* | Male | 1935 | -76.3639 | 39.8882 |
| CMNH | 10147 | *Lithobates sylvaticus* | Female | 1935 | -79.9276 | 38.6164 |
| CMNH | 10148 | *Lithobates sylvaticus* | Female | 1935 | -79.9276 | 38.6164 |
| CMNH | 10195 | *Lithobates sylvaticus* | Female | 1935 | -79.8740 | 38.6124 |
| CMNH | 10388 | *Lithobates sylvaticus* | Female | 1907 | -80.0332 | 40.5176 |
| CMNH | 10584 | *Lithobates sylvaticus* | Female | 1935 | -78.9086 | 40.0670 |
| CMNH | 10778 | *Lithobates sylvaticus* | Male | 1933 | -71.2097 | 42.3369 |
| CMNH | 10780 | *Lithobates sylvaticus* | Male | 1933 | -71.2097 | 42.3369 |
| CMNH | 10783 | *Lithobates sylvaticus* | Female | 1934 | -70.6678 | 41.9583 |
| CMNH | 10785 | *Lithobates sylvaticus* | Male | 1934 | -70.6678 | 41.9583 |
| CMNH | 11074 | *Lithobates sylvaticus* | Male | 1901 | -79.8900 | 40.5200 |
| CMNH | 11075 | *Lithobates sylvaticus* | Male | 1901 | -79.8900 | 40.5200 |
| CMNH | 11076 | *Lithobates sylvaticus* | Male | 1901 | -79.8900 | 40.5200 |
| CMNH | 11077 | *Lithobates sylvaticus* | Male | 1901 | -79.8900 | 40.5200 |
| CMNH | 11078 | *Lithobates sylvaticus* | Male | 1901 | -79.8900 | 40.5200 |
| CMNH | 11079 | *Lithobates sylvaticus* | Male | 1901 | -79.8900 | 40.5200 |
| CMNH | 11080 | *Lithobates sylvaticus* | Male | 1901 | -79.8900 | 40.5200 |
| CMNH | 11081 | *Lithobates sylvaticus* | Male | 1901 | -79.8900 | 40.5200 |
| CMNH | 11082 | *Lithobates sylvaticus* | Male | 1901 | -79.8900 | 40.5200 |
| CMNH | 11083 | *Lithobates sylvaticus* | Male | 1901 | -79.8900 | 40.5200 |
| CMNH | 11272 | *Lithobates sylvaticus* | Female | 1936 | -80.2984 | 38.8860 |
| CMNH | 11407 | *Lithobates sylvaticus* | Female | 1901 | -79.8910 | 40.5178 |
| CMNH | 11408 | *Lithobates sylvaticus* | Female | 1901 | -79.8910 | 40.5178 |
| CMNH | 11409 | *Lithobates sylvaticus* | Male | 1901 | -79.8910 | 40.5178 |
| CMNH | 11410 | *Lithobates sylvaticus* | Male | 1901 | -79.8910 | 40.5178 |
| CMNH | 11411 | *Lithobates sylvaticus* | Male | 1901 | -79.8910 | 40.5178 |
| CMNH | 11412 | *Lithobates sylvaticus* | Male | 1901 | -79.8910 | 40.5178 |
| CMNH | 11413 | *Lithobates sylvaticus* | Male | 1901 | -79.8910 | 40.5178 |
| CMNH | 11414 | *Lithobates sylvaticus* | Male | 1901 | -79.8910 | 40.5178 |
| CMNH | 11722 | *Lithobates sylvaticus* | Female | 1936 | -79.8770 | 38.6243 |
| CMNH | 11929 | *Lithobates sylvaticus* | Male | 1937 | -77.5607 | 39.8452 |
| CMNH | 12084 | *Lithobates sylvaticus* | Female | 1937 | -79.4204 | 38.9744 |
| CMNH | 12693 | *Lithobates sylvaticus* | Male | 1936 | -78.3227 | 40.4439 |
| CMNH | 12863 | *Lithobates sylvaticus* | Female | 1936 | -79.8140 | 39.0136 |
| CMNH | 12894 | *Lithobates sylvaticus* | Female | 1936 | -79.8440 | 38.8677 |
| CMNH | 13139 | *Lithobates sylvaticus* | Male | 1938 | -75.5907 | 39.8722 |
| CMNH | 13841 | *Lithobates sylvaticus* | Male | 1938 | -79.0096 | 39.4598 |
| CMNH | 13842 | *Lithobates sylvaticus* | Male | 1938 | -79.0096 | 39.4598 |
| CMNH | 13843 | *Lithobates sylvaticus* | Male | 1938 | -79.0096 | 39.4598 |
| CMNH | 13844 | *Lithobates sylvaticus* | Female | 1938 | -79.0096 | 39.4598 |
| CMNH | 13845 | *Lithobates sylvaticus* | Female | 1938 | -79.0096 | 39.4598 |
| CMNH | 13851 | *Lithobates sylvaticus* | Female | 1938 | -79.0096 | 39.4598 |
| CMNH | 13852 | *Lithobates sylvaticus* | Female | 1938 | -79.0096 | 39.4598 |
| CMNH | 13853 | *Lithobates sylvaticus* | Male | 1938 | -79.0096 | 39.4598 |
| CMNH | 14078 | *Lithobates sylvaticus* | Female | 1938 | -80.3027 | 39.5206 |
| CMNH | 15694 | *Lithobates sylvaticus* | Male | 1936 | -79.8140 | 39.0136 |
| CMNH | 15714 | *Lithobates sylvaticus* | Female | 1936 | -79.8618 | 39.0324 |
| CMNH | 15732 | *Lithobates sylvaticus* | Female | 1936 | -79.7054 | 38.8590 |
| CMNH | 15746 | *Lithobates sylvaticus* | Female | 1937 | -79.7177 | 39.0826 |
| CMNH | 16861 | *Lithobates sylvaticus* | Male | 1938 | -78.3705 | 40.0171 |
| CMNH | 16862 | *Lithobates sylvaticus* | Male | 1938 | -78.3705 | 40.0171 |
| CMNH | 16865 | *Lithobates sylvaticus* | Male | 1938 | -78.3705 | 40.0171 |
| CMNH | 17153 | *Lithobates sylvaticus* | Female | 1939 | -79.8367 | 40.5403 |
| CMNH | 19104 | *Lithobates sylvaticus* | Male | 1940 | -80.1273 | 39.8989 |
| CMNH | 19273 | *Lithobates sylvaticus* | Male | 1940 | -79.7183 | 41.0200 |
| CMNH | 19613 | *Lithobates sylvaticus* | Male | 1940 | -79.8314 | 38.5548 |
| CMNH | 19955 | *Lithobates sylvaticus* | Male | 1937 | -79.3984 | 40.2927 |
| CMNH | 20343 | *Lithobates sylvaticus* | Female | 1940 | -75.2064 | 40.6676 |
| CMNH | 20344 | *Lithobates sylvaticus* | Female | 1940 | -75.2064 | 40.6676 |
| CMNH | 20553 | *Lithobates sylvaticus* | Female | 1941 | -95.2018 | 47.1975 |
| CMNH | 20883 | *Lithobates sylvaticus* | Male | 1941 | -79.8699 | 39.6282 |
| CMNH | 20884 | *Lithobates sylvaticus* | Male | 1941 | -79.8699 | 39.6282 |
| CMNH | 20885 | *Lithobates sylvaticus* | Male | 1941 | -79.8699 | 39.6282 |
| CMNH | 20886 | *Lithobates sylvaticus* | Female | 1941 | -79.8699 | 39.6282 |
| CMNH | 20887 | *Lithobates sylvaticus* | Female | 1941 | -79.8699 | 39.6282 |
| CMNH | 21164 | *Lithobates sylvaticus* | Female | 1941 | -72.4469 | 44.3036 |
| CMNH | 21219 | *Lithobates sylvaticus* | Male | 1940 | -80.2658 | 38.1104 |
| CMNH | 21220 | *Lithobates sylvaticus* | Male | 1939 | -80.2658 | 38.1104 |
| CMNH | 21557 | *Lithobates sylvaticus* | Female | 1942 | -80.6208 | 51.2505 |
| CMNH | 21558 | *Lithobates sylvaticus* | Female | 1942 | -80.6208 | 51.2505 |
| CMNH | 21599 | *Lithobates sylvaticus* | Male | 1942 | -72.4773 | 44.2781 |
| CMNH | 21604 | *Lithobates sylvaticus* | Female | 1942 | -72.4469 | 44.3036 |
| CMNH | 21605 | *Lithobates sylvaticus* | Female | 1942 | -72.4469 | 44.3036 |
| CMNH | 21918 | *Lithobates sylvaticus* | Female | 1940 | -74.8932 | 41.0340 |
| CMNH | 22240 | *Lithobates sylvaticus* | Male | 1942 | -75.2452 | 41.0939 |
| CMNH | 22243 | *Lithobates sylvaticus* | Male | 1942 | -75.2452 | 41.0939 |
| CMNH | 22288 | *Lithobates sylvaticus* | Female | 1939 | -75.3590 | 41.0954 |
| CMNH | 22347 | *Lithobates sylvaticus* | Male | 1941 | -75.3342 | 41.1487 |
| CMNH | 22598 | *Lithobates sylvaticus* | Male | 1943 | -79.2281 | 41.8403 |
| CMNH | 24855 | *Lithobates sylvaticus* | Female | 1945 | -77.7153 | 41.6289 |
| CMNH | 24856 | *Lithobates sylvaticus* | Female | 1945 | -77.7153 | 41.6289 |
| CMNH | 24890 | *Lithobates sylvaticus* | Male | 1945 | -77.7153 | 41.6289 |
| CMNH | 25466 | *Lithobates sylvaticus* | Male | 1945 | -77.7153 | 41.6289 |
| CMNH | 25946 | *Lithobates sylvaticus* | Male | 1946 | -79.8367 | 40.5403 |
| CMNH | 25955 | *Lithobates sylvaticus* | Male | 1946 | -80.3635 | 40.4992 |
| CMNH | 26318 | *Lithobates sylvaticus* | Female | 1946 | -77.9058 | 40.0333 |
| CMNH | 26326 | *Lithobates sylvaticus* | Female | 1946 | -75.2064 | 40.6676 |
| CMNH | 26327 | *Lithobates sylvaticus* | Male | 1946 | -75.2064 | 40.6676 |
| CMNH | 26461 | *Lithobates sylvaticus* | Male | 1939 | -76.0154 | 39.7399 |
| CMNH | 26648 | *Lithobates sylvaticus* | Female | 1946 | -79.0827 | 39.0105 |
| CMNH | 26662 | *Lithobates sylvaticus* | Male | 1946 | -79.7732 | 38.5383 |
| CMNH | 27009 | *Lithobates sylvaticus* | Male | 1947 | -79.8751 | 38.6119 |
| CMNH | 27975 | *Lithobates sylvaticus* | Male | 1941 | -75.3062 | 41.3362 |
| CMNH | 28516 | *Lithobates sylvaticus* | Male | 1948 | -79.7164 | 38.5768 |
| CMNH | 29059 | *Lithobates sylvaticus* | Female | 1949 | -77.2005 | 40.8646 |
| CMNH | 29824 | *Lithobates sylvaticus* | Female | 1951 | -77.3603 | 40.0338 |
| CMNH | 30485 | *Lithobates sylvaticus* | Female | 1952 | -80.1092 | 40.8630 |
| CMNH | 30880 | *Lithobates sylvaticus* | Male | 1952 | -74.8492 | 39.8506 |
| CMNH | 30881 | *Lithobates sylvaticus* | Male | 1952 | -74.8492 | 39.8506 |
| CMNH | 30897 | *Lithobates sylvaticus* | Male | 1952 | -75.1574 | 39.6053 |
| CMNH | 31462 | *Lithobates sylvaticus* | Female | 1953 | -80.1863 | 40.9706 |
| CMNH | 31599 | *Lithobates sylvaticus* | Male | 1947 | -75.0327 | 40.3238 |
| CMNH | 31600 | *Lithobates sylvaticus* | Female | 1947 | -75.0327 | 40.3238 |
| CMNH | 31601 | *Lithobates sylvaticus* | Male | 1947 | -75.0327 | 40.3238 |
| CMNH | 31746 | *Lithobates sylvaticus* | Male | 1942 | -75.4267 | 40.3582 |
| CMNH | 31747 | *Lithobates sylvaticus* | Male | 1941 | -75.4292 | 40.3669 |
| CMNH | 32065 | *Lithobates sylvaticus* | Male | 1953 | -78.0664 | 40.7900 |
| CMNH | 32648 | *Lithobates sylvaticus* | Female | 1953 | -77.7152 | 41.5397 |
| CMNH | 34383 | *Lithobates sylvaticus* | Male | 1955 | -80.2049 | 40.9923 |
| CMNH | 34499 | *Lithobates sylvaticus* | Male | 1954 | -79.4467 | 39.0497 |
| CMNH | 34717 | *Lithobates sylvaticus* | Male | 1955 | -76.3397 | 40.2467 |
| CMNH | 35110 | *Lithobates sylvaticus* | Male | 1955 | -77.4608 | 41.4023 |
| CMNH | 35279 | *Lithobates sylvaticus* | Male | 1957 | -79.2712 | 40.1482 |
| CMNH | 35280 | *Lithobates sylvaticus* | Male | 1957 | -79.2712 | 40.1482 |
| CMNH | 36993 | *Lithobates sylvaticus* | Female | 1958 | -74.9381 | 40.2187 |
| CMNH | 36994 | *Lithobates sylvaticus* | Male | 1958 | -74.9381 | 40.2187 |
| CMNH | 36996 | *Lithobates sylvaticus* | Female | 1958 | -74.9381 | 40.2187 |
| CMNH | 38994 | *Lithobates sylvaticus* | Male | 1964 | -80.4244 | 40.5014 |
| CMNH | 52373 | *Lithobates sylvaticus* | Female | 1968 | -77.5262 | 41.8283 |
| CMNH | 68713 | *Lithobates sylvaticus* | Female | 1978 | -75.6762 | 41.3287 |
| CMNH | 69552 | *Lithobates sylvaticus* | Female | 1978 | -71.3623 | 43.5475 |
| CMNH | 69567 | *Lithobates sylvaticus* | Male | 1978 | -71.3917 | 45.0516 |
| CMNH | 69610 | *Lithobates sylvaticus* | Male | 1978 | -71.4050 | 43.1078 |
| CMNH | 69611 | *Lithobates sylvaticus* | Female | 1978 | -71.4050 | 43.1078 |
| CMNH | 83028 | *Lithobates sylvaticus* | Female | 1981 | -79.2860 | 41.2373 |
| CMNH | 83029 | *Lithobates sylvaticus* | Male | 1981 | -79.2860 | 41.2373 |
| CMNH | 86688 | *Lithobates sylvaticus* | Female | 1948 | -76.8689 | 41.3650 |
| CMNH | 86689 | *Lithobates sylvaticus* | Female | 1949 | -76.6037 | 41.4594 |
| CMNH | 86692 | *Lithobates sylvaticus* | Female | 1968 | -76.8261 | 41.4219 |
| CMNH | 109031 | *Lithobates sylvaticus* | Female | 1984 | -78.8829 | 41.9763 |
| CMNH | 109034 | *Lithobates sylvaticus* | Male | 1984 | -78.8793 | 41.8846 |
| CMNH | 111664 | *Lithobates sylvaticus* | Male | 1949 | -77.8332 | 40.6394 |
| CMNH | 111665 | *Lithobates sylvaticus* | Female | 1960 | -78.0750 | 40.9399 |
| CMNH | 111666 | *Lithobates sylvaticus* | Female | 1948 | -77.2098 | 41.0119 |
| CMNH | 111668 | *Lithobates sylvaticus* | Female | 1949 | -77.9642 | 40.7083 |
| CMNH | 113274 | *Lithobates sylvaticus* | Male | 1984 | -79.2712 | 40.1482 |
| CMNH | 113275 | *Lithobates sylvaticus* | Male | 1984 | -79.2712 | 40.1482 |
| CMNH | 113276 | *Lithobates sylvaticus* | Male | 1984 | -79.2712 | 40.1482 |
| CMNH | 113277 | *Lithobates sylvaticus* | Male | 1984 | -79.2712 | 40.1482 |
| CMNH | 113278 | *Lithobates sylvaticus* | Male | 1984 | -79.2712 | 40.1482 |
| CMNH | 113279 | *Lithobates sylvaticus* | Male | 1984 | -79.2712 | 40.1482 |
| CMNH | 115331 | *Lithobates sylvaticus* | Male | 1984 | -78.8944 | 41.8933 |
| CMNH | 115332 | *Lithobates sylvaticus* | Male | 1984 | -78.7096 | 41.8138 |
| CMNH | 116257 | *Lithobates sylvaticus* | Male | 1982 | -76.9276 | 41.2145 |
| CMNH | 116258 | *Lithobates sylvaticus* | Male | 1982 | -76.9276 | 41.2145 |
| CMNH | 116259 | *Lithobates sylvaticus* | Male | 1982 | -76.9276 | 41.2145 |
| CMNH | 116260 | *Lithobates sylvaticus* | Male | 1982 | -76.9276 | 41.2145 |
| CMNH | 116261 | *Lithobates sylvaticus* | Male | 1982 | -76.9276 | 41.2145 |
| CMNH | 116262 | *Lithobates sylvaticus* | Male | 1982 | -76.9276 | 41.2145 |
| CMNH | 116263 | *Lithobates sylvaticus* | Male | 1982 | -76.9276 | 41.2145 |
| CMNH | 117300 | *Lithobates sylvaticus* | Male | 1985 | -80.9171 | 37.7545 |
| CMNH | 117301 | *Lithobates sylvaticus* | Male | 1985 | -80.9171 | 37.7545 |
| CMNH | 117317 | *Lithobates sylvaticus* | Female | 1985 | -81.0966 | 37.8761 |
| CMNH | 118700 | *Lithobates sylvaticus* | Male | 1987 | -77.3985 | 39.9250 |
| CMNH | 118701 | *Lithobates sylvaticus* | Female | 1987 | -77.3441 | 40.0103 |
| CMNH | 119605 | *Lithobates sylvaticus* | Male | 1983 | -80.2271 | 37.2965 |
| CMNH | 120129 | *Lithobates sylvaticus* | Male | 1988 | -78.9900 | 37.9700 |
| CMNH | 120130 | *Lithobates sylvaticus* | Female | 1988 | -78.9900 | 37.9700 |
| CMNH | 120136 | *Lithobates sylvaticus* | Male | 1988 | -78.9900 | 37.9700 |
| CMNH | 120137 | *Lithobates sylvaticus* | Male | 1988 | -78.9900 | 37.9700 |
| CMNH | 120138 | *Lithobates sylvaticus* | Male | 1988 | -78.9900 | 37.9700 |
| CMNH | 120160 | *Lithobates sylvaticus* | Female | 1988 | -79.2675 | 38.4380 |
| CMNH | 120246 | *Lithobates sylvaticus* | Male | 1987 | -79.0689 | 38.4443 |
| CMNH | 122500 | *Lithobates sylvaticus* | Female | 1989 | -77.5000 | 39.9691 |
| CMNH | 126784 | *Lithobates sylvaticus* | Male | 1986 | -79.3200 | 37.6200 |
| CMNH | 126785 | *Lithobates sylvaticus* | Male | 1986 | -79.3200 | 37.6200 |
| CMNH | 126786 | *Lithobates sylvaticus* | Male | 1986 | -79.3200 | 37.6200 |
| CMNH | 126891 | *Lithobates sylvaticus* | Female | 1986 | -82.2600 | 37.2800 |
| CMNH | 127926 | *Lithobates sylvaticus* | Female | 1986 | -82.1677 | 37.3096 |
| CMNH | 128203 | *Lithobates sylvaticus* | Male | 1988 | -77.3800 | 38.5600 |
| CMNH | 129587 | *Lithobates sylvaticus* | Male | 1989 | -82.6238 | 36.7381 |
| CMNH | 140084 | *Lithobates sylvaticus* | Male | 1948 | -75.0367 | 40.0628 |
| CMNH | 140085 | *Lithobates sylvaticus* | Male | 1948 | -75.0367 | 40.0628 |
| CMNH | 140089 | *Lithobates sylvaticus* | Male | 1955 | -75.7304 | 40.1786 |
| CMNH | 140090 | *Lithobates sylvaticus* | Female | 1955 | -75.7304 | 40.1786 |
| CMNH | 143751 | *Lithobates sylvaticus* | Male | 1989 | -77.3463 | 40.0089 |
| CMNH | 143976 | *Lithobates sylvaticus* | Female | 1989 | -78.7610 | 41.6123 |
| CMNH | 144158 | *Lithobates sylvaticus* | Female | 1989 | -79.1894 | 41.6781 |
| CMNH | 144159 | *Lithobates sylvaticus* | Male | 1989 | -79.1894 | 41.6781 |
| CMNH | 144226 | *Lithobates sylvaticus* | Female | 1990 | -79.2710 | 40.7600 |
| CMNH | 144604 | *Lithobates sylvaticus* | Female | 1990 | -77.2096 | 40.9918 |
| CMNH | 144612 | *Lithobates sylvaticus* | Female | 1990 | -77.1282 | 40.9868 |
| CMNH | 147276 | *Lithobates sylvaticus* | Female | 1996 | -79.0944 | 41.5170 |
| CMNH | 147700 | *Lithobates sylvaticus* | Female | 1997 | -76.0116 | 40.6390 |
| CMNH | 148463 | *Lithobates sylvaticus* | Male | 1982 | -79.2587 | 40.1635 |
| CMNH | 149229 | *Lithobates sylvaticus* | Male | 1982 | -79.2700 | 40.1511 |
| CMNH | 149289 | *Lithobates sylvaticus* | Male | 1982 | -79.2814 | 40.1495 |
| CMNH | 149329 | *Lithobates sylvaticus* | Male | 1982 | -79.2838 | 40.1340 |
| CMNH | 149470 | *Lithobates sylvaticus* | Male | 1983 | -79.2632 | 40.1626 |
| CMNH | 152119 | *Lithobates sylvaticus* | Male | 1989 | -78.1760 | 37.7390 |
| CMNH | 152198 | *Lithobates sylvaticus* | Male | 1989 | -78.2130 | 37.6920 |
| CMNH | 152199 | *Lithobates sylvaticus* | Male | 1989 | -78.2130 | 37.6920 |
| CMNH | 152200 | *Lithobates sylvaticus* | Male | 1989 | -78.2130 | 37.6920 |
| CMNH | 152202 | *Lithobates sylvaticus* | Male | 1989 | -78.2130 | 37.6920 |
| CMNH | 152204 | *Lithobates sylvaticus* | Male | 1989 | -78.2130 | 37.6920 |
| CMNH | 152205 | *Lithobates sylvaticus* | Male | 1989 | -78.2130 | 37.6920 |
| CMNH | 152312 | *Lithobates sylvaticus* | Female | 1989 | -78.1760 | 37.7390 |
| CMNH | 152491 | *Lithobates sylvaticus* | Male | 1990 | -78.1760 | 37.7390 |
| CMNH | 152537 | *Lithobates sylvaticus* | Male | 1990 | -78.2130 | 37.6920 |
| CMNH | 152550 | *Lithobates sylvaticus* | Male | 1990 | -78.2130 | 37.6920 |
| CMNH | 152559 | *Lithobates sylvaticus* | Male | 1990 | -78.2130 | 37.6920 |
| CMNH | 152580 | *Lithobates sylvaticus* | Male | 1990 | -78.1760 | 37.7390 |
| CMNH | 152581 | *Lithobates sylvaticus* | Male | 1990 | -78.2130 | 37.6920 |
| CMNH | 152601 | *Lithobates sylvaticus* | Male | 1990 | -78.2130 | 37.6920 |
| CMNH | 152736 | *Lithobates sylvaticus* | Male | 1990 | -78.2130 | 37.6920 |
| CMNH | 152929 | *Lithobates sylvaticus* | Female | 1990 | -78.1634 | 37.7021 |
| CMNH | 152968 | *Lithobates sylvaticus* | Male | 1990 | -78.2130 | 37.6920 |
| CMNH | 152969 | *Lithobates sylvaticus* | Female | 1990 | -78.2130 | 37.6920 |
| CMNH | 153005 | *Lithobates sylvaticus* | Male | 1990 | -78.1790 | 37.7320 |
| CMNH | 153043 | *Lithobates sylvaticus* | Male | 1990 | -78.2130 | 37.6920 |
| CMNH | 153044 | *Lithobates sylvaticus* | Male | 1990 | -78.2130 | 37.6920 |
| CMNH | 153045 | *Lithobates sylvaticus* | Female | 1990 | -78.2130 | 37.6920 |
| CMNH | 153046 | *Lithobates sylvaticus* | Female | 1990 | -78.2130 | 37.6920 |
| CMNH | 153068 | *Lithobates sylvaticus* | Male | 1990 | -78.2130 | 37.6920 |
| CMNH | 155396 | *Lithobates sylvaticus* | Male | 1997 | -77.3886 | 38.1516 |
| CMNH | 155397 | *Lithobates sylvaticus* | Male | 1997 | -77.3886 | 38.1516 |
| CMNH | 20553-C | *Lithobates sylvaticus* | Female | 1941 | -95.2018 | 47.1975 |
| CMNH | 20553-D | *Lithobates sylvaticus* | Female | 1941 | -95.2018 | 47.1975 |
| CMNH | 21164-B | *Lithobates sylvaticus* | Female | 1941 | -72.4469 | 44.3036 |
| CMNH | 21164-C | *Lithobates sylvaticus* | Male | 1941 | -72.4469 | 44.3036 |
| CMNH | 21164-D | *Lithobates sylvaticus* | Male | 1941 | -72.4469 | 44.3036 |
| CMNH | 21164-D | *Lithobates sylvaticus* | Male | 1941 | -72.4469 | 44.3036 |
| CMNH | 21164-E | *Lithobates sylvaticus* | Male | 1941 | -72.4469 | 44.3036 |
| CMNH | 21164-F | *Lithobates sylvaticus* | Female | 1941 | -72.4469 | 44.3036 |
| CMNH | 21164-G | *Lithobates sylvaticus* | Male | 1941 | -72.4469 | 44.3036 |
| CMNH | 21164-H | *Lithobates sylvaticus* | Male | 1941 | -72.4469 | 44.3036 |
| CMNH | 21164-I | *Lithobates sylvaticus* | Male | 1941 | -72.4469 | 44.3036 |
| CMNH | 21164-K | *Lithobates sylvaticus* | Male | 1941 | -72.4469 | 44.3036 |
| CMNH | 21599-AA | *Lithobates sylvaticus* | Female | 1942 | -72.4773 | 44.2781 |
| CMNH | 21599-AB | *Lithobates sylvaticus* | Male | 1942 | -72.4773 | 44.2781 |
| CMNH | 21599-AC | *Lithobates sylvaticus* | Male | 1942 | -72.4773 | 44.2781 |
| CMNH | 21599-AD | *Lithobates sylvaticus* | Male | 1942 | -72.4773 | 44.2781 |
| CMNH | 21599-AE | *Lithobates sylvaticus* | Male | 1942 | -72.4773 | 44.2781 |
| CMNH | 21599-AF | *Lithobates sylvaticus* | Male | 1942 | -72.4773 | 44.2781 |
| CMNH | 21599-AG | *Lithobates sylvaticus* | Male | 1942 | -72.4773 | 44.2781 |
| CMNH | 21599-AH | *Lithobates sylvaticus* | Male | 1942 | -72.4773 | 44.2781 |
| CMNH | 21599-AI | *Lithobates sylvaticus* | Male | 1942 | -72.4773 | 44.2781 |
| CMNH | 21599-AJ | *Lithobates sylvaticus* | Male | 1942 | -72.4773 | 44.2781 |
| CMNH | 21599-AK | *Lithobates sylvaticus* | Male | 1942 | -72.4773 | 44.2781 |
| CMNH | 21599-AL | *Lithobates sylvaticus* | Male | 1942 | -72.4773 | 44.2781 |
| CMNH | 21599-AM | *Lithobates sylvaticus* | Male | 1942 | -72.4773 | 44.2781 |
| CMNH | 21599-AN | *Lithobates sylvaticus* | Female | 1942 | -72.4773 | 44.2781 |
| CMNH | 21599-AO | *Lithobates sylvaticus* | Male | 1942 | -72.4773 | 44.2781 |
| CMNH | 21599-AP | *Lithobates sylvaticus* | Male | 1942 | -72.4773 | 44.2781 |
| CMNH | 21599-AQ | *Lithobates sylvaticus* | Male | 1942 | -72.4773 | 44.2781 |
| CMNH | 21599-AR | *Lithobates sylvaticus* | Male | 1942 | -72.4773 | 44.2781 |
| CMNH | 21599-AS | *Lithobates sylvaticus* | Male | 1942 | -72.4773 | 44.2781 |
| CMNH | 21599-B | *Lithobates sylvaticus* | Male | 1942 | -72.4773 | 44.2781 |
| CMNH | 21599-BB | *Lithobates sylvaticus* | Male | 1942 | -72.4773 | 44.2781 |
| CMNH | 21599-C | *Lithobates sylvaticus* | Male | 1942 | -72.4773 | 44.2781 |
| CMNH | 21599-CC | *Lithobates sylvaticus* | Male | 1942 | -72.4773 | 44.2781 |
| CMNH | 21599-D | *Lithobates sylvaticus* | Female | 1942 | -72.4773 | 44.2781 |
| CMNH | 21599-DD | *Lithobates sylvaticus* | Male | 1942 | -72.4773 | 44.2781 |
| CMNH | 21599-E | *Lithobates sylvaticus* | Male | 1942 | -72.4773 | 44.2781 |
| CMNH | 21599-EE | *Lithobates sylvaticus* | Male | 1942 | -72.4773 | 44.2781 |
| CMNH | 21599-F | *Lithobates sylvaticus* | Male | 1942 | -72.4773 | 44.2781 |
| CMNH | 21599-FF | *Lithobates sylvaticus* | Female | 1942 | -72.4773 | 44.2781 |
| CMNH | 21599-G | *Lithobates sylvaticus* | Female | 1942 | -72.4773 | 44.2781 |
| CMNH | 21599-GG | *Lithobates sylvaticus* | Female | 1942 | -72.4773 | 44.2781 |
| CMNH | 21599-H | *Lithobates sylvaticus* | Male | 1942 | -72.4773 | 44.2781 |
| CMNH | 21599-HH | *Lithobates sylvaticus* | Male | 1942 | -72.4773 | 44.2781 |
| CMNH | 21599-I | *Lithobates sylvaticus* | Female | 1942 | -72.4773 | 44.2781 |
| CMNH | 21599-II | *Lithobates sylvaticus* | Male | 1942 | -72.4773 | 44.2781 |
| CMNH | 21599-J | *Lithobates sylvaticus* | Male | 1942 | -72.4773 | 44.2781 |
| CMNH | 21599-JJ | *Lithobates sylvaticus* | Male | 1942 | -72.4773 | 44.2781 |
| CMNH | 21599-K | *Lithobates sylvaticus* | Male | 1942 | -72.4773 | 44.2781 |
| CMNH | 21599-KK | *Lithobates sylvaticus* | Male | 1942 | -72.4773 | 44.2781 |
| CMNH | 21599-L | *Lithobates sylvaticus* | Male | 1942 | -72.4773 | 44.2781 |
| CMNH | 21599-LL | *Lithobates sylvaticus* | Male | 1942 | -72.4773 | 44.2781 |
| CMNH | 21599-M | *Lithobates sylvaticus* | Male | 1942 | -72.4773 | 44.2781 |
| CMNH | 21599-MM | *Lithobates sylvaticus* | Male | 1942 | -72.4773 | 44.2781 |
| CMNH | 21599-N | *Lithobates sylvaticus* | Male | 1942 | -72.4773 | 44.2781 |
| CMNH | 21599-NN | *Lithobates sylvaticus* | Male | 1942 | -72.4773 | 44.2781 |
| CMNH | 21599-O | *Lithobates sylvaticus* | Female | 1942 | -72.4773 | 44.2781 |
| CMNH | 21599-OO | *Lithobates sylvaticus* | Male | 1942 | -72.4773 | 44.2781 |
| CMNH | 21599-P | *Lithobates sylvaticus* | Male | 1942 | -72.4773 | 44.2781 |
| CMNH | 21599-PP | *Lithobates sylvaticus* | Male | 1942 | -72.4773 | 44.2781 |
| CMNH | 21599-Q | *Lithobates sylvaticus* | Male | 1942 | -72.4773 | 44.2781 |
| CMNH | 21599-QQ | *Lithobates sylvaticus* | Female | 1942 | -72.4773 | 44.2781 |
| CMNH | 21599-R | *Lithobates sylvaticus* | Female | 1942 | -72.4773 | 44.2781 |
| CMNH | 21599-RR | *Lithobates sylvaticus* | Male | 1942 | -72.4773 | 44.2781 |
| CMNH | 21599-S | *Lithobates sylvaticus* | Male | 1942 | -72.4773 | 44.2781 |
| CMNH | 21599-SS | *Lithobates sylvaticus* | Male | 1942 | -72.4773 | 44.2781 |
| CMNH | 21599-T | *Lithobates sylvaticus* | Male | 1942 | -72.4773 | 44.2781 |
| CMNH | 21599-TT | *Lithobates sylvaticus* | Male | 1942 | -72.4773 | 44.2781 |
| CMNH | 21599-U | *Lithobates sylvaticus* | Male | 1942 | -72.4773 | 44.2781 |
| CMNH | 21599-UU | *Lithobates sylvaticus* | Female | 1942 | -72.4773 | 44.2781 |
| CMNH | 21599-V | *Lithobates sylvaticus* | Female | 1942 | -72.4773 | 44.2781 |
| CMNH | 21599-VV | *Lithobates sylvaticus* | Male | 1942 | -72.4773 | 44.2781 |
| CMNH | 21599-W | *Lithobates sylvaticus* | Male | 1942 | -72.4773 | 44.2781 |
| CMNH | 21599-WW | *Lithobates sylvaticus* | Male | 1942 | -72.4773 | 44.2781 |
| CMNH | 21599-X | *Lithobates sylvaticus* | Female | 1942 | -72.4773 | 44.2781 |
| CMNH | 21599-XX | *Lithobates sylvaticus* | Male | 1942 | -72.4773 | 44.2781 |
| CMNH | 21599-Y | *Lithobates sylvaticus* | Male | 1942 | -72.4773 | 44.2781 |
| CMNH | 21599-YY | *Lithobates sylvaticus* | Female | 1942 | -72.4773 | 44.2781 |
| CMNH | 21599-Z | *Lithobates sylvaticus* | Male | 1942 | -72.4773 | 44.2781 |
| CMNH | 21599-ZZ | *Lithobates sylvaticus* | Male | 1942 | -72.4773 | 44.2781 |
| CMNH | 29671-BB | *Lithobates sylvaticus* | Male | 1950 | -75.5190 | 41.1016 |
| CMNH | 29671-C | *Lithobates sylvaticus* | Female | 1950 | -75.5190 | 41.1016 |
| CMNH | 29671-DD | *Lithobates sylvaticus* | Male | 1950 | -75.5190 | 41.1016 |
| CMNH | 29671-E | *Lithobates sylvaticus* | Male | 1950 | -75.5190 | 41.1016 |
| CMNH | 29671-EE | *Lithobates sylvaticus* | Male | 1950 | -75.5190 | 41.1016 |
| CMNH | 29671-F | *Lithobates sylvaticus* | Male | 1950 | -75.5190 | 41.1016 |
| CMNH | 29671-G | *Lithobates sylvaticus* | Male | 1950 | -75.5190 | 41.1016 |
| CMNH | 29671-H | *Lithobates sylvaticus* | Female | 1950 | -75.5190 | 41.1016 |
| CMNH | 29671-L | *Lithobates sylvaticus* | Male | 1950 | -75.5190 | 41.1016 |
| CMNH | 29671-N | *Lithobates sylvaticus* | Male | 1950 | -75.5190 | 41.1016 |
| CMNH | 29671-O | *Lithobates sylvaticus* | Male | 1950 | -75.5190 | 41.1016 |
| CMNH | 29671-P | *Lithobates sylvaticus* | Male | 1950 | -75.5190 | 41.1016 |
| CMNH | 29671-S | *Lithobates sylvaticus* | Male | 1950 | -75.5190 | 41.1016 |
| CMNH | 29671-T | *Lithobates sylvaticus* | Male | 1950 | -75.5190 | 41.1016 |
| CMNH | 29671-U | *Lithobates sylvaticus* | Male | 1950 | -75.5190 | 41.1016 |
| CMNH | 29671-V | *Lithobates sylvaticus* | Male | 1950 | -75.5190 | 41.1016 |
| CMNH | 29671-W | *Lithobates sylvaticus* | Male | 1950 | -75.5190 | 41.1016 |
| CMNH | 29671-Y | *Lithobates sylvaticus* | Male | 1950 | -75.5190 | 41.1016 |
| CMNH | 29671-Z | *Lithobates sylvaticus* | Male | 1950 | -75.5190 | 41.1016 |
| CMNH | 4172-A | *Lithobates sylvaticus* | Male | 1928 | -79.1282 | 40.1780 |
| CMNH | 4172-B | *Lithobates sylvaticus* | Female | 1928 | -79.1282 | 40.1780 |
| CMNH | 4172-C | *Lithobates sylvaticus* | Male | 1928 | -79.1282 | 40.1780 |
| CMNH | 4172-D | *Lithobates sylvaticus* | Male | 1928 | -79.1282 | 40.1780 |
| CMNH | 4172-E | *Lithobates sylvaticus* | Male | 1928 | -79.1282 | 40.1780 |
| CMNH | 5904-A | *Lithobates sylvaticus* | Male | 1932 | -79.9546 | 41.2338 |
| CUMV | 2969 | *Lithobates sylvaticus* | Male | 1936 | -76.3649 | 42.4503 |
| CUMV | 3259 | *Lithobates sylvaticus* | Female | 1931 | -80.3277 | 37.6472 |
| CUMV | 3711 | *Lithobates sylvaticus* | Female | 1937 | -74.1377 | 42.5162 |
| CUMV | 3843 | *Lithobates sylvaticus* | Female | 1939 | -72.2342 | 44.7190 |
| CUMV | 4001 | *Lithobates sylvaticus* | Male | 1940 | -94.1400 | 58.7465 |
| CUMV | 4343 | *Lithobates sylvaticus* | Male | 1942 | -76.4595 | 42.4596 |
| CUMV | 4349 | *Lithobates sylvaticus* | Male | 1942 | -76.3649 | 42.4503 |
| CUMV | 4361 | *Lithobates sylvaticus* | Male | 1942 | -76.3649 | 42.4503 |
| CUMV | 4601 | *Lithobates sylvaticus* | Female | 1934 | -74.5778 | 40.6647 |
| CUMV | 4624 | *Lithobates sylvaticus* | Female | 1935 | -74.5475 | 40.6853 |
| CUMV | 4629 | *Lithobates sylvaticus* | Male | 1935 | -74.5453 | 40.6197 |
| CUMV | 5305 | *Lithobates sylvaticus* | Male | 1946 | -77.1061 | 42.3749 |
| CUMV | 5306 | *Lithobates sylvaticus* | Male | 1946 | -76.7429 | 42.3122 |
| CUMV | 5308 | *Lithobates sylvaticus* | Male | 1946 | -76.8033 | 42.4666 |
| CUMV | 5311 | *Lithobates sylvaticus* | Male | 1946 | -76.8067 | 42.1567 |
| CUMV | 5312 | *Lithobates sylvaticus* | Male | 1946 | -76.8196 | 42.2648 |
| CUMV | 5458 | *Lithobates sylvaticus* | Female | 1947 | -74.2871 | 44.3535 |
| CUMV | 5864 | *Lithobates sylvaticus* | Male | 1949 | -76.3649 | 42.4503 |
| CUMV | 6860 | *Lithobates sylvaticus* | Female | 1950 | -76.5461 | 42.3960 |
| CUMV | 6969 | *Lithobates sylvaticus* | Male | 1950 | -76.4595 | 42.4596 |
| CUMV | 9579 | *Lithobates sylvaticus* | Male | 1974 | -76.3649 | 42.4503 |
| CUMV | 11943 | *Lithobates sylvaticus* | Female | 1977 | -102.3802 | 49.8139 |
| CUMV | 12400 | *Lithobates sylvaticus* | Female | 1948 | -78.4303 | 42.0783 |
| CUMV | 12963 | *Lithobates sylvaticus* | Male | 2000 | -76.3649 | 42.4503 |
| CUMV | 13403 | *Lithobates sylvaticus* | Female | 2000 | -76.3951 | 42.3826 |
| CUMV | 13404 | *Lithobates sylvaticus* | Female | 2000 | -76.3951 | 42.3826 |
| CUMV | 13405 | *Lithobates sylvaticus* | Female | 2000 | -76.3951 | 42.3826 |
| CUMV | 12541a | *Lithobates sylvaticus* | Male | 1948 | -76.4595 | 42.4596 |
| CUMV | 12541b | *Lithobates sylvaticus* | Male | 1948 | -76.4595 | 42.4596 |
| CUMV | 12541c | *Lithobates sylvaticus* | Male | 1948 | -76.4595 | 42.4596 |
| CUMV | 12541d | *Lithobates sylvaticus* | Male | 1948 | -76.4595 | 42.4596 |
| CUMV | 2379a | *Lithobates sylvaticus* | Male | 1931 | -76.3649 | 42.4503 |
| CUMV | 2379b | *Lithobates sylvaticus* | Male | 1931 | -76.3649 | 42.4503 |
| CUMV | 2431a | *Lithobates sylvaticus* | Male | 1930 | -76.3649 | 42.4503 |
| CUMV | 2431b | *Lithobates sylvaticus* | Male | 1930 | -76.3649 | 42.4503 |
| CUMV | 3991a | *Lithobates sylvaticus* | Female | 1940 | -65.8693 | 49.1611 |
| CUMV | 3991b | *Lithobates sylvaticus* | Female | 1940 | -65.8693 | 49.1611 |
| CUMV | 3991c | *Lithobates sylvaticus* | Male | 1940 | -65.8693 | 49.1611 |
| CUMV | 4298a | *Lithobates sylvaticus* | Female | 1942 | -74.9042 | 41.6292 |
| CUMV | 4298b | *Lithobates sylvaticus* | Male | 1942 | -74.9042 | 41.6292 |
| CUMV | 5693a | *Lithobates sylvaticus* | Female | 1955 | -76.5522 | 42.1987 |
| CUMV | 5693b | *Lithobates sylvaticus* | Female | 1955 | -76.5522 | 42.1987 |
| CUMV | 5818a | *Lithobates sylvaticus* | Male | 1948 | -76.3649 | 42.4503 |
| CUMV | 5818b | *Lithobates sylvaticus* | Male | 1948 | -76.3649 | 42.4503 |
| CUMV | 5818c | *Lithobates sylvaticus* | Male | 1948 | -76.3649 | 42.4503 |
| CUMV | 5818d | *Lithobates sylvaticus* | Male | 1948 | -76.3649 | 42.4503 |
| CUMV | 5818e | *Lithobates sylvaticus* | Male | 1948 | -76.3649 | 42.4503 |
| CUMV | 5843a | *Lithobates sylvaticus* | Male | 1948 | -76.3649 | 42.4503 |
| CUMV | 5843b | *Lithobates sylvaticus* | Male | 1948 | -76.3649 | 42.4503 |
| CUMV | 5850a | *Lithobates sylvaticus* | Female | 1948 | -76.3649 | 42.4503 |
| CUMV | 5850b | *Lithobates sylvaticus* | Male | 1948 | -76.3649 | 42.4503 |
| CUMV | 5850c | *Lithobates sylvaticus* | Male | 1948 | -76.3649 | 42.4503 |
| CUMV | 5850d | *Lithobates sylvaticus* | Male | 1948 | -76.3649 | 42.4503 |
| CUMV | 6978a | *Lithobates sylvaticus* | Female | 1958 | -73.7738 | 43.4954 |
| CUMV | 6978b | *Lithobates sylvaticus* | Female | 1958 | -73.7738 | 43.4954 |
| FMNH | 11977 | *Lithobates sylvaticus* | Female | 1931 | -87.9124 | 42.1681 |
| FMNH | 94540 | *Lithobates sylvaticus* | Female | 1930 | -77.5266 | 43.1760 |
| FMNH | 94546 | *Lithobates sylvaticus* | Female | 1930 | -77.5266 | 43.1760 |
| FMNH | 162068 | *Lithobates sylvaticus* | Male | 1946 | -87.9069 | 42.1530 |
| FMNH | 162069 | *Lithobates sylvaticus* | Male | 1946 | -87.9069 | 42.1530 |
| FMNH | 162175 | *Lithobates sylvaticus* | Male | 1947 | -87.9124 | 42.1681 |
| FMNH | 162178 | *Lithobates sylvaticus* | Male | 1947 | -87.9124 | 42.1681 |
| FMNH | 162179 | *Lithobates sylvaticus* | Male | 1947 | -87.9124 | 42.1681 |
| FMNH | 162180 | *Lithobates sylvaticus* | Female | 1947 | -87.9124 | 42.1681 |
| FMNH | 162181 | *Lithobates sylvaticus* | Female | 1947 | -87.9124 | 42.1681 |
| FMNH | 162182 | *Lithobates sylvaticus* | Male | 1947 | -87.9124 | 42.1681 |
| FMNH | 213007 | *Lithobates sylvaticus* | Female | 1980 | -87.5187 | 44.2022 |
| FMNH | 213010 | *Lithobates sylvaticus* | Female | 1980 | -87.5187 | 44.2022 |
| FMNH | 213011 | *Lithobates sylvaticus* | Female | 1980 | -87.5187 | 44.2022 |
| FMNH | 213026 | *Lithobates sylvaticus* | Female | 1980 | -88.2290 | 43.4858 |
| FMNH | 213029 | *Lithobates sylvaticus* | Female | 1980 | -88.2290 | 43.4858 |
| FMNH | 213031 | *Lithobates sylvaticus* | Female | 1980 | -88.2290 | 43.4858 |
| FMNH | 213032 | *Lithobates sylvaticus* | Female | 1980 | -88.2290 | 43.4858 |
| FMNH | 213036 | *Lithobates sylvaticus* | Male | 1980 | -88.2290 | 43.4858 |
| FMNH | 213037 | *Lithobates sylvaticus* | Male | 1980 | -88.2290 | 43.4858 |
| FMNH | 213038 | *Lithobates sylvaticus* | Male | 1980 | -88.2290 | 43.4858 |
| FMNH | 213039 | *Lithobates sylvaticus* | Male | 1980 | -88.2290 | 43.4858 |
| FMNH | 213040 | *Lithobates sylvaticus* | Male | 1980 | -88.2290 | 43.4858 |
| FMNH | 213041 | *Lithobates sylvaticus* | Male | 1980 | -88.2290 | 43.4858 |
| FMNH | 213042 | *Lithobates sylvaticus* | Male | 1980 | -88.2290 | 43.4858 |
| FMNH | 213043 | *Lithobates sylvaticus* | Male | 1980 | -95.0000 | 47.0000 |
| LACM | 1993 | *Lithobates sylvaticus* | Female | 1961 | -75.6897 | 37.6043 |
| LACM | 13896 | *Lithobates sylvaticus* | Female | 1957 | -150.1961 | 60.4527 |
| LACM | 13899 | *Lithobates sylvaticus* | Male | 1955 | -94.5217 | 47.0450 |
| LACM | 13905 | *Lithobates sylvaticus* | Female | 1955 | -95.2018 | 47.1975 |
| LACM | 13908 | *Lithobates sylvaticus* | Female | 1955 | -95.2018 | 47.1975 |
| LACM | 25954 | *Lithobates sylvaticus* | Female | 1960 | -83.1968 | 35.0526 |
| LACM | 36225 | *Lithobates sylvaticus* | Male | 1966 | -103.6667 | 61.2000 |
| LACM | 53119 | *Lithobates sylvaticus* | Male | 1951 | -151.5667 | 66.9000 |
| LACM | 60547 | *Lithobates sylvaticus* | Female | 1959 | -74.8269 | 41.0522 |
| LACM | 60548 | *Lithobates sylvaticus* | Female | 1964 | -72.0125 | 42.6406 |
| LACM | 65405 | *Lithobates sylvaticus* | Female | 1965 | -76.7338 | 39.2297 |
| LACM | 65406 | *Lithobates sylvaticus* | Male | 1965 | -76.7338 | 39.2297 |
| LACM | 65407 | *Lithobates sylvaticus* | Male | 1965 | -76.7338 | 39.2297 |
| LACM | 65408 | *Lithobates sylvaticus* | Male | 1965 | -76.7338 | 39.2297 |
| LACM | 65409 | *Lithobates sylvaticus* | Male | 1965 | -76.7338 | 39.2297 |
| LACM | 65410 | *Lithobates sylvaticus* | Male | 1965 | -76.7338 | 39.2297 |
| LACM | 65411 | *Lithobates sylvaticus* | Male | 1965 | -76.7338 | 39.2297 |
| LACM | 65412 | *Lithobates sylvaticus* | Male | 1965 | -76.7338 | 39.2297 |
| LACM | 65413 | *Lithobates sylvaticus* | Male | 1965 | -76.7338 | 39.2297 |
| LACM | 65414 | *Lithobates sylvaticus* | Male | 1965 | -76.7338 | 39.2297 |
| LACM | 65415 | *Lithobates sylvaticus* | Male | 1965 | -76.7338 | 39.2297 |
| LACM | 65416 | *Lithobates sylvaticus* | Male | 1965 | -76.7338 | 39.2297 |
| LACM | 65417 | *Lithobates sylvaticus* | Female | 1965 | -76.7338 | 39.2297 |
| LACM | 65418 | *Lithobates sylvaticus* | Female | 1965 | -76.7338 | 39.2297 |
| LACM | 65419 | *Lithobates sylvaticus* | Male | 1965 | -76.7338 | 39.2297 |
| LACM | 65420 | *Lithobates sylvaticus* | Female | 1965 | -76.7338 | 39.2297 |
| LACM | 65421 | *Lithobates sylvaticus* | Female | 1965 | -76.7338 | 39.2297 |
| LACM | 65422 | *Lithobates sylvaticus* | Female | 1965 | -76.7338 | 39.2297 |
| LACM | 65423 | *Lithobates sylvaticus* | Male | 1965 | -76.7338 | 39.2297 |
| LACM | 65424 | *Lithobates sylvaticus* | Male | 1965 | -76.7338 | 39.2297 |
| LACM | 65425 | *Lithobates sylvaticus* | Male | 1965 | -76.7338 | 39.2297 |
| LACM | 65426 | *Lithobates sylvaticus* | Male | 1965 | -76.7338 | 39.2297 |
| LACM | 65427 | *Lithobates sylvaticus* | Male | 1966 | -76.7504 | 39.2307 |
| LACM | 65428 | *Lithobates sylvaticus* | Male | 1966 | -76.7504 | 39.2307 |
| LACM | 65429 | *Lithobates sylvaticus* | Male | 1966 | -76.7504 | 39.2307 |
| LACM | 65430 | *Lithobates sylvaticus* | Male | 1966 | -76.7504 | 39.2307 |
| LACM | 65431 | *Lithobates sylvaticus* | Male | 1966 | -76.7504 | 39.2307 |
| LACM | 65432 | *Lithobates sylvaticus* | Male | 1966 | -76.7504 | 39.2307 |
| LACM | 65433 | *Lithobates sylvaticus* | Male | 1966 | -76.7504 | 39.2307 |
| LACM | 65434 | *Lithobates sylvaticus* | Male | 1966 | -76.7504 | 39.2307 |
| LACM | 65435 | *Lithobates sylvaticus* | Male | 1966 | -76.7504 | 39.2307 |
| LACM | 65438 | *Lithobates sylvaticus* | Female | 1965 | -76.7836 | 38.6398 |
| LACM | 65439 | *Lithobates sylvaticus* | Male | 1965 | -76.7836 | 38.6398 |
| LACM | 65441 | *Lithobates sylvaticus* | Male | 1965 | -76.9386 | 38.9859 |
| LACM | 65442 | *Lithobates sylvaticus* | Male | 1963 | -76.6939 | 38.9022 |
| LACM | 65444 | *Lithobates sylvaticus* | Female | 1965 | -76.7836 | 38.6398 |
| LACM | 65450 | *Lithobates sylvaticus* | Female | 1965 | -76.6467 | 38.9907 |
| LACM | 76527 | *Lithobates sylvaticus* | Male | 1970 | -116.9854 | 48.6235 |
| LACM | 76529 | *Lithobates sylvaticus* | Female | 1970 | -116.9854 | 48.6235 |
| LACM | 91500 | *Lithobates sylvaticus* | Female | 1968 | -150.0070 | 61.2690 |
| LACM | 106090 | *Lithobates sylvaticus* | Female | 1972 | -96.1727 | 47.6568 |
| LACM | 106091 | *Lithobates sylvaticus* | Male | 1972 | -96.1727 | 47.6568 |
| LACM | 106094 | *Lithobates sylvaticus* | Male | 1972 | -96.1727 | 47.6568 |
| LACM | 106095 | *Lithobates sylvaticus* | Male | 1972 | -96.1727 | 47.6568 |
| LACM | 106097 | *Lithobates sylvaticus* | Male | 1972 | -96.1727 | 47.6568 |
| LACM | 106099 | *Lithobates sylvaticus* | Male | 1972 | -96.1727 | 47.6568 |
| LACM | 106102 | *Lithobates sylvaticus* | Male | 1972 | -96.1727 | 47.6568 |
| LACM | 106103 | *Lithobates sylvaticus* | Male | 1972 | -96.1727 | 47.6568 |
| LACM | 106105 | *Lithobates sylvaticus* | Male | 1972 | -96.1727 | 47.6568 |
| LACM | 106106 | *Lithobates sylvaticus* | Male | 1972 | -96.1727 | 47.6568 |
| LACM | 106110 | *Lithobates sylvaticus* | Male | 1972 | -96.1727 | 47.6568 |
| LACM | 106111 | *Lithobates sylvaticus* | Male | 1972 | -96.1727 | 47.6568 |
| MCZ | 6020 | *Lithobates sylvaticus* | Female | 1908 | -83.1756 | 43.9658 |
| MCZ | 8183 | *Lithobates sylvaticus* | Female | 1920 | -111.4500 | 58.8685 |
| MCZ | 8184 | *Lithobates sylvaticus* | Female | 1920 | -111.0637 | 58.5485 |
| MCZ | 15038 | *Lithobates sylvaticus* | Female | 1924 | -113.5110 | 53.5407 |
| MCZ | 25779 | *Lithobates sylvaticus* | Male | 1944 | -144.7127 | 63.7681 |
| MCZ | 26823 | *Lithobates sylvaticus* | Male | 1950 | -77.7839 | 55.2671 |
| MCZ | 26826 | *Lithobates sylvaticus* | Female | 1950 | -76.2589 | 56.3211 |
| MCZ | 26827 | *Lithobates sylvaticus* | Female | 1950 | -76.2589 | 56.3211 |
| MCZ | 26828 | *Lithobates sylvaticus* | Female | 1950 | -76.2589 | 56.3211 |
| MCZ | 66713 | *Lithobates sylvaticus* | Male | 1967 | -66.1349 | 45.4727 |
| MCZ | 66714 | *Lithobates sylvaticus* | Male | 1967 | -66.1349 | 45.4727 |
| MCZ | 66715 | *Lithobates sylvaticus* | Male | 1967 | -66.1349 | 45.4727 |
| MCZ | 66716 | *Lithobates sylvaticus* | Female | 1967 | -66.1349 | 45.4727 |
| MCZ | 82942 | *Lithobates sylvaticus* | Female | 1972 | -72.9706 | 42.0781 |
| MCZ | 91289 | *Lithobates sylvaticus* | Female | 1976 | -73.2853 | 42.3564 |
| MCZ | 93525 | *Lithobates sylvaticus* | Female | 1977 | -71.8778 | 42.4486 |
| MCZ | 96620 | *Lithobates sylvaticus* | Female | 1978 | -72.7175 | 42.6171 |
| MCZ | 99972 | *Lithobates sylvaticus* | Male | 1980 | -94.8631 | 47.3356 |
| MCZ | 99973 | *Lithobates sylvaticus* | Male | 1980 | -94.8631 | 47.3356 |
| MCZ | 99974 | *Lithobates sylvaticus* | Male | 1980 | -94.8631 | 47.3356 |
| MCZ | 99975 | *Lithobates sylvaticus* | Male | 1980 | -94.8631 | 47.3356 |
| MCZ | 99983 | *Lithobates sylvaticus* | Male | 1980 | -95.1522 | 47.1394 |
| MCZ | 99987 | *Lithobates sylvaticus* | Male | 1980 | -95.1522 | 47.1394 |
| MCZ | 99990 | *Lithobates sylvaticus* | Male | 1980 | -95.1522 | 47.1394 |
| MCZ | 100297 | *Lithobates sylvaticus* | Male | 1981 | -71.7581 | 41.8537 |
| MCZ | 106432 | *Lithobates sylvaticus* | Male | 1983 | -72.8225 | 42.5401 |
| MCZ | 106433 | *Lithobates sylvaticus* | Male | 1983 | -72.8225 | 42.5401 |
| MCZ | 106435 | *Lithobates sylvaticus* | Male | 1983 | -72.8225 | 42.5401 |
| MCZ | 106441 | *Lithobates sylvaticus* | Female | 1983 | -72.8995 | 42.5551 |
| MCZ | 106997 | *Lithobates sylvaticus* | Male | 1982 | -71.2094 | 41.6278 |
| MCZ | 112852 | *Lithobates sylvaticus* | Male | 1975 | -70.8175 | 42.5811 |
| MCZ | 134944 | *Lithobates sylvaticus* | Male | 1968 | -71.7649 | 42.2372 |
| MCZ | 134945 | *Lithobates sylvaticus* | Male | 1968 | -71.7649 | 42.2372 |
| MCZ | 134957 | *Lithobates sylvaticus* | Male | 1968 | -71.9925 | 42.3561 |
| MCZ | 134958 | *Lithobates sylvaticus* | Female | 1968 | -71.9925 | 42.3561 |
| MCZ | 134994 | *Lithobates sylvaticus* | Female | 1964 | -72.0125 | 42.6406 |
| MPM | 33216 | *Lithobates sylvaticus* | Female | 1998 | -90.5997 | 46.9286 |
| MPM | 33251 | *Lithobates sylvaticus* | Male | 1999 | -90.5684 | 47.0325 |
| MPM | 33262 | *Lithobates sylvaticus* | Male | 1998 | -90.5000 | 46.8816 |
| MVZ | 36181 | *Lithobates sylvaticus* | Female | 1940 | -147.8030 | 64.8570 |
| MVZ | 49385 | *Lithobates sylvaticus* | Male | 1949 | -106.5215 | 40.4012 |
| MVZ | 49386 | *Lithobates sylvaticus* | Female | 1949 | -106.5215 | 40.4012 |
| MVZ | 49387 | *Lithobates sylvaticus* | Male | 1949 | -106.5215 | 40.4012 |
| MVZ | 49388 | *Lithobates sylvaticus* | Female | 1949 | -106.5215 | 40.4012 |
| MVZ | 53669 | *Lithobates sylvaticus* | Male | 1950 | -77.5674 | 43.2370 |
| MVZ | 60482 | *Lithobates sylvaticus* | Male | 1952 | -86.2678 | 41.6471 |
| MVZ | 60483 | *Lithobates sylvaticus* | Female | 1952 | -86.2678 | 41.6471 |
| MVZ | 60484 | *Lithobates sylvaticus* | Male | 1952 | -86.2678 | 41.6471 |
| MVZ | 60485 | *Lithobates sylvaticus* | Female | 1952 | -86.2678 | 41.6471 |
| MVZ | 60486 | *Lithobates sylvaticus* | Female | 1952 | -86.2678 | 41.6471 |
| MVZ | 60487 | *Lithobates sylvaticus* | Female | 1952 | -86.2678 | 41.6471 |
| MVZ | 60488 | *Lithobates sylvaticus* | Male | 1952 | -86.2678 | 41.6471 |
| MVZ | 60489 | *Lithobates sylvaticus* | Male | 1952 | -86.2678 | 41.6471 |
| MVZ | 60490 | *Lithobates sylvaticus* | Male | 1952 | -86.2678 | 41.6471 |
| MVZ | 68071 | *Lithobates sylvaticus* | Male | 1954 | -149.9064 | 61.1886 |
| MVZ | 68074 | *Lithobates sylvaticus* | Male | 1953 | -149.9064 | 61.1886 |
| MVZ | 68087 | *Lithobates sylvaticus* | Female | 1954 | -149.9064 | 61.1886 |
| MVZ | 68089 | *Lithobates sylvaticus* | Male | 1953 | -149.9064 | 61.1886 |
| MVZ | 68093 | *Lithobates sylvaticus* | Male | 1953 | -149.9064 | 61.1886 |
| MVZ | 68098 | *Lithobates sylvaticus* | Female | 1953 | -144.8459 | 62.8204 |
| MVZ | 68100 | *Lithobates sylvaticus* | Male | 1954 | -149.8172 | 61.1950 |
| MVZ | 68116 | *Lithobates sylvaticus* | Female | 1954 | -149.9064 | 61.1886 |
| MVZ | 68117 | *Lithobates sylvaticus* | Male | 1954 | -149.9064 | 61.1886 |
| MVZ | 68580 | *Lithobates sylvaticus* | Female | 1955 | -161.7558 | 60.7922 |
| MVZ | 68588 | *Lithobates sylvaticus* | Female | 1956 | -150.8983 | 63.5189 |
| MVZ | 68610 | *Lithobates sylvaticus* | Female | 1957 | -133.7706 | 58.4881 |
| MVZ | 68973 | *Lithobates sylvaticus* | Male | 1959 | -147.8706 | 64.8569 |
| MVZ | 68974 | *Lithobates sylvaticus* | Male | 1959 | -145.3306 | 62.2966 |
| MVZ | 68975 | *Lithobates sylvaticus* | Male | 1959 | -145.3306 | 62.2966 |
| MVZ | 68976 | *Lithobates sylvaticus* | Male | 1959 | -145.3306 | 62.2966 |
| MVZ | 68977 | *Lithobates sylvaticus* | Female | 1959 | -145.3306 | 62.2966 |
| MVZ | 68978 | *Lithobates sylvaticus* | Male | 1959 | -145.3306 | 62.2966 |
| MVZ | 68980 | *Lithobates sylvaticus* | Male | 1959 | -145.3306 | 62.2966 |
| MVZ | 68981 | *Lithobates sylvaticus* | Male | 1959 | -145.3306 | 62.2966 |
| MVZ | 68982 | *Lithobates sylvaticus* | Female | 1959 | -145.3306 | 62.2966 |
| MVZ | 68983 | *Lithobates sylvaticus* | Male | 1959 | -145.3306 | 62.2966 |
| MVZ | 68984 | *Lithobates sylvaticus* | Female | 1959 | -145.3306 | 62.2966 |
| MVZ | 68985 | *Lithobates sylvaticus* | Female | 1959 | -145.3306 | 62.2966 |
| MVZ | 68986 | *Lithobates sylvaticus* | Male | 1959 | -145.3306 | 62.2966 |
| MVZ | 68987 | *Lithobates sylvaticus* | Male | 1959 | -145.3306 | 62.2966 |
| MVZ | 68988 | *Lithobates sylvaticus* | Male | 1959 | -145.3306 | 62.2966 |
| MVZ | 68990 | *Lithobates sylvaticus* | Female | 1959 | -145.3924 | 62.0747 |
| MVZ | 69037 | *Lithobates sylvaticus* | Female | 1959 | -145.3506 | 62.2890 |
| MVZ | 69044 | *Lithobates sylvaticus* | Male | 1959 | -117.4653 | 54.9639 |
| MVZ | 69045 | *Lithobates sylvaticus* | Female | 1959 | -117.4653 | 54.9639 |
| MVZ | 69046 | *Lithobates sylvaticus* | Female | 1959 | -117.4653 | 54.9639 |
| MVZ | 69048 | *Lithobates sylvaticus* | Female | 1959 | -117.4653 | 54.9639 |
| MVZ | 71678 | *Lithobates sylvaticus* | Female | 1959 | -139.7272 | 59.5469 |
| MVZ | 98022 | *Lithobates sylvaticus* | Female | 1964 | -81.8706 | 36.0664 |
| MVZ | 105240 | *Lithobates sylvaticus* | Female | 1972 | -133.9667 | 60.3030 |
| MVZ | 105251 | *Lithobates sylvaticus* | Female | 1972 | -135.6992 | 59.2969 |
| MVZ | 105252 | *Lithobates sylvaticus* | Female | 1972 | -135.6992 | 59.2969 |
| MVZ | 105254 | *Lithobates sylvaticus* | Female | 1972 | -135.6992 | 59.2969 |
| MVZ | 105255 | *Lithobates sylvaticus* | Female | 1972 | -135.6992 | 59.2969 |
| MVZ | 105256 | *Lithobates sylvaticus* | Male | 1972 | -135.6992 | 59.2969 |
| MVZ | 105257 | *Lithobates sylvaticus* | Female | 1972 | -135.6992 | 59.2969 |
| MVZ | 105273 | *Lithobates sylvaticus* | Male | 1972 | -142.9844 | 63.3366 |
| MVZ | 105274 | *Lithobates sylvaticus* | Male | 1972 | -149.4422 | 60.2875 |
| MVZ | 105275 | *Lithobates sylvaticus* | Female | 1972 | -149.4422 | 60.2875 |
| MVZ | 105276 | *Lithobates sylvaticus* | Male | 1972 | -149.4422 | 60.2875 |
| MVZ | 105277 | *Lithobates sylvaticus* | Female | 1972 | -149.4422 | 60.2875 |
| MVZ | 128867 | *Lithobates sylvaticus* | Male | 1975 | -83.7077 | 35.6581 |
| MVZ | 212074 | *Lithobates sylvaticus* | Female | 1952 | -86.2678 | 41.6471 |
| NCMNS | 517 | *Lithobates sylvaticus* | Female | 1922 | -73.7536 | 42.6481 |
| NCMNS | 4387 | *Lithobates sylvaticus* | Male | 1966 | -76.3990 | 42.4402 |
| NCMNS | 4388 | *Lithobates sylvaticus* | Male | 1966 | -76.3990 | 42.4402 |
| NCMNS | 5474 | *Lithobates sylvaticus* | Male | 1967 | -82.2957 | 35.4409 |
| NCMNS | 12236 | *Lithobates sylvaticus* | Male | 1973 | -83.1833 | 35.2756 |
| NCMNS | 12237 | *Lithobates sylvaticus* | Male | 1973 | -83.1833 | 35.2756 |
| NCMNS | 12238 | *Lithobates sylvaticus* | Male | 1973 | -83.1833 | 35.2756 |
| NCMNS | 12239 | *Lithobates sylvaticus* | Male | 1973 | -83.1833 | 35.2756 |
| NCMNS | 12766 | *Lithobates sylvaticus* | Female | 1973 | -83.2007 | 35.0542 |
| NCMNS | 14524 | *Lithobates sylvaticus* | Female | 1974 | -83.1886 | 35.0499 |
| NCMNS | 18061 | *Lithobates sylvaticus* | Male | 1977 | -83.8884 | 35.4134 |
| NCMNS | 18062 | *Lithobates sylvaticus* | Male | 1977 | -83.8884 | 35.4134 |
| NCMNS | 22624 | *Lithobates sylvaticus* | Female | 1982 | -79.9934 | 36.5358 |
| NCMNS | 25471 | *Lithobates sylvaticus* | Female | 1982 | -88.1802 | 43.5223 |
| NCMNS | 26150 | *Lithobates sylvaticus* | Male | 1984 | -76.1213 | 35.9721 |
| NCMNS | 27377 | *Lithobates sylvaticus* | Female | 1967 | -75.2559 | 44.3736 |
| NCMNS | 31359 | *Lithobates sylvaticus* | Female | 1991 | -76.4397 | 35.5373 |
| NCMNS | 31419 | *Lithobates sylvaticus* | Male | 1992 | -76.4400 | 35.5444 |
| NCMNS | 31833 | *Lithobates sylvaticus* | Male | 1992 | -80.9242 | 36.4260 |
| NCMNS | 35390 | *Lithobates sylvaticus* | Female | 1971 | -83.1807 | 35.7113 |
| NCMNS | 52386 | *Lithobates sylvaticus* | Female | 1975 | -81.0000 | 36.0000 |
| NCMNS | 52387 | *Lithobates sylvaticus* | Female | 1975 | -81.0000 | 36.0000 |
| NCMNS | 52388 | *Lithobates sylvaticus* | Male | 1962 | -82.3328 | 35.2190 |
| NCMNS | 52389 | *Lithobates sylvaticus* | Male | 1962 | -82.3328 | 35.2190 |
| NCMNS | 52390 | *Lithobates sylvaticus* | Female | 1962 | -82.3328 | 35.2190 |
| NCMNS | 52394 | *Lithobates sylvaticus* | Male | 1967 | -81.3236 | 35.9750 |
| NCMNS | 52398 | *Lithobates sylvaticus* | Male | 1962 | -82.2663 | 35.4615 |
| NCMNS | 52407 | *Lithobates sylvaticus* | Male | 1962 | -71.2076 | 47.5392 |
| NCMNS | 52408 | *Lithobates sylvaticus* | Male | 1962 | -71.2076 | 47.5392 |
| NCMNS | 52409 | *Lithobates sylvaticus* | Male | 1962 | -71.2076 | 47.5392 |
| NCMNS | 52410 | *Lithobates sylvaticus* | Male | 1962 | -71.2076 | 47.5392 |
| NCMNS | 52421 | *Lithobates sylvaticus* | Male | 1956 | -81.4398 | 41.2398 |
| NCMNS | 52425 | *Lithobates sylvaticus* | Male | 1965 | -79.5206 | 38.6454 |
| NCMNS | 52426 | *Lithobates sylvaticus* | Female | 1965 | -79.5900 | 38.6211 |
| NCMNS | 56294 | *Lithobates sylvaticus* | Male | 1952 | -81.6158 | 35.9673 |
| NCMNS | 56295 | *Lithobates sylvaticus* | Male | 1952 | -81.6158 | 35.9673 |
| NCMNS | 56296 | *Lithobates sylvaticus* | Male | 1954 | -80.9789 | 36.0814 |
| NCMNS | 56297 | *Lithobates sylvaticus* | Female | 1954 | -80.9789 | 36.0814 |
| NCMNS | 56298 | *Lithobates sylvaticus* | Female | 1954 | -80.9789 | 36.0814 |
| NCMNS | 56300 | *Lithobates sylvaticus* | Male | 1955 | -82.8896 | 35.1289 |
| NCMNS | 56306 | *Lithobates sylvaticus* | Female | 1970 | -81.8164 | 35.6058 |
| NCMNS | 60883 | *Lithobates sylvaticus* | Male | 1999 | -74.6056 | 39.5573 |
| NCMNS | 60884 | *Lithobates sylvaticus* | Male | 1999 | -74.6056 | 39.5573 |
| NCMNS | 61737 | *Lithobates sylvaticus* | Female | 1973 | -76.2165 | 39.0053 |
| NCMNS | 63263 | *Lithobates sylvaticus* | Female | 1967 | -76.0216 | 42.0321 |
| NCMNS | 63264 | *Lithobates sylvaticus* | Female | 1967 | -76.0216 | 42.0321 |
| NCMNS | 63265 | *Lithobates sylvaticus* | Male | 1967 | -76.0216 | 42.0321 |
| NCMNS | 63266 | *Lithobates sylvaticus* | Male | 1967 | -76.0216 | 42.0321 |
| NCMNS | 63268 | *Lithobates sylvaticus* | Male | 1967 | -76.0216 | 42.0321 |
| NCMNS | 63269 | *Lithobates sylvaticus* | Male | 1967 | -76.0216 | 42.0321 |
| NCMNS | 63270 | *Lithobates sylvaticus* | Male | 1967 | -76.0216 | 42.0321 |
| NCMNS | 63271 | *Lithobates sylvaticus* | Male | 1967 | -76.0216 | 42.0321 |
| NCMNS | 63272 | *Lithobates sylvaticus* | Male | 1967 | -76.0216 | 42.0321 |
| NCMNS | 63273 | *Lithobates sylvaticus* | Male | 1967 | -76.0216 | 42.0321 |
| NCMNS | 63274 | *Lithobates sylvaticus* | Male | 1967 | -76.0216 | 42.0321 |
| NCMNS | 63275 | *Lithobates sylvaticus* | Female | 1969 | -76.0537 | 42.0851 |
| NCMNS | 63276 | *Lithobates sylvaticus* | Female | 1969 | -76.0537 | 42.0851 |
| NCMNS | 63277 | *Lithobates sylvaticus* | Male | 1969 | -76.0537 | 42.0851 |
| NCMNS | 63789 | *Lithobates sylvaticus* | Female | 1967 | -76.0212 | 42.0308 |
| NCMNS | 65528 | *Lithobates sylvaticus* | Male | 1996 | -79.6397 | 38.5826 |
| NCMNS | 67854 | *Lithobates sylvaticus* | Male | 1973 | -79.8871 | 38.1022 |
| NCMNS | 67857 | *Lithobates sylvaticus* | Male | 1973 | -79.8871 | 38.1022 |
| NCMNS | 67858 | *Lithobates sylvaticus* | Male | 1975 | -77.6425 | 37.7696 |
| NCMNS | 67860 | *Lithobates sylvaticus* | Female | 1975 | -79.0000 | 38.0000 |
| NCMNS | 67861 | *Lithobates sylvaticus* | Female | 1975 | -81.5411 | 36.6869 |
| NCMNS | 67863 | *Lithobates sylvaticus* | Female | 1976 | -79.3149 | 37.7323 |
| NCMNS | 67866 | *Lithobates sylvaticus* | Female | 1979 | -78.2005 | 38.8925 |
| NCMNS | 67867 | *Lithobates sylvaticus* | Female | 1979 | -78.2061 | 38.8738 |
| NCMNS | 67868 | *Lithobates sylvaticus* | Female | 1979 | -78.2061 | 38.8738 |
| NCMNS | 67869 | *Lithobates sylvaticus* | Male | 1979 | -78.2057 | 38.8497 |
| NCMNS | 67870 | *Lithobates sylvaticus* | Female | 1979 | -78.2057 | 38.8497 |
| NCMNS | 69049 | *Lithobates sylvaticus* | Male | 1980 | -80.5979 | 37.2172 |
| PSM | 7966 | *Lithobates sylvaticus* | Female | 1965 | -115.9926 | 53.6659 |
| PSM | 7968 | *Lithobates sylvaticus* | Female | 1965 | -115.9926 | 53.6659 |
| PSM | 7969 | *Lithobates sylvaticus* | Female | 1965 | -115.9926 | 53.6659 |
| PSM | 9153 | *Lithobates sylvaticus* | Male | 1958 | -145.2797 | 66.5807 |
| RAM | 89.19.29 | *Lithobates sylvaticus* | Male | 1989 | -110.5200 | 55.4300 |
| RAM | 89.19.30 | *Lithobates sylvaticus* | Male | 1989 | -110.5200 | 55.4300 |
| RAM | 89.19.30-a | *Lithobates sylvaticus* | Male | 1989 | -110.5200 | 55.4300 |
| RAM | L92.15.2 | *Lithobates sylvaticus* | Male | 1969 | -113.0700 | 53.5200 |
| RAM | L93.13.30 | *Lithobates sylvaticus* | Female | 1993 | -119.1500 | 57.3700 |
| RAM | L93.13.31 | *Lithobates sylvaticus* | Male | 1969 | -113.0700 | 53.5200 |
| RAM | L93.19.3 | *Lithobates sylvaticus* | Female | 1995 | -119.4000 | 57.1200 |
| RAM | L93.19.4 | *Lithobates sylvaticus* | Female | 1996 | -119.4000 | 57.1200 |
| RAM | L93.19.5 | *Lithobates sylvaticus* | Male | 1971 | -115.4300 | 56.4000 |
| RAM | L93.20.24 | *Lithobates sylvaticus* | Female | 1993 | -114.9300 | 53.2000 |
| RAM | L93.20.26 | *Lithobates sylvaticus* | Female | 1993 | -114.7200 | 53.2500 |
| RAM | L94.20.50 | *Lithobates sylvaticus* | Female | 1994 | -116.0700 | 52.4000 |
| RAM | L94.24.39 | *Lithobates sylvaticus* | Female | 1994 | -119.8000 | 57.3500 |
| RAM | L94.24.39-a | *Lithobates sylvaticus* | Female | 1994 | -119.8000 | 57.3500 |
| RAM | L94.24.40 | *Lithobates sylvaticus* | Female | 1994 | -119.7800 | 57.3300 |
| RAM | L95.17.60 | *Lithobates sylvaticus* | Male | 1971 | -115.1700 | 55.8300 |
| RAM | L95.17.60-a | *Lithobates sylvaticus* | Male | 1971 | -115.1700 | 55.8300 |
| RAM | L95.17.60-b | *Lithobates sylvaticus* | Male | 1971 | -113.0200 | 52.3300 |
| RAM | L95.17.60-c | *Lithobates sylvaticus* | Male | 1992 | -116.7700 | 53.0300 |
| RAM | L96.2.190 | *Lithobates sylvaticus* | Female | 1996 | -111.0500 | 59.1700 |
| RAM | L97.28.2 | *Lithobates sylvaticus* | Female | 1997 | -114.7300 | 58.4000 |
| RAM | Z69.45.1 | *Lithobates sylvaticus* | Male | 1993 | -119.1500 | 57.3700 |
| RAM | Z69.45.6 | *Lithobates sylvaticus* | Male | 1995 | -115.4800 | 58.9500 |
| RAM | Z69.50.2 | *Lithobates sylvaticus* | Female | 1969 | -116.8000 | 53.0800 |
| RAM | Z71.37.77 | *Lithobates sylvaticus* | Male | 1995 | -115.4800 | 58.9500 |
| RAM | Z71.37.78 | *Lithobates sylvaticus* | Male | 1995 | -115.4800 | 58.9500 |
| RAM | Z71.41.4 | *Lithobates sylvaticus* | Female | 1971 | -115.4300 | 56.4000 |
| RAM | Z71.41.5 | *Lithobates sylvaticus* | Male | 1995 | -115.4800 | 58.9500 |
| RAM | Z71.62.3 | *Lithobates sylvaticus* | Male | 1997 | -119.4800 | 57.7800 |
| RAM | Z78.79.2 | *Lithobates sylvaticus* | Female | 1978 | -114.3500 | 50.3800 |
| RMMU | 2743 | *Lithobates sylvaticus* | Female | 1992 | -73.9506 | 45.4320 |
| RMMU | 2765 | *Lithobates sylvaticus* | Male | 1990 | -66.0644 | 48.5018 |
| RMMU | 2766 | *Lithobates sylvaticus* | Male | 1990 | -73.5974 | 45.4988 |
| RMMU | 2767 | *Lithobates sylvaticus* | Male | 1990 | -73.5974 | 45.4988 |
| RMMU | 2768 | *Lithobates sylvaticus* | Male | 1990 | -73.5974 | 45.4988 |
| RMMU | 2769 | *Lithobates sylvaticus* | Male | 1990 | -73.5974 | 45.4988 |
| RMMU | 2770 | *Lithobates sylvaticus* | Male | 1990 | -73.5974 | 45.4988 |
| RMMU | 2771 | *Lithobates sylvaticus* | Male | 1990 | -73.5974 | 45.4988 |
| RMMU | 2772 | *Lithobates sylvaticus* | Female | 1990 | -73.5974 | 45.4988 |
| RMMU | 2773 | *Lithobates sylvaticus* | Male | 1990 | -73.5974 | 45.4988 |
| RMMU | 2774 | *Lithobates sylvaticus* | Male | 1990 | -73.5974 | 45.4988 |
| RMMU | 2822 | *Lithobates sylvaticus* | Male | 1991 | -73.5002 | 45.4979 |
| RMMU | 2854 | *Lithobates sylvaticus* | Male | 1990 | -73.3418 | 45.5306 |
| RMMU | 2855 | *Lithobates sylvaticus* | Male | 1990 | -73.3418 | 45.5306 |
| RMMU | 2856 | *Lithobates sylvaticus* | Female | 1990 | -73.3418 | 45.5306 |
| RMMU | 2857 | *Lithobates sylvaticus* | Female | 1990 | -73.3418 | 45.5306 |
| RMMU | 2858 | *Lithobates sylvaticus* | Male | 1990 | -73.3418 | 45.5306 |
| RMMU | 2859 | *Lithobates sylvaticus* | Male | 1990 | -73.3418 | 45.5306 |
| RMMU | 2860 | *Lithobates sylvaticus* | Male | 1990 | -73.3418 | 45.5306 |
| RMMU | 2861 | *Lithobates sylvaticus* | Male | 1990 | -73.3418 | 45.5306 |
| RMMU | 2862 | *Lithobates sylvaticus* | Male | 1990 | -73.3418 | 45.5306 |
| RMMU | 2863 | *Lithobates sylvaticus* | Female | 1990 | -73.3418 | 45.5306 |
| RMMU | 2864 | *Lithobates sylvaticus* | Male | 1990 | -73.3418 | 45.5306 |
| RMMU | 3033 | *Lithobates sylvaticus* | Male | 1986 | -72.2989 | 45.1536 |
| RMMU | 3187 | *Lithobates sylvaticus* | Female | 1998 | -73.9506 | 45.4320 |
| SDNHM | 24486 | *Lithobates sylvaticus* | Male | 1934 | -87.0386 | 41.6627 |
| SDNHM | 24490 | *Lithobates sylvaticus* | Male | 1934 | -87.0386 | 41.6627 |
| SDNHM | 65270 | *Lithobates sylvaticus* | Female | 1985 | -74.1372 | 41.7403 |
| SDNHM | 65271 | *Lithobates sylvaticus* | Male | 1985 | -74.1626 | 41.7657 |
| SDNHM | 65272 | *Lithobates sylvaticus* | Female | 1985 | -73.7342 | 43.3925 |
| SDNHM | 65274 | *Lithobates sylvaticus* | Female | 1985 | -73.7936 | 43.3647 |
| SMNH | 24117 | *Lithobates sylvaticus* | Male | 1987 | -68.5697 | 44.2661 |
| SMNH | 29306 | *Lithobates sylvaticus* | Female | 1901 | -78.3800 | 38.5983 |
| SMNH | 32482 | *Lithobates sylvaticus* | Female | 1903 | -77.1769 | 38.9708 |
| SMNH | 33137 | *Lithobates sylvaticus* | Male | 1902 | -91.7986 | 46.2469 |
| SMNH | 33138 | *Lithobates sylvaticus* | Male | 1902 | -89.9733 | 46.2831 |
| SMNH | 33784 | *Lithobates sylvaticus* | Male | 1902 | -85.8586 | 40.5014 |
| SMNH | 34567 | *Lithobates sylvaticus* | Female | 1904 | -77.3000 | 38.8333 |
| SMNH | 35447 | *Lithobates sylvaticus* | Female | 1901 | -86.4036 | 41.2022 |
| SMNH | 35510 | *Lithobates sylvaticus* | Male | 1904 | -99.3625 | 49.8717 |
| SMNH | 35600 | *Lithobates sylvaticus* | Male | 1903 | -89.7500 | 46.7672 |
| SMNH | 37960 | *Lithobates sylvaticus* | Female | 1907 | -92.9883 | 45.1761 |
| SMNH | 37976 | *Lithobates sylvaticus* | Male | 1907 | -88.0828 | 38.7311 |
| SMNH | 42647 | *Lithobates sylvaticus* | Male | 1906 | -85.1314 | 41.0717 |
| SMNH | 42703 | *Lithobates sylvaticus* | Female | 1909 | -84.3872 | 42.2322 |
| SMNH | 46110 | *Lithobates sylvaticus* | Male | 1904 | -121.3547 | 61.8642 |
| SMNH | 47971 | *Lithobates sylvaticus* | Female | 1901 | -111.8817 | 60.0086 |
| SMNH | 47972 | *Lithobates sylvaticus* | Male | 1901 | -111.8817 | 60.0086 |
| SMNH | 47975 | *Lithobates sylvaticus* | Female | 1901 | -111.8817 | 60.0086 |
| SMNH | 48002 | *Lithobates sylvaticus* | Female | 1901 | -113.6681 | 61.1697 |
| SMNH | 48012 | *Lithobates sylvaticus* | Female | 1901 | -113.6681 | 61.1697 |
| SMNH | 48014 | *Lithobates sylvaticus* | Female | 1901 | -113.6681 | 61.1697 |
| SMNH | 48015 | *Lithobates sylvaticus* | Female | 1901 | -113.6681 | 61.1697 |
| SMNH | 49665 | *Lithobates sylvaticus* | Female | 1901 | -113.6681 | 61.1697 |
| SMNH | 49670 | *Lithobates sylvaticus* | Male | 1907 | -77.1764 | 38.9697 |
| SMNH | 49671 | *Lithobates sylvaticus* | Female | 1907 | -77.1764 | 38.9697 |
| SMNH | 49676 | *Lithobates sylvaticus* | Male | 1907 | -77.1764 | 38.9697 |
| SMNH | 49677 | *Lithobates sylvaticus* | Male | 1907 | -77.1764 | 38.9697 |
| SMNH | 49724 | *Lithobates sylvaticus* | Male | 1907 | -77.1764 | 38.9697 |
| SMNH | 52452 | *Lithobates sylvaticus* | Female | 1915 | -99.7106 | 48.9450 |
| SMNH | 53106 | *Lithobates sylvaticus* | Female | 1915 | -100.4092 | 48.4594 |
| SMNH | 53107 | *Lithobates sylvaticus* | Female | 1915 | -100.4092 | 48.4594 |
| SMNH | 53109 | *Lithobates sylvaticus* | Female | 1915 | -100.4092 | 48.4594 |
| SMNH | 53110 | *Lithobates sylvaticus* | Male | 1915 | -100.4092 | 48.4594 |
| SMNH | 53121 | *Lithobates sylvaticus* | Male | 1915 | -97.1803 | 48.0769 |
| SMNH | 55159 | *Lithobates sylvaticus* | Female | 1916 | -81.8572 | 36.0669 |
| SMNH | 57926 | *Lithobates sylvaticus* | Male | 1911 | -83.7422 | 42.2694 |
| SMNH | 57927 | *Lithobates sylvaticus* | Female | 1911 | -83.7422 | 42.2694 |
| SMNH | 63936 | *Lithobates sylvaticus* | Female | 1920 | -107.7814 | 59.3347 |
| SMNH | 82325 | *Lithobates sylvaticus* | Female | 1909 | -108.5225 | 45.7244 |
| SMNH | 82502 | *Lithobates sylvaticus* | Female | 1904 | -73.8156 | 44.3158 |
| SMNH | 103314 | *Lithobates sylvaticus* | Female | 1937 | -72.6850 | 44.4653 |
| SMNH | 103315 | *Lithobates sylvaticus* | Female | 1937 | -72.6850 | 44.4653 |
| SMNH | 107252 | *Lithobates sylvaticus* | Male | 1937 | -74.1983 | 39.9536 |
| SMNH | 108710 | *Lithobates sylvaticus* | Male | 1939 | -72.6850 | 44.4653 |
| SMNH | 127396 | *Lithobates sylvaticus* | Female | 1939 | -77.1561 | 39.0122 |
| SMNH | 127397 | *Lithobates sylvaticus* | Male | 1939 | -77.1561 | 39.0122 |
| SMNH | 127398 | *Lithobates sylvaticus* | Male | 1939 | -77.1561 | 39.0122 |
| SMNH | 127399 | *Lithobates sylvaticus* | Male | 1939 | -77.1561 | 39.0122 |
| SMNH | 127400 | *Lithobates sylvaticus* | Male | 1939 | -77.1561 | 39.0122 |
| SMNH | 127531 | *Lithobates sylvaticus* | Male | 1946 | -80.2300 | 37.6800 |
| SMNH | 127533 | *Lithobates sylvaticus* | Male | 1947 | -79.8200 | 37.8400 |
| SMNH | 134417 | *Lithobates sylvaticus* | Male | 1953 | -84.0347 | 35.0875 |
| SMNH | 134418 | *Lithobates sylvaticus* | Female | 1953 | -84.0347 | 35.0875 |
| SMNH | 134505 | *Lithobates sylvaticus* | Female | 1925 | -84.6975 | 45.5808 |
| SMNH | 136718 | *Lithobates sylvaticus* | Male | 1954 | -77.3400 | 39.0000 |
| SMNH | 136719 | *Lithobates sylvaticus* | Male | 1954 | -77.3400 | 39.0000 |
| SMNH | 136720 | *Lithobates sylvaticus* | Male | 1954 | -77.3400 | 39.0000 |
| SMNH | 138539 | *Lithobates sylvaticus* | Male | 1956 | -78.4328 | 38.5179 |
| SMNH | 138762 | *Lithobates sylvaticus* | Male | 1955 | -77.0936 | 39.0150 |
| SMNH | 141029 | *Lithobates sylvaticus* | Female | 1950 | -78.7625 | 39.6528 |
| SMNH | 141030 | *Lithobates sylvaticus* | Male | 1950 | -78.7625 | 39.6528 |
| SMNH | 141031 | *Lithobates sylvaticus* | Female | 1950 | -78.7625 | 39.6528 |
| SMNH | 141032 | *Lithobates sylvaticus* | Male | 1950 | -78.7625 | 39.6528 |
| SMNH | 141033 | *Lithobates sylvaticus* | Male | 1948 | -76.8375 | 39.0717 |
| SMNH | 141036 | *Lithobates sylvaticus* | Female | 1950 | -77.2108 | 39.4406 |
| SMNH | 141038 | *Lithobates sylvaticus* | Male | 1949 | -76.9369 | 38.9828 |
| SMNH | 141039 | *Lithobates sylvaticus* | Male | 1949 | -76.9369 | 38.9828 |
| SMNH | 141040 | *Lithobates sylvaticus* | Male | 1949 | -76.9369 | 38.9828 |
| SMNH | 141042 | *Lithobates sylvaticus* | Female | 1949 | -76.9369 | 38.9828 |
| SMNH | 141053 | *Lithobates sylvaticus* | Female | 1949 | -76.9369 | 38.9808 |
| SMNH | 141054 | *Lithobates sylvaticus* | Male | 1949 | -76.9369 | 38.9808 |
| SMNH | 141056 | *Lithobates sylvaticus* | Male | 1949 | -76.9369 | 38.9808 |
| SMNH | 141058 | *Lithobates sylvaticus* | Female | 1949 | -76.9369 | 38.9808 |
| SMNH | 141059 | *Lithobates sylvaticus* | Female | 1949 | -76.9369 | 38.9808 |
| SMNH | 141061 | *Lithobates sylvaticus* | Male | 1949 | -76.9369 | 38.9808 |
| SMNH | 141062 | *Lithobates sylvaticus* | Male | 1949 | -76.9369 | 38.9808 |
| SMNH | 141065 | *Lithobates sylvaticus* | Male | 1949 | -76.9369 | 38.9808 |
| SMNH | 141066 | *Lithobates sylvaticus* | Male | 1949 | -76.9369 | 38.9808 |
| SMNH | 141069 | *Lithobates sylvaticus* | Male | 1949 | -76.9369 | 38.9808 |
| SMNH | 141070 | *Lithobates sylvaticus* | Male | 1949 | -76.9369 | 38.9808 |
| SMNH | 141076 | *Lithobates sylvaticus* | Male | 1949 | -76.9369 | 38.9808 |
| SMNH | 141077 | *Lithobates sylvaticus* | Male | 1949 | -76.9369 | 38.9808 |
| SMNH | 143937 | *Lithobates sylvaticus* | Female | 1952 | -77.3167 | 38.8500 |
| SMNH | 143938 | *Lithobates sylvaticus* | Female | 1951 | -80.4692 | 37.2808 |
| SMNH | 143939 | *Lithobates sylvaticus* | Male | 1951 | -80.4692 | 37.2808 |
| SMNH | 143940 | *Lithobates sylvaticus* | Female | 1951 | -80.4692 | 37.2808 |
| SMNH | 143941 | *Lithobates sylvaticus* | Female | 1951 | -80.4692 | 37.2808 |
| SMNH | 143942 | *Lithobates sylvaticus* | Male | 1951 | -80.4692 | 37.2808 |
| SMNH | 143943 | *Lithobates sylvaticus* | Male | 1951 | -80.4692 | 37.2808 |
| SMNH | 143944 | *Lithobates sylvaticus* | Male | 1951 | -80.4692 | 37.2808 |
| SMNH | 143945 | *Lithobates sylvaticus* | Male | 1951 | -80.4692 | 37.2808 |
| SMNH | 143946 | *Lithobates sylvaticus* | Male | 1951 | -80.4692 | 37.2808 |
| SMNH | 143947 | *Lithobates sylvaticus* | Female | 1951 | -80.4692 | 37.2808 |
| SMNH | 143948 | *Lithobates sylvaticus* | Male | 1951 | -80.4692 | 37.2808 |
| SMNH | 144280 | *Lithobates sylvaticus* | Female | 1959 | -76.6156 | 39.5786 |
| SMNH | 149968 | *Lithobates sylvaticus* | Male | 1947 | -99.9742 | 60.6331 |
| SMNH | 149969 | *Lithobates sylvaticus* | Female | 1947 | -99.9742 | 60.6331 |
| SMNH | 149971 | *Lithobates sylvaticus* | Male | 1947 | -66.8172 | 54.7953 |
| SMNH | 149973 | *Lithobates sylvaticus* | Female | 1945 | -133.3031 | 60.4869 |
| SMNH | 149974 | *Lithobates sylvaticus* | Female | 1945 | -133.3031 | 60.4869 |
| SMNH | 149976 | *Lithobates sylvaticus* | Male | 1947 | -94.1400 | 58.7464 |
| SMNH | 162683 | *Lithobates sylvaticus* | Male | 1967 | -76.8756 | 39.0047 |
| SMNH | 162684 | *Lithobates sylvaticus* | Female | 1967 | -76.8756 | 39.0047 |
| SMNH | 165744 | *Lithobates sylvaticus* | Male | 1967 | -66.1350 | 45.4728 |
| SMNH | 165745 | *Lithobates sylvaticus* | Male | 1967 | -66.1350 | 45.4728 |
| SMNH | 165746 | *Lithobates sylvaticus* | Male | 1967 | -66.1350 | 45.4728 |
| SMNH | 165747 | *Lithobates sylvaticus* | Male | 1967 | -66.1350 | 45.4728 |
| SMNH | 166435 | *Lithobates sylvaticus* | Male | 1968 | -106.1511 | 41.0806 |
| SMNH | 166438 | *Lithobates sylvaticus* | Female | 1962 | -106.1411 | 41.2983 |
| SMNH | 166440 | *Lithobates sylvaticus* | Female | 1962 | -106.1411 | 41.2983 |
| SMNH | 166441 | *Lithobates sylvaticus* | Male | 1962 | -106.1411 | 41.2983 |
| SMNH | 166779 | *Lithobates sylvaticus* | Female | 1968 | -106.1511 | 41.0806 |
| SMNH | 166780 | *Lithobates sylvaticus* | Male | 1965 | -99.3019 | 49.8714 |
| SMNH | 166782 | *Lithobates sylvaticus* | Male | 1965 | -99.3019 | 49.8714 |
| SMNH | 166783 | *Lithobates sylvaticus* | Male | 1967 | -100.0694 | 49.1261 |
| SMNH | 197428 | *Lithobates sylvaticus* | Male | 1971 | -77.0856 | 39.0081 |
| SMNH | 198675 | *Lithobates sylvaticus* | Male | 1974 | -77.3400 | 37.9300 |
| SMNH | 203201 | *Lithobates sylvaticus* | Female | 1975 | -78.4687 | 38.8818 |
| SMNH | 207593 | *Lithobates sylvaticus* | Male | 1976 | -90.5942 | 46.6033 |
| SMNH | 207595 | *Lithobates sylvaticus* | Female | 1976 | -90.6814 | 46.6086 |
| SMNH | 207601 | *Lithobates sylvaticus* | Male | 1976 | -90.4636 | 46.5472 |
| SMNH | 207608 | *Lithobates sylvaticus* | Male | 1976 | -87.5314 | 44.1761 |
| SMNH | 207612 | *Lithobates sylvaticus* | Male | 1976 | -87.5692 | 44.1539 |
| SMNH | 207616 | *Lithobates sylvaticus* | Male | 1976 | -87.5692 | 44.1539 |
| SMNH | 207617 | *Lithobates sylvaticus* | Female | 1976 | -87.5692 | 44.1539 |
| SMNH | 207618 | *Lithobates sylvaticus* | Male | 1976 | -89.6456 | 46.0989 |
| SMNH | 207621 | *Lithobates sylvaticus* | Female | 1976 | -90.2417 | 44.4533 |
| SMNH | 209412 | *Lithobates sylvaticus* | Female | 1977 | -86.1675 | 46.2519 |
| SMNH | 241130 | *Lithobates sylvaticus* | Female | 1977 | -76.9992 | 41.4242 |
| SMNH | 244791 | *Lithobates sylvaticus* | Female | 1950 | -65.8122 | 45.6758 |
| SMNH | 246833 | *Lithobates sylvaticus* | Male | 1979 | -69.6542 | 45.6411 |
| SMNH | 246834 | *Lithobates sylvaticus* | Male | 1976 | -77.3833 | 38.7833 |
| SMNH | 261052 | *Lithobates sylvaticus* | Female | 1948 | -83.6556 | 42.3133 |
| SMNH | 279169 | *Lithobates sylvaticus* | Female | 1981 | -75.7933 | 39.3039 |
| SMNH | 279170 | *Lithobates sylvaticus* | Male | 1981 | -75.7933 | 39.3039 |
| SMNH | 292162 | *Lithobates sylvaticus* | Male | 1986 | -77.2556 | 37.8247 |
| SMNH | 292163 | *Lithobates sylvaticus* | Male | 1986 | -77.2556 | 37.8247 |
| SMNH | 312525 | *Lithobates sylvaticus* | Male | 1931 | -70.7539 | 45.1339 |
| SMNH | 312529 | *Lithobates sylvaticus* | Male | 1929 | -77.1769 | 38.9708 |
| SMNH | 312531 | *Lithobates sylvaticus* | Male | 1933 | -77.1769 | 38.9708 |
| SMNH | 312533 | *Lithobates sylvaticus* | Female | 1935 | -77.1769 | 38.9708 |
| SMNH | 312534 | *Lithobates sylvaticus* | Female | 1935 | -77.1769 | 38.9708 |
| SMNH | 312536 | *Lithobates sylvaticus* | Female | 1926 | -77.1769 | 38.9708 |
| SMNH | 312539 | *Lithobates sylvaticus* | Female | 1928 | -77.1769 | 38.9708 |
| SMNH | 312541 | *Lithobates sylvaticus* | Female | 1926 | -77.1769 | 38.9708 |
| SMNH | 312542 | *Lithobates sylvaticus* | Male | 1926 | -77.1769 | 38.9708 |
| SMNH | 312543 | *Lithobates sylvaticus* | Male | 1926 | -77.1769 | 38.9708 |
| SMNH | 312547 | *Lithobates sylvaticus* | Female | 1941 | -76.8136 | 39.0572 |
| SMNH | 312548 | *Lithobates sylvaticus* | Male | 1941 | -76.8136 | 39.0572 |
| SMNH | 312549 | *Lithobates sylvaticus* | Female | 1941 | -76.8136 | 39.0572 |
| SMNH | 312550 | *Lithobates sylvaticus* | Male | 1941 | -76.8136 | 39.0572 |
| SMNH | 312554 | *Lithobates sylvaticus* | Male | 1943 | -76.8092 | 39.0478 |
| SMNH | 312555 | *Lithobates sylvaticus* | Male | 1943 | -76.8092 | 39.0478 |
| SMNH | 312556 | *Lithobates sylvaticus* | Male | 1943 | -76.8092 | 39.0478 |
| SMNH | 312557 | *Lithobates sylvaticus* | Male | 1943 | -76.8092 | 39.0478 |
| SMNH | 312558 | *Lithobates sylvaticus* | Male | 1943 | -76.8092 | 39.0478 |
| SMNH | 312559 | *Lithobates sylvaticus* | Male | 1943 | -76.8092 | 39.0478 |
| SMNH | 312560 | *Lithobates sylvaticus* | Male | 1943 | -76.8092 | 39.0478 |
| SMNH | 312561 | *Lithobates sylvaticus* | Male | 1943 | -76.8092 | 39.0478 |
| SMNH | 312562 | *Lithobates sylvaticus* | Male | 1943 | -76.8092 | 39.0478 |
| SMNH | 312563 | *Lithobates sylvaticus* | Male | 1943 | -76.8092 | 39.0478 |
| SMNH | 312564 | *Lithobates sylvaticus* | Male | 1943 | -76.8092 | 39.0478 |
| SMNH | 312565 | *Lithobates sylvaticus* | Male | 1943 | -76.8092 | 39.0478 |
| SMNH | 312566 | *Lithobates sylvaticus* | Male | 1931 | -72.5381 | 42.4881 |
| SMNH | 312571 | *Lithobates sylvaticus* | Male | 1928 | -87.8956 | 46.8381 |
| SMNH | 312585 | *Lithobates sylvaticus* | Female | 1929 | -95.8894 | 48.4903 |
| SMNH | 312586 | *Lithobates sylvaticus* | Female | 1929 | -95.8894 | 48.4903 |
| SMNH | 312587 | *Lithobates sylvaticus* | Female | 1929 | -95.8894 | 48.4903 |
| SMNH | 312593 | *Lithobates sylvaticus* | Female | 1931 | -71.2833 | 44.0781 |
| SMNH | 312600 | *Lithobates sylvaticus* | Male | 1928 | -76.9525 | 38.9042 |
| SMNH | 312607 | *Lithobates sylvaticus* | Female | 1929 | -99.7106 | 48.9450 |
| SMNH | 312618 | *Lithobates sylvaticus* | Female | 1929 | -97.3000 | 45.3500 |
| SMNH | 312623 | *Lithobates sylvaticus* | Male | 1929 | -89.7722 | 43.6256 |
| SMNH | 312633 | *Lithobates sylvaticus* | Female | 1931 | -87.1233 | 45.0661 |
| SMNH | 314882 | *Lithobates sylvaticus* | Male | 1990 | -77.1528 | 39.0839 |
| SMNH | 314883 | *Lithobates sylvaticus* | Male | 1990 | -77.1528 | 39.0839 |
| SMNH | 314884 | *Lithobates sylvaticus* | Male | 1990 | -77.1528 | 39.0839 |
| SMNH | 314885 | *Lithobates sylvaticus* | Male | 1990 | -77.1528 | 39.0839 |
| SMNH | 314886 | *Lithobates sylvaticus* | Male | 1990 | -77.1528 | 39.0839 |
| SMNH | 315491 | *Lithobates sylvaticus* | Male | 1991 | -83.2042 | 35.0956 |
| SMNH | 315492 | *Lithobates sylvaticus* | Male | 1991 | -83.1975 | 35.0922 |
| SMNH | 325851 | *Lithobates sylvaticus* | Female | 1990 | -78.3825 | 39.5000 |
| SMNH | 325852 | *Lithobates sylvaticus* | Male | 1990 | -78.3825 | 39.5000 |
| SMNH | 338809 | *Lithobates sylvaticus* | Female | 1980 | -86.8986 | 37.4319 |
| SMNH | 362429 | *Lithobates sylvaticus* | Male | 1959 | -75.6050 | 39.0433 |
| SMNH | 362430 | *Lithobates sylvaticus* | Male | 1959 | -75.6050 | 39.0433 |
| SMNH | 362431 | *Lithobates sylvaticus* | Male | 1959 | -75.6050 | 39.0433 |
| SMNH | 362432 | *Lithobates sylvaticus* | Male | 1959 | -75.6050 | 39.0433 |
| SMNH | 369384 | *Lithobates sylvaticus* | Male | 1960 | -76.7414 | 38.5342 |
| SMNH | 369385 | *Lithobates sylvaticus* | Male | 1960 | -76.7414 | 38.5342 |
| SMNH | 369386 | *Lithobates sylvaticus* | Male | 1960 | -76.7414 | 38.5342 |
| SMNH | 369387 | *Lithobates sylvaticus* | Male | 1960 | -76.7414 | 38.5342 |
| SMNH | 369388 | *Lithobates sylvaticus* | Male | 1960 | -76.7414 | 38.5342 |
| SMNH | 369389 | *Lithobates sylvaticus* | Male | 1960 | -76.7414 | 38.5342 |
| SMNH | 369417 | *Lithobates sylvaticus* | Male | 1960 | -76.7414 | 38.5342 |
| SMNH | 369419 | *Lithobates sylvaticus* | Male | 1960 | -76.7414 | 38.5342 |
| SMNH | 369420 | *Lithobates sylvaticus* | Male | 1960 | -76.7414 | 38.5342 |
| SMNH | 381899 | *Lithobates sylvaticus* | Female | 1963 | -77.4714 | 39.6306 |
| SMNH | 391655 | *Lithobates sylvaticus* | Female | 1970 | -76.5644 | 41.1231 |
| SMNH | 414880 | *Lithobates sylvaticus* | Female | 1960 | -79.5644 | 37.4817 |
| SMNH | 521813 | *Lithobates sylvaticus* | Female | 1965 | -106.1411 | 41.2983 |
| SMNH | 521814 | *Lithobates sylvaticus* | Female | 1965 | -106.1411 | 41.2983 |
| SMNH | 521815 | *Lithobates sylvaticus* | Female | 1965 | -106.1411 | 41.2983 |
| SMNH | 521816 | *Lithobates sylvaticus* | Female | 1965 | -106.1411 | 41.2983 |
| SMNH | 521817 | *Lithobates sylvaticus* | Female | 1965 | -106.1411 | 41.2983 |
| SMNH | 521840 | *Lithobates sylvaticus* | Male | 1965 | -106.1511 | 41.0806 |
| SMNH | 521841 | *Lithobates sylvaticus* | Male | 1965 | -106.1511 | 41.0806 |
| SMNH | 521842 | *Lithobates sylvaticus* | Male | 1965 | -106.1511 | 41.0806 |
| SMNH | 521844 | *Lithobates sylvaticus* | Male | 1965 | -106.1511 | 41.0806 |
| SMNH | 521847 | *Lithobates sylvaticus* | Male | 1965 | -106.1511 | 41.0806 |
| SMNH | 521848 | *Lithobates sylvaticus* | Male | 1965 | -106.1511 | 41.0806 |
| SMNH | 521850 | *Lithobates sylvaticus* | Male | 1965 | -106.1511 | 41.0806 |
| SMNH | 521852 | *Lithobates sylvaticus* | Male | 1965 | -106.1511 | 41.0806 |
| SMNH | 521853 | *Lithobates sylvaticus* | Male | 1965 | -106.1511 | 41.0806 |
| SMNH | 521855 | *Lithobates sylvaticus* | Male | 1965 | -106.1511 | 41.0806 |
| SMNH | 521858 | *Lithobates sylvaticus* | Female | 1965 | -106.1511 | 41.0806 |
| SMNH | 521859 | *Lithobates sylvaticus* | Female | 1965 | -106.1511 | 41.0806 |
| SMNH | 521860 | *Lithobates sylvaticus* | Female | 1968 | -106.1511 | 41.0806 |
| SMNH | 521861 | *Lithobates sylvaticus* | Female | 1968 | -106.1511 | 41.0806 |
| SMNH | 521862 | *Lithobates sylvaticus* | Male | 1968 | -106.1511 | 41.0806 |
| SMNH | 521863 | *Lithobates sylvaticus* | Male | 1968 | -106.1511 | 41.0806 |
| SMNH | 521867 | *Lithobates sylvaticus* | Male | 1965 | -99.3064 | 49.8714 |
| SMNH | 521868 | *Lithobates sylvaticus* | Male | 1965 | -99.3064 | 49.8714 |
| SMNH | 521874 | *Lithobates sylvaticus* | Male | 1965 | -99.3064 | 49.8714 |
| SMNH | 521881 | *Lithobates sylvaticus* | Female | 1965 | -99.3019 | 49.8714 |
| SMNH | 521886 | *Lithobates sylvaticus* | Female | 1965 | -99.3019 | 49.8714 |
| SMNH | 521887 | *Lithobates sylvaticus* | Female | 1965 | -99.3019 | 49.8714 |
| SMNH | 521888 | *Lithobates sylvaticus* | Female | 1965 | -99.3019 | 49.8714 |
| SMNH | 521889 | *Lithobates sylvaticus* | Female | 1965 | -99.3019 | 49.8714 |
| SMNH | 521890 | *Lithobates sylvaticus* | Female | 1965 | -99.3019 | 49.8714 |
| SMNH | 521892 | *Lithobates sylvaticus* | Female | 1967 | -100.0694 | 49.1261 |
| SMNH | 521894 | *Lithobates sylvaticus* | Male | 1967 | -100.0694 | 49.1261 |
| SMNH | 521895 | *Lithobates sylvaticus* | Female | 1967 | -100.0694 | 49.1261 |
| SMNH | 521897 | *Lithobates sylvaticus* | Male | 1967 | -100.0694 | 49.1261 |
| SMNH | 521898 | *Lithobates sylvaticus* | Male | 1967 | -100.0694 | 49.1261 |
| SMNH | 521899 | *Lithobates sylvaticus* | Female | 1967 | -100.0694 | 49.1261 |
| SMNH | 521900 | *Lithobates sylvaticus* | Male | 1967 | -100.0694 | 49.1261 |
| SMNH | 521904 | *Lithobates sylvaticus* | Female | 1967 | -100.0694 | 49.1261 |
| SMNH | 521905 | *Lithobates sylvaticus* | Male | 1967 | -100.0694 | 49.1261 |
| SMNH | 521908 | *Lithobates sylvaticus* | Male | 1967 | -100.0694 | 49.1261 |
| SMNH | 521909 | *Lithobates sylvaticus* | Male | 1967 | -100.0694 | 49.1261 |
| SMNH | 533173 | *Lithobates sylvaticus* | Male | 1943 | -76.8092 | 39.0478 |
| SMNH | 533174 | *Lithobates sylvaticus* | Male | 1943 | -76.8092 | 39.0478 |
| SMNH | 533175 | *Lithobates sylvaticus* | Male | 1943 | -76.8092 | 39.0478 |
| SMNH | 541046 | *Lithobates sylvaticus* | Female | 1984 | -81.6142 | 36.6975 |
| SMNH | 541047 | *Lithobates sylvaticus* | Female | 1984 | -79.8500 | 37.9167 |
| SMNH | 541050 | *Lithobates sylvaticus* | Male | 1985 | -78.9983 | 38.5067 |
| SMNH | 541051 | *Lithobates sylvaticus* | Female | 1985 | -79.0017 | 38.5150 |
| SMNH | 541052 | *Lithobates sylvaticus* | Male | 1985 | -78.9789 | 38.5528 |
| SMNH | 541053 | *Lithobates sylvaticus* | Male | 1985 | -78.9708 | 38.5636 |
| SMNH | 541054 | *Lithobates sylvaticus* | Female | 1985 | -78.9708 | 38.5636 |
| SMNH | 541055 | *Lithobates sylvaticus* | Male | 1985 | -78.9325 | 38.6017 |
| SMNH | 541056 | *Lithobates sylvaticus* | Male | 1985 | -78.9325 | 38.6017 |
| SMNH | 541057 | *Lithobates sylvaticus* | Female | 1985 | -78.9236 | 38.6133 |
| SMNH | 541059 | *Lithobates sylvaticus* | Male | 1980 | -78.9667 | 37.5500 |
| SMNH | 541060 | *Lithobates sylvaticus* | Male | 1980 | -78.9667 | 37.5500 |
| SMNH | 541061 | *Lithobates sylvaticus* | Male | 1980 | -78.9667 | 37.5500 |
| SMNH | 541062 | *Lithobates sylvaticus* | Male | 1980 | -78.9667 | 37.5500 |
| SMNH | 541063 | *Lithobates sylvaticus* | Male | 1980 | -79.7453 | 36.8111 |
| SMNH | 541065 | *Lithobates sylvaticus* | Male | 1983 | -78.1667 | 37.7833 |
| SMNH | 541066 | *Lithobates sylvaticus* | Male | 1983 | -78.1667 | 37.7833 |
| SMNH | 541067 | *Lithobates sylvaticus* | Female | 1983 | -82.9528 | 36.7933 |
| SMNH | 541068 | *Lithobates sylvaticus* | Male | 1983 | -82.9425 | 36.7964 |
| SMNH | 541159 | *Lithobates sylvaticus* | Female | 1987 | -79.2703 | 38.4333 |
| SMNH | 541170 | *Lithobates sylvaticus* | Female | 1987 | -79.0861 | 38.6667 |
| SMNH | 541182 | *Lithobates sylvaticus* | Female | 1987 | -79.2942 | 38.4164 |
| SMNH | 541186 | *Lithobates sylvaticus* | Female | 1987 | -79.2942 | 38.4164 |
| SMNH | 541188 | *Lithobates sylvaticus* | Female | 1987 | -79.2942 | 38.4164 |
| SMNH | 541203 | *Lithobates sylvaticus* | Female | 1987 | -79.0797 | 38.6231 |
| SMNH | 541205 | *Lithobates sylvaticus* | Female | 1987 | -79.0797 | 38.6231 |
| SMNH | 541227 | *Lithobates sylvaticus* | Female | 1987 | -79.0797 | 38.6231 |
| SMNH | 543519 | *Lithobates sylvaticus* | Female | 1956 | -93.5269 | 47.2350 |
| SMNH | 543520 | *Lithobates sylvaticus* | Female | 1956 | -93.5269 | 47.2350 |
| SMNH | 543523 | *Lithobates sylvaticus* | Female | 1947 | -100.2964 | 48.9258 |
| UA | A1170 | *Lithobates sylvaticus* | Male | 1943 | -113.4667 | 53.5500 |
| UA | A1171 | *Lithobates sylvaticus* | Male | 1943 | -113.4667 | 53.5500 |
| UA | A1173 | *Lithobates sylvaticus* | Male | 1943 | -113.4667 | 53.5500 |
| UA | A1174 | *Lithobates sylvaticus* | Male | 1943 | -113.4667 | 53.5500 |
| UA | A1175 | *Lithobates sylvaticus* | Male | 1943 | -113.4667 | 53.5500 |
| UA | A1176 | *Lithobates sylvaticus* | Male | 1943 | -113.4667 | 53.5500 |
| UA | A1177 | *Lithobates sylvaticus* | Male | 1943 | -113.4667 | 53.5500 |
| UA | A1178 | *Lithobates sylvaticus* | Male | 1943 | -113.4667 | 53.5500 |
| UA | A1179 | *Lithobates sylvaticus* | Male | 1943 | -113.4667 | 53.5500 |
| UA | A1229 | *Lithobates sylvaticus* | Female | 1961 | -112.5333 | 53.4500 |
| UA | A1231 | *Lithobates sylvaticus* | Female | 1961 | -112.5333 | 53.4500 |
| UA | A1232 | *Lithobates sylvaticus* | Male | 1954 | -118.8667 | 55.0500 |
| UA | A1234 | *Lithobates sylvaticus* | Female | 1954 | -118.8667 | 55.0500 |
| UA | A1236 | *Lithobates sylvaticus* | Female | 1943 | -113.4667 | 53.5500 |
| UA | A1237 | *Lithobates sylvaticus* | Male | 1943 | -113.4667 | 53.5500 |
| UA | A1357 | *Lithobates sylvaticus* | Male | 1963 | -115.1333 | 53.9500 |
| UA | A1372 | *Lithobates sylvaticus* | Male | 1963 | -117.7333 | 56.2333 |
| UA | A1377 | *Lithobates sylvaticus* | Male | 1962 | -113.0167 | 53.3500 |
| UA | A1377-a | *Lithobates sylvaticus* | Male | 1962 | -113.0167 | 53.3500 |
| UA | A1446 | *Lithobates sylvaticus* | Female | 1960 | -113.4931 | 53.6722 |
| UA | A1447 | *Lithobates sylvaticus* | Male | 1961 | -112.5333 | 53.4500 |
| UA | A1448 | *Lithobates sylvaticus* | Male | 1960 | -115.8456 | 53.6069 |
| UA | A1450 | *Lithobates sylvaticus* | Male | 1960 | -115.8456 | 53.6069 |
| UA | A1457 | *Lithobates sylvaticus* | Male | 1960 | -115.8456 | 53.6069 |
| UA | A1457-b | *Lithobates sylvaticus* | Male | 1960 | -115.8456 | 53.6069 |
| UA | A1457-c | *Lithobates sylvaticus* | Male | 1960 | -115.8456 | 53.6069 |
| UA | A1457-d | *Lithobates sylvaticus* | Male | 1960 | -115.8456 | 53.6069 |
| UA | A1462 | *Lithobates sylvaticus* | Female | 1961 | -117.1042 | 55.5486 |
| UA | A1524 | *Lithobates sylvaticus* | Female | 1966 | -114.6500 | 50.6500 |
| UA | A1525 | *Lithobates sylvaticus* | Female | 1966 | -114.6500 | 50.6500 |
| UA | A1780 | *Lithobates sylvaticus* | Male | 1968 | -114.6500 | 50.6500 |
| UA | A1858 | *Lithobates sylvaticus* | Female | 1968 | -114.6500 | 50.6500 |
| UA | A1859 | *Lithobates sylvaticus* | Female | 1968 | -114.6500 | 50.6500 |
| UA | A189 | *Lithobates sylvaticus* | Male | 1950 | -114.5833 | 53.5333 |
| UA | A192 | *Lithobates sylvaticus* | Female | 1950 | -114.5833 | 53.5333 |
| UA | A1987 | *Lithobates sylvaticus* | Female | 1970 | -111.8333 | 54.7167 |
| UA | A1988 | *Lithobates sylvaticus* | Male | 1970 | -113.2167 | 53.4764 |
| UA | A2014 | *Lithobates sylvaticus* | Male | 1971 | -119.1681 | 53.8931 |
| UA | A2087 | *Lithobates sylvaticus* | Male | 1971 | -119.0833 | 53.8833 |
| UA | A2128 | *Lithobates sylvaticus* | Male | 1972 | -113.7875 | 53.5639 |
| UA | A2215 | *Lithobates sylvaticus* | Female | 1975 | -117.3667 | 60.8667 |
| UA | A2229 | *Lithobates sylvaticus* | Male | 1976 | -111.3803 | 56.7264 |
| UA | A2230 | *Lithobates sylvaticus* | Male | 1976 | -116.4261 | 53.5222 |
| UA | A2231 | *Lithobates sylvaticus* | Female | 1976 | -116.4261 | 53.5222 |
| UA | A2247 | *Lithobates sylvaticus* | Male | 1976 | -116.2500 | 54.2500 |
| UA | A2248 | *Lithobates sylvaticus* | Male | 1976 | -116.2500 | 54.2500 |
| UA | A2249 | *Lithobates sylvaticus* | Male | 1976 | -116.2500 | 54.2500 |
| UA | A2250 | *Lithobates sylvaticus* | Male | 1976 | -116.2500 | 54.2500 |
| UA | A2251 | *Lithobates sylvaticus* | Female | 1976 | -116.2500 | 54.2500 |
| UA | A2263 | *Lithobates sylvaticus* | Male | 1976 | -117.4806 | 56.1306 |
| UA | A2265 | *Lithobates sylvaticus* | Male | 1976 | -114.3178 | 53.0542 |
| UA | A2280 | *Lithobates sylvaticus* | Male | 1976 | -112.5403 | 53.2153 |
| UA | A2281 | *Lithobates sylvaticus* | Male | 1976 | -113.6889 | 53.3778 |
| UA | A2283 | *Lithobates sylvaticus* | Male | 1976 | -114.0333 | 53.0167 |
| UA | A2284 | *Lithobates sylvaticus* | Male | 1976 | -114.0333 | 53.0167 |
| UA | A2299 | *Lithobates sylvaticus* | Male | 1976 | -118.6500 | 55.1694 |
| UA | A2348 | *Lithobates sylvaticus* | Male | 1976 | -115.1667 | 50.6500 |
| UA | A2349 | *Lithobates sylvaticus* | Female | 1976 | -115.1667 | 50.6500 |
| UA | A2367 | *Lithobates sylvaticus* | Male | 1976 | -115.1667 | 50.6500 |
| UA | A2396 | *Lithobates sylvaticus* | Male | 1976 | -115.1111 | 50.7125 |
| UA | A2397 | *Lithobates sylvaticus* | Male | 1976 | -115.1111 | 50.7125 |
| UA | A2421 | *Lithobates sylvaticus* | Male | 1976 | -112.3164 | 55.0708 |
| UA | A2422 | *Lithobates sylvaticus* | Male | 1976 | -112.3164 | 55.0708 |
| UA | A2445 | *Lithobates sylvaticus* | Female | 1976 | -117.9064 | 56.3047 |
| UA | A2462 | *Lithobates sylvaticus* | Male | 1973 | -115.2833 | 55.0000 |
| UA | A2462-a | *Lithobates sylvaticus* | Female | 1973 | -115.2833 | 55.0000 |
| UA | A2465 | *Lithobates sylvaticus* | Female | 1973 | -115.3000 | 55.4333 |
| UA | A2466 | *Lithobates sylvaticus* | Male | 1973 | -115.3000 | 55.4333 |
| UA | A2467 | *Lithobates sylvaticus* | Male | 1973 | -115.3000 | 55.4333 |
| UA | A2475 | *Lithobates sylvaticus* | Male | 1977 | -112.5403 | 53.0875 |
| UA | A2476 | *Lithobates sylvaticus* | Male | 1977 | -112.5403 | 53.0875 |
| UA | A2478 | *Lithobates sylvaticus* | Male | 1977 | -114.2486 | 53.5694 |
| UA | A2512 | *Lithobates sylvaticus* | Female | 1977 | -118.5014 | 59.6583 |
| UA | A2527 | *Lithobates sylvaticus* | Male | 1978 | -114.1250 | 53.7014 |
| UA | A2533 | *Lithobates sylvaticus* | Female | 1978 | -112.6167 | 54.5833 |
| UA | A2534 | *Lithobates sylvaticus* | Male | 1978 | -112.8789 | 54.5875 |
| UA | A2542 | *Lithobates sylvaticus* | Female | 1978 | -115.6775 | 54.4347 |
| UA | A2565 | *Lithobates sylvaticus* | Female | 1979 | -114.1736 | 53.5708 |
| UA | A2595 | *Lithobates sylvaticus* | Male | 1979 | -110.0286 | 54.4486 |
| UA | A2671 | *Lithobates sylvaticus* | Male | 1976 | -111.2833 | 56.5125 |
| UA | A2680 | *Lithobates sylvaticus* | Female | 1976 | -111.0000 | 56.4167 |
| UA | A2692 | *Lithobates sylvaticus* | Female | 1976 | -111.6000 | 56.2792 |
| UA | A2697 | *Lithobates sylvaticus* | Female | 1976 | -111.6653 | 57.2542 |
| UA | A27 | *Lithobates sylvaticus* | Female | 1947 | -118.6167 | 54.5500 |
| UA | A2705 | *Lithobates sylvaticus* | Female | 1976 | -111.1125 | 56.4458 |
| UA | A2706 | *Lithobates sylvaticus* | Female | 1976 | -111.1125 | 56.4458 |
| UA | A2710 | *Lithobates sylvaticus* | Male | 1976 | -111.5958 | 56.3167 |
| UA | A2712 | *Lithobates sylvaticus* | Female | 1976 | -111.6000 | 56.3333 |
| UA | A2713 | *Lithobates sylvaticus* | Female | 1976 | -111.6000 | 56.3333 |
| UA | A2718 | *Lithobates sylvaticus* | Female | 1976 | -111.2500 | 56.5333 |
| UA | A2720 | *Lithobates sylvaticus* | Male | 1976 | -111.6000 | 56.2750 |
| UA | A2725 | *Lithobates sylvaticus* | Female | 1976 | -111.6000 | 57.1000 |
| UA | A2731 | *Lithobates sylvaticus* | Male | 1976 | -111.6653 | 57.2542 |
| UA | A2740 | *Lithobates sylvaticus* | Female | 1976 | -111.6000 | 57.1000 |
| UA | A2746 | *Lithobates sylvaticus* | Female | 1976 | -111.4667 | 56.9625 |
| UA | A2748 | *Lithobates sylvaticus* | Female | 1976 | -111.6333 | 56.7500 |
| UA | A2750 | *Lithobates sylvaticus* | Female | 1976 | -111.6000 | 56.3333 |
| UA | A2753 | *Lithobates sylvaticus* | Male | 1976 | -111.2500 | 56.5333 |
| UA | A2764 | *Lithobates sylvaticus* | Female | 1976 | -111.6000 | 57.1667 |
| UA | A2765 | *Lithobates sylvaticus* | Female | 1976 | -111.6000 | 57.1667 |
| UA | A2769 | *Lithobates sylvaticus* | Female | 1976 | -111.3000 | 56.5833 |
| UA | A2771 | *Lithobates sylvaticus* | Male | 1976 | -111.2500 | 56.5333 |
| UA | A2772 | *Lithobates sylvaticus* | Female | 1976 | -111.0500 | 56.4500 |
| UA | A30 | *Lithobates sylvaticus* | Female | 1939 | -114.5833 | 53.5333 |
| UA | A3040 | *Lithobates sylvaticus* | Female | 1996 | -113.3947 | 52.0283 |
| UA | A312 | *Lithobates sylvaticus* | Female | 1950 | -114.0000 | 49.9167 |
| UA | A314 | *Lithobates sylvaticus* | Male | 1950 | -113.3667 | 49.7667 |
| UA | A32 | *Lithobates sylvaticus* | Male | 1943 | -113.4667 | 53.5500 |
| UA | A34 | *Lithobates sylvaticus* | Male | 1943 | -113.4667 | 53.5500 |
| UA | A35 | *Lithobates sylvaticus* | Female | 1943 | -113.4667 | 53.5500 |
| UA | A36 | *Lithobates sylvaticus* | Male | 1943 | -113.4667 | 53.5500 |
| UA | A3668 | *Lithobates sylvaticus* | Male | 1981 | -114.4000 | 52.9500 |
| UA | A37 | *Lithobates sylvaticus* | Male | 1943 | -113.4667 | 53.5500 |
| UA | A38 | *Lithobates sylvaticus* | Male | 1943 | -113.4667 | 53.5500 |
| UA | A39 | *Lithobates sylvaticus* | Male | 1943 | -113.4667 | 53.5500 |
| UA | A40 | *Lithobates sylvaticus* | Male | 1943 | -113.4667 | 53.5500 |
| UA | A403 | *Lithobates sylvaticus* | Female | 1951 | -112.7167 | 52.3167 |
| UA | A42 | *Lithobates sylvaticus* | Male | 1949 | -111.8333 | 54.9000 |
| UA | A44 | *Lithobates sylvaticus* | Male | 1949 | -111.8333 | 54.9000 |
| UA | A446 | *Lithobates sylvaticus* | Female | 1951 | -115.6667 | 61.6000 |
| UA | A447 | *Lithobates sylvaticus* | Female | 1951 | -115.6667 | 61.6000 |
| UA | A448 | *Lithobates sylvaticus* | Female | 1951 | -115.7972 | 60.8153 |
| UA | A450 | *Lithobates sylvaticus* | Female | 1951 | -115.7972 | 60.8153 |
| UA | A537 | *Lithobates sylvaticus* | Male | 1952 | -118.1000 | 52.9833 |
| UA | A538 | *Lithobates sylvaticus* | Female | 1952 | -118.1000 | 52.9833 |
| UA | A545 | *Lithobates sylvaticus* | Male | 1952 | -118.1000 | 52.9833 |
| UA | A546 | *Lithobates sylvaticus* | Male | 1952 | -116.0500 | 51.6000 |
| UA | A547 | *Lithobates sylvaticus* | Female | 1952 | -116.0500 | 51.6000 |
| UA | A548 | *Lithobates sylvaticus* | Female | 1952 | -116.0500 | 51.6000 |
| UA | A566 | *Lithobates sylvaticus* | Female | 1952 | -114.6858 | 50.9486 |
| UA | A567 | *Lithobates sylvaticus* | Female | 1952 | -114.6858 | 50.9486 |
| UA | A598 | *Lithobates sylvaticus* | Female | 1952 | -110.9167 | 55.6333 |
| UA | A599 | *Lithobates sylvaticus* | Male | 1952 | -111.8333 | 54.9000 |
| UA | A601 | *Lithobates sylvaticus* | Male | 1952 | -111.8333 | 54.9000 |
| UA | A613 | *Lithobates sylvaticus* | Female | 1952 | -116.0833 | 52.4667 |
| UA | A614 | *Lithobates sylvaticus* | Female | 1952 | -116.0833 | 52.4667 |
| UA | A615 | *Lithobates sylvaticus* | Female | 1952 | -116.0833 | 52.4667 |
| UA | A616 | *Lithobates sylvaticus* | Female | 1952 | -116.0833 | 52.4667 |
| UA | A617 | *Lithobates sylvaticus* | Male | 1952 | -116.0833 | 52.4667 |
| UA | A618 | *Lithobates sylvaticus* | Male | 1952 | -116.0833 | 52.4667 |
| UA | A622 | *Lithobates sylvaticus* | Male | 1952 | -118.2500 | 53.4500 |
| UA | A623 | *Lithobates sylvaticus* | Male | 1952 | -118.2500 | 53.4500 |
| UA | A626 | *Lithobates sylvaticus* | Male | 1952 | -118.2500 | 53.4500 |
| UA | A629 | *Lithobates sylvaticus* | Female | 1952 | -118.2500 | 53.4500 |
| UA | A632 | *Lithobates sylvaticus* | Female | 1952 | -118.2500 | 53.4500 |
| UA | A634 | *Lithobates sylvaticus* | Female | 1953 | -113.2000 | 51.9833 |
| UA | A635 | *Lithobates sylvaticus* | Female | 1953 | -113.1667 | 52.1167 |
| UA | A636 | *Lithobates sylvaticus* | Female | 1953 | -113.1667 | 52.1167 |
| UA | A637 | *Lithobates sylvaticus* | Female | 1953 | -113.2333 | 52.2000 |
| UA | A638 | *Lithobates sylvaticus* | Female | 1953 | -113.2333 | 52.2000 |
| UA | A666 | *Lithobates sylvaticus* | Male | 1953 | -114.7333 | 53.5500 |
| UA | A691 | *Lithobates sylvaticus* | Female | 1954 | -111.9667 | 54.7667 |
| UA | A694 | *Lithobates sylvaticus* | Male | 1952 | -118.2500 | 53.4500 |
| UA | A697 | *Lithobates sylvaticus* | Male | 1954 | -117.0333 | 59.8667 |
| UA | A699 | *Lithobates sylvaticus* | Female | 1954 | -117.0333 | 59.8667 |
| UA | A700 | *Lithobates sylvaticus* | Female | 1954 | -117.0333 | 59.8667 |
| UA | A704 | *Lithobates sylvaticus* | Female | 1954 | -118.6000 | 56.2500 |
| UA | A706 | *Lithobates sylvaticus* | Female | 1954 | -118.8667 | 55.0500 |
| UA | A707 | *Lithobates sylvaticus* | Female | 1954 | -118.8667 | 55.0500 |
| UA | A708 | *Lithobates sylvaticus* | Female | 1954 | -118.8667 | 55.0500 |
| UA | A709 | *Lithobates sylvaticus* | Male | 1954 | -118.8667 | 55.0500 |
| UA | A734 | *Lithobates sylvaticus* | Female | 1954 | -114.1667 | 53.5000 |
| UA | A735 | *Lithobates sylvaticus* | Male | 1954 | -114.1667 | 53.5000 |
| UA | A777 | *Lithobates sylvaticus* | Female | 1956 | -115.4000 | 54.7167 |
| UA | A778 | *Lithobates sylvaticus* | Female | 1956 | -115.4000 | 54.7167 |
| UA | A779 | *Lithobates sylvaticus* | Female | 1956 | -115.4000 | 54.7167 |
| UA | A780 | *Lithobates sylvaticus* | Female | 1956 | -115.4000 | 54.7167 |
| UA | A781 | *Lithobates sylvaticus* | Male | 1956 | -115.4000 | 54.7167 |
| UA | A800 | *Lithobates sylvaticus* | Female | 1957 | -126.8292 | 65.2819 |
| UA | A99 | *Lithobates sylvaticus* | Female | 1950 | -114.4167 | 53.7000 |
| YPM | 1020 | *Lithobates sylvaticus* | Female | 1954 | -72.9975 | 41.4217 |
| YPM | 2874 | *Lithobates sylvaticus* | Female | 1935 | -72.9722 | 41.3306 |
| YPM | 2875 | *Lithobates sylvaticus* | Male | 1935 | -72.9722 | 41.3306 |
| YPM | 2876 | *Lithobates sylvaticus* | Male | 1935 | -72.9722 | 41.3306 |
| YPM | 2877 | *Lithobates sylvaticus* | Male | 1935 | -72.9722 | 41.3306 |
| YPM | 2881 | *Lithobates sylvaticus* | Male | 1935 | -72.9722 | 41.3306 |
| YPM | 2885 | *Lithobates sylvaticus* | Male | 1935 | -72.9722 | 41.3306 |
| YPM | 2887 | *Lithobates sylvaticus* | Male | 1935 | -72.9722 | 41.3306 |
| YPM | 2888 | *Lithobates sylvaticus* | Male | 1935 | -72.9722 | 41.3306 |
| YPM | 2889 | *Lithobates sylvaticus* | Male | 1935 | -72.9722 | 41.3306 |
| YPM | 2890 | *Lithobates sylvaticus* | Male | 1935 | -72.9722 | 41.3306 |
| YPM | 2892 | *Lithobates sylvaticus* | Male | 1935 | -72.9722 | 41.3306 |
| YPM | 2894 | *Lithobates sylvaticus* | Male | 1935 | -72.9722 | 41.3306 |
| YPM | 2901 | *Lithobates sylvaticus* | Male | 1935 | -72.9722 | 41.3306 |
| YPM | 2902 | *Lithobates sylvaticus* | Female | 1935 | -72.9722 | 41.3306 |
| YPM | 3204 | *Lithobates sylvaticus* | Female | 1932 | -73.0089 | 41.3525 |
| YPM | 3207 | *Lithobates sylvaticus* | Female | 1932 | -73.0089 | 41.3525 |
| YPM | 3219 | *Lithobates sylvaticus* | Male | 1932 | -73.0089 | 41.3525 |
| YPM | 3224 | *Lithobates sylvaticus* | Male | 1932 | -73.0089 | 41.3525 |
| YPM | 3230 | *Lithobates sylvaticus* | Female | 1932 | -73.0089 | 41.3525 |
| YPM | 3239 | *Lithobates sylvaticus* | Male | 1932 | -73.0089 | 41.3525 |
| YPM | 3698 | *Lithobates sylvaticus* | Male | 1969 | -72.4128 | 41.9611 |
| YPM | 3699 | *Lithobates sylvaticus* | Male | 1969 | -72.4128 | 41.9611 |
| YPM | 3700 | *Lithobates sylvaticus* | Male | 1969 | -72.4128 | 41.9611 |
| YPM | 3701 | *Lithobates sylvaticus* | Female | 1969 | -72.4128 | 41.9611 |
| YPM | 4179 | *Lithobates sylvaticus* | Male | 1969 | -72.8942 | 41.8244 |

**Table S2.** Sample sizes available for body size and Julian day of first collection (breeding) across decades. Note that sample sizes for Julian day do not equal the sum of males and females due to the removal of individuals appearing outside of the breeding season from this latter data set (see Methods), and the fact that individuals were omitted from JD dataset if exact collection date was not available, while for male & female body size, only collection year was necessary.

| Decade | Females | Males | Julian Day |
| --- | --- | --- | --- |
| 1900 | 21 | 30 | 29 |
| 1910 | 6 | 3 | 6 |
| 1920 | 15 | 12 | 19 |
| 1930 | 52 | 71 | 68 |
| 1940 | 68 | 181 | 121 |
| 1950 | 99 | 102 | 116 |
| 1960 | 66 | 123 | 143 |
| 1970 | 79 | 88 | 98 |
| 1980 | 44 | 114 | 111 |
| 1990 | 31 | 55 | 61 |
| 2000 | 3 | 1 | 1 |

**Table S3.** Variance inflation factors (VIF) for predictors (frost free day, precipitation, temperature) of body size and Julian day of first capture (a proxy for breeding).

| Response | VIF | | | | | | |
| --- | --- | --- | --- | --- | --- | --- | --- |
|  | FFD | Precipitation | Temperature | FFD:Precip | FFD:Temp | Precip:Temp | Precip:Temp:FFD |
| Female Body Size | 37.051 | 5.256 | 51.642 | 26.643 | 2.834 | 36.536 | 9.386 |
|  | 36.363 | 4.700 | 51.617 | 24.063 | 2.803 | 27.093 |  |
|  | 16.716 | 3.601 | 21.467 | 3.319 | 2.796 |  |  |
|  | 16.687 | 3.233 | 20.711 | 2.041 |  |  |  |
|  | 2.129 | 3.022 |  | 1.815 |  |  |  |
| Male Body Size | 12.945 | 2.898 | 15.058 | 17.900 | 1.769 | 20.041 | 2.496 |
|  | 12.043 | 1.291 | 13.514 | 17.120 | 1.690 | 18.965 |  |
|  | 4.893 | 1.282 | 4.196 | 1.247 | 1.367 |  |  |
|  | 4.022 | 1.282 | 3.999 | 1.257 |  |  |  |
|  | 1.007 | 1.257 |  | 1.257 |  |  |  |
| Julian day | 13.981 | 3.298 | 17.043 | 21.914 | 1.883 | 23.985 | 2.995 |
|  | 13.573 | 1.269 | 16.319 | 21.778 | 1.796 | 23.297 |  |
|  | 7.017 | 1.238 | 6.674 | 1.087 | 1.640 |  |  |
|  | 6.091 | 1.180 | 6.520 | 1.060 |  |  |  |
|  | 1.097 | 1.039 |  | 1.057 |  |  |  |

**Table S4.** Models testing best fit between climate and size or Julian day of first collection, for data from 1901–1960. Models in bold text (ΔAICc < 2) are those which received sufficient support to be included in further analyses.

|  | **Model** | **AICc** | **ΔAICc** | **R^2^m** | **R^2^c** |
| --- | --- | --- | --- | --- | --- |
| Female Body Size | **FFD*Precipitation** | **-494.44** | **0.00** | **0.429** | **0.429** |
|  | Precipitation | -483.21 | 11.23 | 0.396 | 0.423 |
|  | FFD + Precipitation | -481.15 | 13.29 | 0.396 | 0.425 |
|  | FFD | -430.32 | 64.12 | 0.195 | 0.282 |
|  | Intercept Only | -394.65 | 99.79 | 0.000 | 0.222 |
| Male Body Size | **FFD + Precipitation** | **-782.77** | **0.00** | **0.092** | **0.191** |
|  | **FFD** | **-782.37** | **0.40** | **0.087** | **0.205** |
|  | **FFD*Precipitation** | **-781.11** | **1.66** | **0.095** | **0.190** |
|  | Intercept Only | -762.77 | 19.99 | 0.000 | 0.184 |
|  | Precipitation | -761.74 | 21.03 | 0.001 | 0.173 |
| Julian day of first collection | **FFD*Precipitation** | **3145.63** | **0.00** | **0.648** | **0.719** |
|  | **FFD** | **3147.19** | **1.56** | **0.643** | **0.714** |
|  | Intercept Only | 3293.18 | 147.55 | 0.000 | 0.210 |
|  | FFD + Precipitation | 3446.26 | 300.63 | 0.628 | 0.830 |
|  | Precipitation | 3857.42 | 711.79 | 0.091 | 0.427 |


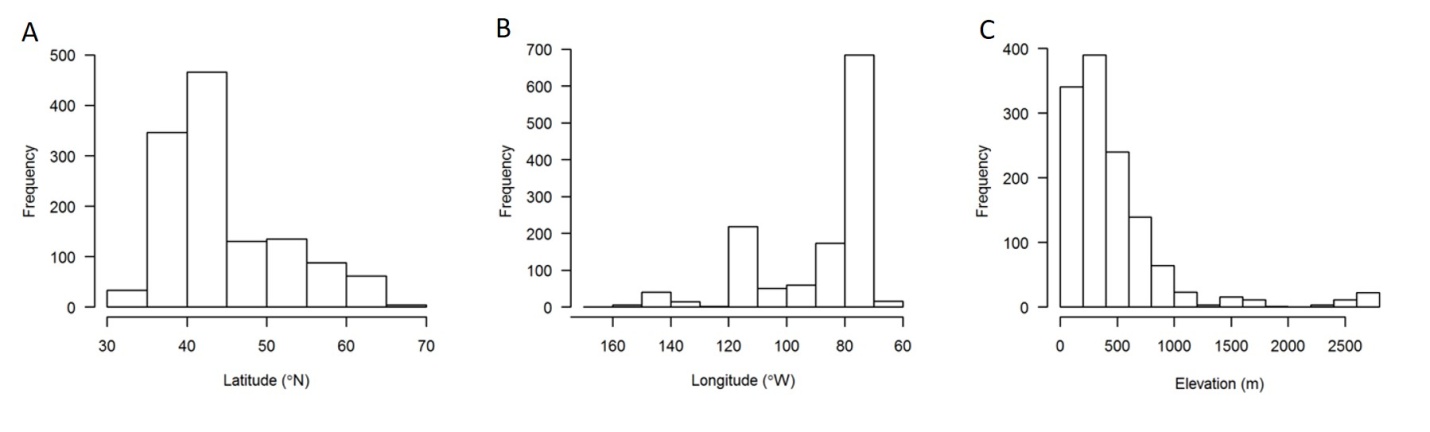


**Figure S1.** Sample sizes for each set of A) latitudes, B) longitudes, and C) elevations.

**Table S5.** Models of historical (1901–1960) relationship between climate variables and female body size, male body size, and Julian day of first collection (breeding). Note that χ2 and P-values are only possible when there is only one best model based on ΔAICc < 2 (Table S3).

|  | Fixed Effect | Estimate | Lower 95% CI | Upper 95% CI | χ2 | P |
| --- | --- | --- | --- | --- | --- | --- |
| Females | FFD | -0.003 | -0.024 | 0.018 | 0.03 | 0.85 |
|  | **Precipitation** | **0.112** | **0.088** | **0.136** | **52.93** | **<0.001** |
|  | **FFD:Precipitation** | **0.045** | **0.023** | **0.067** | **15.40** | **<0.001** |
| Males | **FFD** | **0.041** | **0.024** | **0.057** | **n/a** | **n/a** |
|  | Precipitation | 0.008 | -0.002 | 0.018 | n/a | n/a |
|  | FFD:Precipitation | -0.004 | -0.014 | 0.007 | n/a | n/a |
| Julian Day | **FFD** | **-42.403** | **-47.611** | **-37.195** | **n/a** | **n/a** |
|  | **Precipitation** | **-1.219** | **-2.260** | **-0.179** | **n/a** | **n/a** |
|  | FFD:Precipitation | .377 | -0.736 | 1.490 | n/a | n/a |

**Appendix S1. Effect of cut-off date choice on estimates of change in size and breeding.**

*Sensitivity of historical relationship between climate and both size and breeding (Julian day) to cut-off date.*

To confirm our choice of cut-off date, we tested whether the relationship between climate variables and body size (female or male) or collection date varied with choice of cut-off date, comparing our model selection (Tables A1-A3) and coefficient values with 95% CI for 8 different cut-off years (every 5 years from 1940–1980), and compared decadal coefficient estimates (1930, 1940, 1950, 1960, 1970, 1980) to our 1960 cut-off date estimates. We found no effect of choice of cut-off date on our results, and no consistent trend in observations when compared on a decade by decade basis.

Table A1. AICc for female body size and historical climate data.

| **Cut-off (n)** | **Model** | **AICc** | **ΔAICc** | **R^2^m** | **R^2^c** |
| --- | --- | --- | --- | --- | --- |
| 1940 (94) | Precipitation | -146.17 | 0.00 | 0.434 | 0.440 |
|  | FFD*Precipitation | -145.52 | 0.65 | 0.446 | 0.446 |
|  | FFD + Precipitation | -143.90 | 2.27 | 0.434 | 0.442 |
|  | FFD | -124.49 | 21.69 | 0.149 | 0.365 |
|  | Intercept Only | -116.54 | 29.63 | 0.000 | 0.327 |
| 1945 (132) | Precipitation | -222.74 | 0.00 | 0.388 | 0.429 |
|  | FFD*Precipitation | -222.34 | 0.41 | 0.400 | 0.400 |
|  | FFD + Precipitation | -220.60 | 2.15 | 0.389 | 0.430 |
|  | FFD | -194.94 | 27.80 | 0.144 | 0.301 |
|  | Intercept Only | -183.82 | 38.93 | 0.000 | 0.273 |
| 1950 (162) | FFD*Precipitation | -278.58 | 0.00 | 0.385 | 0.402 |
|  | Precipitation | -275.21 | 3.37 | 0.355 | 0.400 |
|  | FFD + Precipitation | -273.95 | 4.63 | 0.360 | 0.410 |
|  | FFD | -240.70 | 37.88 | 0.108 | 0.255 |
|  | Intercept Only | -230.30 | 48.27 | 0.000 | 0.272 |
| 1955 (227) | FFD*Precipitation | -428.32 | 0.00 | 0.429 | 0.429 |
|  | Precipitation | -420.34 | 7.98 | 0.403 | 0.421 |
|  | FFD + Precipitation | -418.52 | 9.79 | 0.405 | 0.428 |
|  | FFD | -369.62 | 58.69 | 0.177 | 0.269 |
|  | Intercept Only | -341.91 | 86.40 | 0.000 | 0.207 |
| 1960 (265) | FFD*Precipitation | -494.44 | 0.00 | 0.429 | 0.429 |
|  | Precipitation | -483.21 | 11.23 | 0.396 | 0.423 |
|  | FFD + Precipitation | -481.15 | 13.29 | 0.396 | 0.425 |
|  | FFD | -430.32 | 64.12 | 0.195 | 0.282 |
|  | Intercept Only | -394.65 | 99.79 | 0.000 | 0.222 |
| 1965 (276) | FFD*Precipitation | -512.34 | 0.00 | 0.430 | 0.430 |
|  | Precipitation | -499.51 | 12.83 | 0.397 | 0.408 |
|  | FFD + Precipitation | -497.44 | 14.90 | 0.397 | 0.409 |
|  | FFD | -444.91 | 67.43 | 0.204 | 0.273 |
|  | Intercept Only | -405.24 | 107.10 | 0.000 | 0.192 |
| 1970 (327) | FFD*Precipitation | -606.14 | 0.00 | 0.395 | 0.395 |
|  | Precipitation | -592.66 | 13.47 | 0.364 | 0.380 |
|  | FFD + Precipitation | -591.58 | 14.56 | 0.367 | 0.386 |
|  | FFD | -530.97 | 75.17 | 0.163 | 0.247 |
|  | Intercept Only | -494.34 | 111.80 | 0.000 | 0.181 |
| 1975 (350) | FFD*Precipitation | -643.96 | 0.00 | 0.420 | 0.420 |
|  | Precipitation | -626.53 | 17.42 | 0.389 | 0.402 |
|  | FFD + Precipitation | -625.57 | 18.39 | 0.391 | 0.411 |
|  | FFD | -549.67 | 94.29 | 0.177 | 0.233 |
|  | Intercept Only | -506.87 | 137.09 | 0.000 | 0.163 |
| 1980 (406) | FFD*Precipitation | -743.55 | 0.00 | 0.435 | 0.477 |
|  | Precipitation | -432.39 | 11.16 | 0.412 | 0.474 |
|  | FFD + Precipitation | -730.71 | 12.84 | 0.412 | 0.478 |
|  | FFD | -645.94 | 97.61 | 0.215 | 0.315 |
|  | Intercept Only | -582.13 | 161.42 | 0.000 | 0.243 |

Table A2. AICc for male body size and historical climate data.

| Cut-off (n) | Model | AICc | ΔAICc | R^2^m | R^2^c |
| --- | --- | --- | --- | --- | --- |
| 1940 (116) | FFD + Precipitation | -200.73 | 0.00 | 0.031 | 0.260 |
|  | FFD*Precipitation | -198.69 | 2.04 | 0.113 | 0.234 |
|  | Precipitation | -194.11 | 6.62 | 0.110 | 0.235 |
|  | FFD | -193.59 | 7.14 | 0.000 | 0.282 |
|  | Intercept Only | -188.34 | 12.40 | 0.081 | 0.240 |
| 1945 (235) | FFD + Precipitation | -456.45 | 0.00 | 0.085 | 0.396 |
|  | FFD*Precipitation | -454.46 | 1.99 | 0.084 | 0.404 |
|  | FFD | -450.10 | 6.35 | 0.064 | 0.422 |
|  | Precipitation | -443.50 | 12.95 | 0.016 | 0.387 |
|  | Intercept Only | -440.58 | 15.87 | 0.000 | 0.393 |
| 1950 (297) | FFD + Precipitation | -556.98 | 0.00 | 0.047 | 0.267 |
|  | FFD*Precipitation | -554.90 | 2.08 | 0.047 | 0.272 |
|  | FFD | -553.80 | 3.17 | 0.040 | 0.277 |
|  | Precipitation | -550.09 | 6.89 | 0.009 | 0.284 |
|  | Intercept Only | -548.46 | 8.52 | 0.000 | 0.291 |
| 1955 (369) | FFD + Precipitation | -690.40 | 0.00 | 0.090 | 0.173 |
|  | FFD*Precipitation | -689.68 | 0.72 | 0.090 | 0.176 |
|  | FFD | -688.22 | 2.18 | 0.085 | 0.186 |
|  | Intercept Only | -670.28 | 20.12 | 0.000 | 0.156 |
|  | Precipitation | -670.11 | 20.29 | 0.003 | 0.146 |
| 1960 (414) | FFD + Precipitation | -782.77 | 0.00 | 0.092 | 0.191 |
|  | FFD | -782.37 | 0.40 | 0.087 | 0.205 |
|  | FFD*Precipitation | -781.11 | 1.66 | 0.095 | 0.190 |
|  | Intercept Only | -762.77 | 19.99 | 0.000 | 0.184 |
|  | Precipitation | -761.74 | 21.03 | 0.001 | 0.173 |
| 1965 (430) | FFD + Precipitation | -807.02 | 0.00 | 0.119 | 0.177 |
|  | FFD | -806.30 | 0.72 | 0.114 | 0.188 |
|  | FFD*Precipitation | -805.36 | 1.66 | 0.121 | 0.178 |
|  | Intercept Only | -779.01 | 28.01 | 0.000 | 0.135 |
|  | Precipitation | -778.14 | 28.88 | 0.002 | 0.125 |
| 1970 (522) | FFD | -1002.58 | 0.00 | 0.113 | 0.193 |
|  | FFD + Precipitation | -1001.43 | 1.15 | 0.115 | 0.187 |
|  | FFD*Precipitation | -999.95 | 2.63 | 0.115 | 0.188 |
|  | Intercept Only | -968.75 | 33.82 | 0.000 | 0.124 |
|  | Precipitation | -966.83 | 35.75 | 0.000 | 0.122 |
| 1975 (561) | FFD | -1040.36 | 0.00 | 0.119 | 0.206 |
|  | FFD + Precipitation | -1039.00 | 1.37 | 0.120 | 0.202 |
|  | FFD*Precipitation | -1038.20 | 2.16 | 0.121 | 0.204 |
|  | Intercept Only | -999.87 | 40.49 | 0.000 | 0.132 |
|  | Precipitation | 997.86 | 42.50 | 0.000 | 0.130 |
| 1980 (610) | FFD | -1127.41 | 0.00 | 0.141 | 0.230 |
|  | FFD + Precipitation | -1126.75 | 0.66 | 0.143 | 0.227 |
|  | FFD*Precipitation | -1126.28 | 1.13 | 0.144 | 0.228 |
|  | Intercept Only | -1074.18 | 53.23 | 0.000 | 0.158 |
|  | Precipitation | -1072.20 | 55.21 | 0.000 | 0.157 |

Table A3.

| Cut-off (n) | Model | AICc | ΔAICc | R^2^m | R^2^c |
| --- | --- | --- | --- | --- | --- |
| 1940 (122) | FFD | 893.98 | 0.00 | 0.366 | 0.689 |
|  | FFD + Precipitation | 896.18 | 2.20 | 0.366 | 0.689 |
|  | FFD*Precipitation | 898.43 | 4.45 | 0.366 | 0.689 |
|  | Intercept Only | 919.77 | 25.80 | 0.000 | 0.562 |
|  | Precipitation | 1173.47 | 279.49 | 0.034 | 0.710 |
| 1945 (175) | FFD | 1170.11 | 0.00 | 0.415 | 0.710 |
|  | FFD + Precipitation | 1172.25 | 2.13 | 0.415 | 0.710 |
|  | FFD*Precipitation | 1174.42 | 4.31 | 0.415 | 0.710 |
|  | Intercept Only | 1208.61 | 38.50 | 0.000 | 0.497 |
|  | Precipitation | 1650.32 | 480.21 | 0.026 | 0.703 |
| 1950 (243) | FFD + Precipitation | 2068.38 | 0.00 | 0.441 | 0.710 |
|  | FFD*Precipitation | 2069.62 | 1.24 | 0.443 | 0.713 |
|  | FFD | 2069.75 | 1.37 | 0.435 | 0.710 |
|  | Intercept Only | 2135.53 | 67.15 | 0.000 | 0.455 |
|  | Precipitation | 2136.43 | 68.05 | 0.000 | 0.448 |
| 1955 (324) | FFD + Precipitation | 2745.06 | 0.00 | 0.605 | 0.713 |
|  | FFD*Precipitation | 2745.99 | 0.93 | 0.607 | 0.718 |
|  | FFD | 2747.53 | 2.47 | 0.600 | 0.712 |
|  | Intercept Only | 2868.77 | 123.71 | 0.000 | 0.197 |
|  | Precipitation | 3325.67 | 580.61 | 0.084 | 0.400 |
| 1960 (375) | FFD*Precipitation | 3145.63 | 0.00 | 0.648 | 0.719 |
|  | FFD | 3147.19 | 1.56 | 0.643 | 0.714 |
|  | Intercept Only | 3293.18 | 147.55 | 0.000 | 0.210 |
|  | FFD + Precipitation | 3446.26 | 300.63 | 0.628 | 0.830 |
|  | Precipitation | 3857.42 | 711.79 | 0.091 | 0.427 |
| 1965 (388) | FFD*Precipitation | 3257.16 | 0.00 | 0.646 | 0.701 |
|  | FFD | 3258.78 | 1.62 | 0.641 | 0.697 |
|  | Intercept Only | 3407.60 | 150.44 | 0.000 | 0.192 |
|  | FFD + Precipitation | 3577.89 | 320.73 | 0.632 | 0.825 |
|  | Precipitation | 3998.12 | 740.96 | 0.097 | 0.402 |
| 1970 (502) | FFD | 4166.20 | 0.00 | 0.644 | 0.684 |
|  | FFD + Precipitation | 4168.17 | 1.97 | 0.644 | 0.685 |
|  | FFD*Precipitation | 4168.39 | 2.19 | 0.646 | 0.688 |
|  | Intercept Only | 4347.44 | 181.24 | 0.000 | 0.172 |
|  | Precipitation | 4349.48 | 183.28 | 0.000 | 0.172 |
| 1975 (550) | FFD | 4528.84 | 0.00 | 0.657 | 0.702 |
|  | FFD + Precipitation | 4530.84 | 2.00 | 0.657 | 0.702 |
|  | FFD*Precipitation | 4531.45 | 2.61 | 0.658 | 0.704 |
|  | Intercept Only | 4734.82 | 205.98 | 0.000 | 0.221 |
|  | Precipitation | 4736.56 | 208.02 | 0.000 | 0.221 |
| 1980 (600) | FFD | 4968.26 | 0.00 | 0.669 | 0.699 |
|  | FFD*Precipitation | 4970.03 | 1.78 | 0.671 | 0.701 |
|  | FFD + Precipitation | 4970.12 | 1.87 | 0.669 | 0.699 |
|  | Intercept Only | 5210.81 | 242.55 | 0.000 | 0.252 |
|  | Precipitation | 5212.57 | 244.57 | 0.000 | 0.252 |

*Sensitivity of change in size and breeding (Julian day) to choice of cut-off date*

In order to determine whether our choice of cut-off date (1960) influenced our results, we compared our estimates of change in body size (female and male) and Julian day from our chosen time periods (1901–1960 to 1961–2000) to estimates achieved by different cut-off years (every 5 years from 1940–1980). For the eight additional cut-off years, we used all available data and determined change between the two periods by subtracting the pre-cut off date data from the post-cut off date data. Differences in estimates with choice of cut-off data were minor (Figures A1-A3), so we kept our original choice of cut-off date (1960) due to supporting evidence from the literature that this was roughly the onset of rapid climate warming.


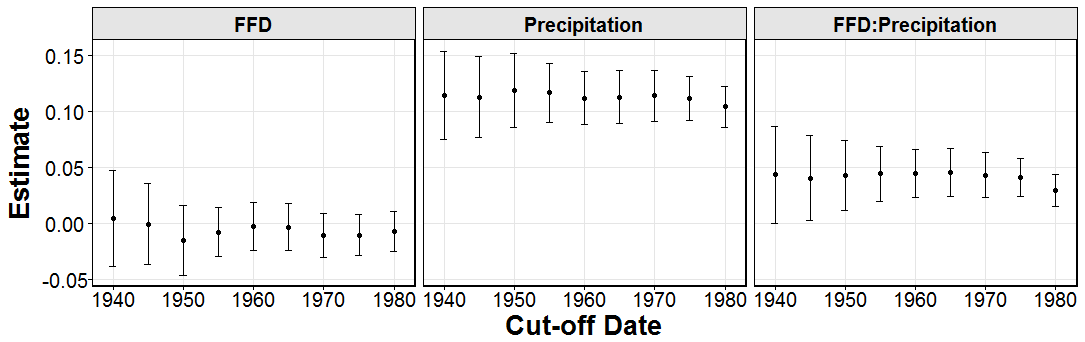


Figure A1. Estimated change in female body size with different cut-off dates for the three climate predictors: FFD, Precipitation, and FFD:Precipitation.


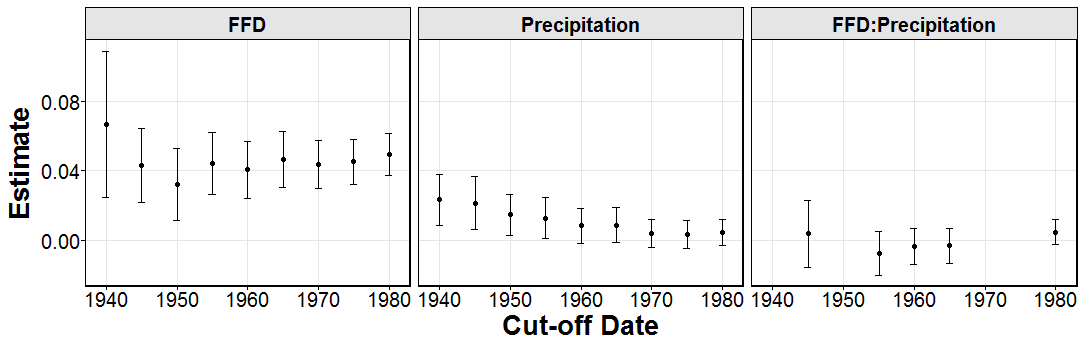


Figure A2. Estimated change in male body size with different cut-off dates for the three climate predictors: FFD, Precipitation, and FFD:Precipitation.


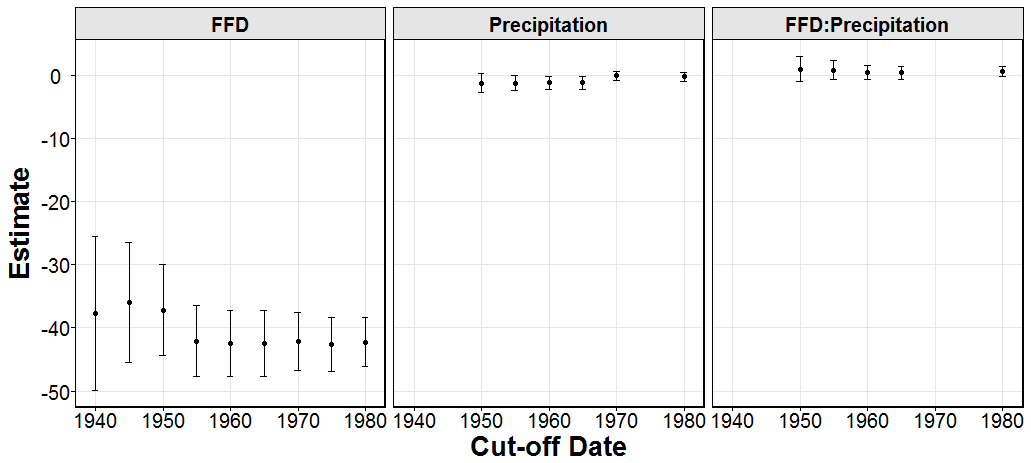


Figure A3. Estimated change in Julian Day of first appearance (breeding) with different cut-off dates for the three climate predictors: FFD, Precipitation, and FFD:Precipitation.
